# Stepwise triple-click functionalization of synthetic peptides†
†Electronic supplementary information (ESI) available. See DOI: 10.1039/c8ob01617h


**DOI:** 10.1039/c8ob01617h

**Published:** 2018-08-09

**Authors:** Anna Kovalová, Radek Pohl, Milan Vrabel

**Affiliations:** a Institute of Organic Chemistry and Biochemistry of the Czech Academy of Sciences , Flemingovo nám. 2 , 16610 , Prague , Czech Republic . Email: vrabel@uochb.cas.cz ; Tel: +420 220183317

## Abstract

The increasing popularity of peptides as promising molecular scaffolds for biomedical applications and as valuable biochemical probes makes new methods allowing for their modification highly desirable. We describe herein an optimized protocol based on a sequence of CuAAC click reactions and selective deprotection steps, which leads to an efficient multi-functionalization of synthetic peptides. The methodology has been successfully applied to the construction of defined heteroglycopeptides and fluorophore–quencher-containing probes for proteases. The developed chemistry thus represents an important addition to the available toolbox of methods enabling efficient postsynthetic modification of peptides. The commercial availability of numerous azide probes further greatly extends the application potential of the described methodology.

## Abstract 2

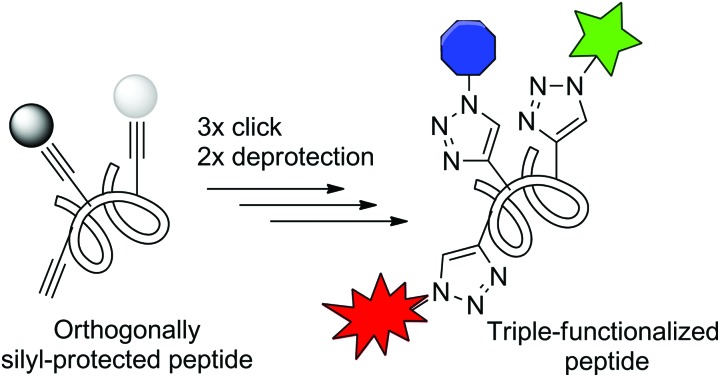
A sequence of click reactions and selective deprotection steps enables the stepwise synthesis of defined triple-functionalized peptides.

## 

The substantial progress made in solid-phase peptide synthesis (SPPS) together with the commercial availability of various building blocks now enables the effective construction of high quality peptides in an automated manner.^1^ The ever growing interest in peptide drugs, often referred to as next-generation therapeutics, led to an increased demand for methods enabling their straightforward and economical production.^2^–^5^ Besides pharmacology, multi-functionalized peptides find broad utility in other fields including peptide arrays,^6^,^7^ materials sciences^8^,^9^ and numerous biological applications.^10^ All of these applications often require the attachment of various functional groups to the peptide backbone. One possibility is to prepare a modified building block, which can be directly incorporated during standard SPPS at the desired position. Despite obvious advantages, this approach often requires laborious synthesis of precious building blocks that need to be used in excess during the coupling reaction step. Alternatively, one can utilize the functional groups present in natural amino acids.^11^–^14^ The major limitation of these methodologies is reflected by the chemical space available within the amino acid side-chains. Although significant selectivity can be achieved for a particular amino acid residue, the fact that these are usually present in multiple copies within a given peptide sequence prevents complete control over the modification site and number of modifications attached.

Incorporation of unnatural functional groups into peptides and proteins, which can be modified by selective chemical reactions, represents an attractive alternative to the abovementioned approaches.^15^,^16^ Among other ligation methods, the Cu-catalyzed azide–alkyne cycloaddition (CuAAC) stands out as one of the most versatile and robust methods that enables selective attachment of useful functional groups to biomolecules.^17^–^19^ The commercial availability of numerous azide and alkyne functional probes further makes this methodology a superior choice for many applications. This powerful reaction, in combination with a shrewd selection of protecting groups, has already found application for the multi-functionalization of nucleic acids,^20^ and for the synthesis of dual labeled proteins^21^ and tri-orthogonal synthetic scaffolds.^22^

Herein, we report on the development of a modular protocol for the selective modification of synthetic peptides based on a sequence of click reactions and deprotection steps. We applied the methodology for the construction of well-defined triple-modified heteroglycopeptides and for the synthesis of fluorogenic protease probes.

Our study began with the synthesis of modified amino acids containing free (**1**) or silyl-protected terminal alkyne groups (**2**, **3**). We chose lysine as the core structure, which was modified at the ε-amino group by 4-pentynoic acid and the corresponding triethylsilyl (TES) or triisopropylsilyl (TIPS) protected derivatives (Scheme 1, Scheme S1 in ESI†).

**Scheme 1:**
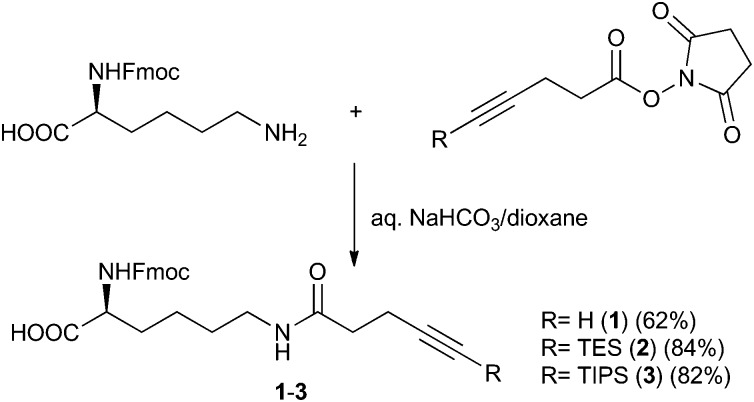
Synthesis of modified amino acids **1–3** containing free or silyl-protected alkyne groups.

We next examined whether these modified amino acids are amenable to standard conditions used in Fmoc SPPS. The synthesis of a model peptide (**Pep1**) was performed on a TentaGel S–OH resin using an automated peptide synthesizer under standard conditions (HBTU = *N*,*N*,*N*′,*N*′-tetramethyl-*O*-(1*H*-benzotriazol-1-yl)uronium hexafluorophosphate as the coupling agent, NMM = 4-methylmorpholine as the base, and 20% piperidine in DMF for Fmoc deprotection). The TentaGel OH resin used allows for easy monitoring of the reaction after cleavage of a small portion of the peptide from the resin using NaOH. A series of experiments confirmed that each of the modified amino acids **1–3** is compatible with the standard SPPS protocols and that these amino acids can be successfully incorporated into peptides (Fig. 1, Scheme S3, Fig. S1–S3†).

**Fig. 1:**
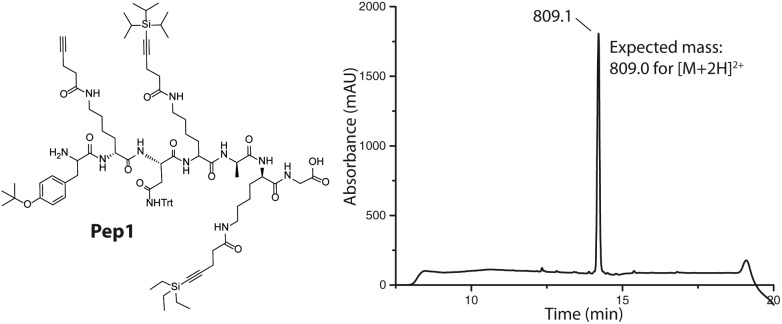
Structure and HPLC chromatogram of the peptide containing all three modified amino acids **1–3** (measured at 214 nm). The observed mass is indicated above the signal of the product.

Our next goal was to identify mild deprotection conditions allowing for selective removal of the TES group in the presence of the TIPS group.^23^ This is an important prerequisite for successful use of the modified silyl-protected amino acids for the intended triple-functionalization of peptides, where each modification is attached in a defined manner using a sequence of click reactions and deprotection steps. Based on our initial experiments, AgNO_3_, AgClO_4_ and AgF were selected as the promising cleavage agents and were studied in more detail (Fig. S6†). Other silver salts were either of limited efficacy (AgNO_2_) or not effective at all (AgOCN, Ag_2_SO_4_) at any given concentrations. We first performed a comprehensive optimization on the modified amino acids and investigated the influence of the silver salt stoichiometry, concentration, and reaction time on the yield of the deprotection step. For this, an equivalent mixture of amino acids **2** and **3** was mixed in DMF/MeOH/H_2_O (60/32/8) and the progress of the reaction was monitored using HPLC/MS analysis. Among other silver salts, AgClO_4_ (2.5 equiv.) gave very clean conversion to the desired TES-deprotected derivative. More importantly, the TIPS group was not affected under these conditions (Fig. 2A and Fig. S6†).

**Fig. 2:**
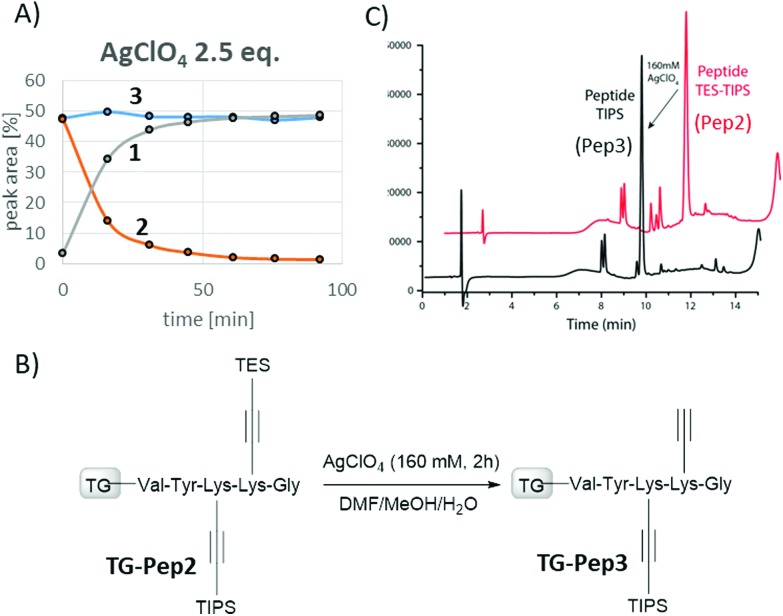
(A) Optimization of the chemoselective TES deprotection using an equimolar mixture of amino acids **2** and **3**. (B) Selective deprotection of the TES group in the presence of the TIPS group on **Pep2**. (C) HPLC analysis (214 nm) of the crude peptide before (red) and after (black) selective TES deprotection using 160 mM AgClO_4_. For specific peptide structures see the ESI.†

Having identified AgClO_4_ as the optimal and chemoselective TES deprotection reagent, we next investigated the deprotection on the model peptide **Pep2**. First, the TES group was successfully removed from the peptide containing amino acid **2** using 160 mM solution of AgClO_4_ (Fig. S7†). Under these conditions, the TIPS group of incorporated amino acid **3** was not affected (Fig. S8†). An experiment on a model peptide containing both amino acids **2** and **3** further confirmed the excellent chemoselectivity of AgClO_4_ (160 mM), which is able to deprotect the TES group in the presence of the TIPS group within two hours giving **Pep3** (Fig. 2B,C and Fig. S9†).

One ongoing project in our laboratory requires specific access to various heteroglycopeptides. Even though several methods to access glycopeptide synthesis exist,^24^,^25^ a robust, efficient and modular approach toward this goal is still missing. We therefore thought to examine our developed methodology for the synthesis of glycopeptides based on selective peptide functionalization by sequential CuAAC reactions. To this end, we first synthesized three different modified sugars, namely: β-d-galactopyranoside (**Gal-C3-N_3_**), β-d-glucopyranoside (**Glc-C4-N_3_**) and α-d-mannopyranoside (**Man-C2-N_3_**) derivatives containing an aliphatic azide moiety at the anomeric position with carbon spacers of various lengths (for details see the ESI†). We then turned our attention to the click reactions.

Unexpectedly we found that the first click reaction performed on **TG-Pep1** still attached to the solid support affords the desired product in low yield giving low conversion under standard click conditions.^26^ Since full conversion of each click-modification step is crucial for obtaining well-defined triple-modified products, we decided to explore this in more detail (Fig. 3). One possible explanation for the observed low reactivity could be attributed to the relatively hydrophobic nature of the peptide (silyl and other protecting groups), which reacts with the hydrophilic azidoglycoside. We therefore decided to investigate the effect of detergents on the reaction efficiency. Tween 20 and Triton X were found to have positive effects on the reaction, but still gave only about 60% conversion. Heating the reaction mixture also did not afford better results. After extensive experimentation we found that performing the reaction in ^*t*^BuOH/H_2_O (2 : 3) in the presence of a base (2.2 equiv. of diisopropylethylamine or *N*-methylmorpholine) led to full conversion of the starting material and the desired click-modified glycopeptide. We speculate that the observed enhanced reactivity of the alkyne groups in CuAAC in the presence of a base could be the result of a base-promoted formation of copper-acetylide species formed as intermediates during the catalytic cycle.^27^ It is important to note that under our optimized conditions the reaction required only two equivalents of the respective azidoglycoside (Fig. 3 and Fig. S10†).

**Fig. 3:**
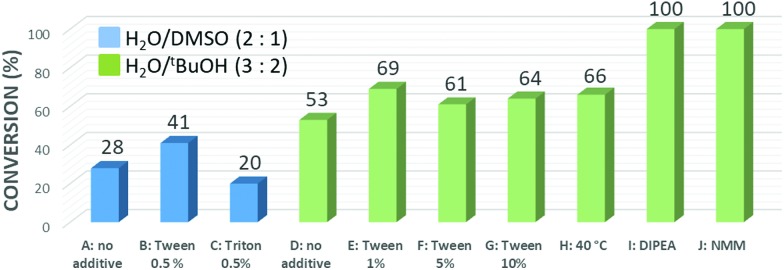
Optimization of the click reaction on **Pep1**. Conditions: **Gal-C3-N_3_** (2 equiv.), 25 mol% CuSO_4_, 50 mol% BTTP ligand, and 50 mol% sodium ascorbate + additive. The conversion was calculated based on HPLC-MS analysis of the cleaved peptide.

Having the optimized conditions for the click reaction in hand we next moved to the triple-click modification of the model peptide using three different azidoglycoside derivatives (Fig. 4 and Fig. S4†). The peptide **Pep4** containing our modified amino acids **1–3** was assembled on the TentaGel NH_2_ resin using standard automated SPPS (see the ESI†). The first C-terminal amino acid was methionine, which enabled cleavage of the peptide from the resin using BrCN.^28^ In this way we were able to follow the progress of the synthesis by removing a small portion of the resin, cleaving the peptide with BrCN and analysing it using HPLC-MS. The first click reaction was performed using **Gal-C3-N_3_** under our optimized conditions. Selective removal of the TES group was achieved with AgClO_4_ giving the free alkyne at position 7. Then, the next **Glc-C4-N_3_** sugar was clicked onto the peptide backbone. The TIPS group was removed using TBAF in THF and the last **Man-C2-N_3_** moiety was introduced again by the optimized click reaction. The peptide backbone was then fully deprotected using a TFA/H_2_O/TIS (95/2.5/2.5) cocktail. We found that during this step a small amount of TFA ester/amide formed (based on MS analysis). This was readily hydrolysed using 1.1 M NaOH(aq). Final cleavage of the peptide from the resin was achieved with BrCN. At this stage the HPLC-MS analysis showed that the whole synthesis of **Pep10** was successful. In fact, the crude reaction mixture after each step, side-chain group deprotection and BrCN cleavage (**Pep5–Pep10**) gave a very clean HPLC profile indicating that all steps proceeded with extraordinary efficiency (Fig. 4B). Our optimized protocol thus enables a straightforward synthesis of heteroglycopeptides by a successive sequence of CuAAC click reaction and deprotection steps.

**Fig. 4:**
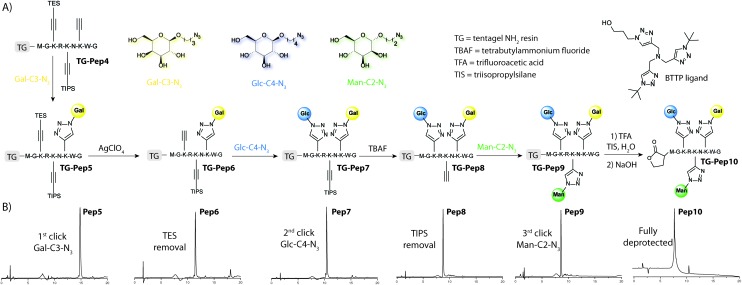
(A) Triple click modification of the model peptide **Pep4** on solid support using a sequence of click reactions/deprotection steps and three different azidoglycosides to give heteroglycopeptide **Pep10**. Click-conditions: azidoglycoside (2 equiv.), CuSO_4_·5H_2_O/BTTP ligand/Na-ascorbate (1/2/2), DIPEA (2.2 equiv.) in ^*t*^BuOH/H_2_O = 2/3 (for details see the ESI†). (B) An HPLC chromatogram of crude reaction mixtures after each step (266 nm). For specific peptide structures see the ESI.†

To further evaluate our strategy for the synthesis of defined, multi-functionalized peptide probes, we decided to prepare a fluorophore–quencher-modified peptide. Such modified peptides are very useful probes for studies related to substrate specificity of various proteases and in drug discovery.^29^ As a proof of principle, we chose trypsin, a well-known and established serine protease, which cleaves the peptide bonds after polar lysine (K) and arginine (R) residues. Toward this end, we prepared a peptide YK(alk)KAFK(alk-TES)MG by automated SPPS (ESI†). The peptide still attached to the solid support was first modified with 7-hydroxy-3-azidocoumarin, a fluorogenic probe that becomes fluorescent upon CuAAC.^30^ The TES group was subsequently removed using AgClO_4_. We used a deep red-coloured azide-modified azobenzene quencher in the following click reaction step. The peptide side-chain protecting groups were removed with TFA/H_2_O/TIS solution and the final doubly modified peptide was cleaved from the resin using BrCN (see the ESI†). HPLC-MS analysis confirmed the presence of the desired fluorogenic peptide probe **Pep11** (Fig. S5†). The presence of the two molecules, the quencher and the fluorophore, within one peptide chain results in quenching of the coumarin fluorescence due to fluorescence resonance energy transfer. Cleavage of the peptide bond by trypsin after the lysine at position 4 results in spatial separation of the two molecules (**Pep12** and **Pep13**) and restoration of the fluorescence (Fig. 5). Indeed, when we incubated the modified peptide probe **Pep11** with trypsin and followed the reaction progress on a fluorescence plate reader we observed a time-dependent increase in fluorescence (Fig. S11†). This proof-of-principle experiment demonstrates that our methodology can be successfully applied for constructing fluorogenic protease probes. One particular advantage of our approach is that it allows for easy optimization of the fluorophore–quencher pair for particular application. This can be done by simply changing the clickable azide probes, many of which are nowadays commercially available.

**Fig. 5:**
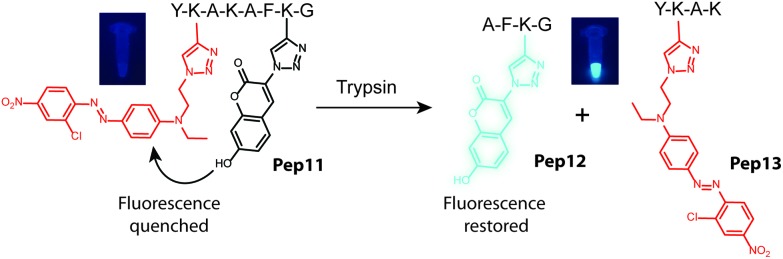
Fluorogenic protease probe **Pep11** containing the quenched coumarin dye (black) in the presence of the azobenzene quencher (red). Incubation with trypsin leads to restoration of the coumarin fluorescence (blue) indicating successful peptide cleavage. For specific peptide structures see the ESI.†

In conclusion, we describe a robust protocol enabling selective, triple-modification of peptides, which is based on the incorporation of orthogonally silyl-protected amino acids compatible with standard Fmoc solid-phase peptide synthesis. A subsequent optimized sequence of click reactions/deprotection steps enables efficient, modular and versatile attachment of various functional groups to synthetic peptides in a defined manner. The commercial availability of numerous azides (fluorophores, PEG linkers, pull-down and targeting probes, quenchers, sugars *etc*.) makes the presented procedure a readily viable methodology for constructing functionalized peptides useful in various applications.

## Conflicts of interest

There are no conflicts to declare.

## Supplementary Material

Supplementary information
