# Correction: Stepwise triple-click functionalization of synthetic peptides

**DOI:** 10.1039/c8ob90127a

**Published:** 2018-09-05

**Authors:** Anna Kovalová, Radek Pohl, Milan Vrabel

**Affiliations:** Institute of Organic Chemistry and Biochemistry of the Czech Academy of Sciences Flemingovo nám. 2 16610 Prague Czech Republic vrabel@uochb.cas.cz +420 220183317

## Abstract

Correction for ‘Stepwise triple-click functionalization of synthetic peptides’ by Anna Kovalová *et al.*, *Org. Biomol. Chem.*, 2018, **16**, 5960–5964.

The authors regret that the author list for reference 22 was included incorrectly. The correct reference is shown below.

22 J. Pícha, B. Fabre, M. Buděšínský, J. Hajduch, M. Abdellaoui and J. Jiráček, *Eur. J. Org. Chem.*, 2018, DOI: 10.1002/ejoc.201800601.

The Royal Society of Chemistry apologises for these errors and any consequent inconvenience to authors and readers.

## Supplementary Material

