# Supplementary material for: Stepwise triple-click functionalization of synthetic peptides
Source: Org Biomol Chem. 2018 Aug 9;16(33):5960–4. doi: 10.1039/c8ob01617h (PMC6113709; doi:10.1039/c8ob01617h)
Supplement: Supplementary file 1 [file OB-016-C8OB01617H-s001.pdf]

Electronic Supplementary Material (ESI) for Organic & Biomolecular Chemistry.
This journal is © The Royal Society of Chemistry 2018

Supporting Information

Table of Contents

General information 3

Synthetic procedures 3

Compounds prepared according to literature: 3

AA building blocks synthesis: 4

General procedure 1 (for synthesis of **5** and **6**): 4

tert-Butyl 5-(triisopropylsilyl)pent-4-ynoate (**5**) 5

tert-Butyl 5-(triethylsilyl)pent-4-ynoate (**6**) 5

General procedure 2 (for synthesis of **9**, **10**, and **11**) 5

2,5-dioxopyrrolidin-1-yl pent-4-ynoate[6] (pent-4-ynoic acid 1-oxysuccinimidyl ester (**11**) 5

2,5-dioxopyrrolidin-1-yl 5-(triisopropylsilyl)pent-4-ynoate (TIPS-pent-4-ynoic acid 1-oxysuccinimidyl ester (**9**) 6

2,5-dioxopyrrolidin-1-yl 5-(triethylsilyl)pent-4-ynoate (TES-pent-4-ynoic acid 1-oxysuccinimidyl ester (**10**) 6

General procedure 3 (for synthesis of **1**, **2** and **3**) 6

N^2-(((9H-fluoren-9-yl)methoxy)carbonyl)-N^6-(pent-4-ynoyl)-L-lysine (Fmoc-L-Lys(pentynoyl)-OH, (**1**) 7

N^2-(((9H-fluoren-9-yl)methoxy)carbonyl)-N^6-(5-(triisopropylsilyl)pent-4-ynoyl)-L-lysine ((Fmoc-L-Lys(pentynoyl-TIPS)-OH, (**2**) 7

N^2-(((9H-fluoren-9-yl)methoxy)carbonyl)-N^6-(5-(triethylsilyl)pent-4-ynoyl)-L-lysine ((Fmoc-L-Lys(pentynoyl-TES)-OH, **3**) 8

Saccharide building blocks synthesis: 9

(2R,3R,4S,5R,6R)-2-(acetoxymethyl)-6-(4-bromobutoxy)tetrahydro-2H-pyran-3,4,5-triyl triacetate (4-Bromobutyltetra-O-acetyl-β-D-glucopyranoside (**12**)) 10

General procedure 4 (for synthesis of **13**, **15** and **17**) 11

(2R,3R,4S,5R,6R)-2-(acetoxymethyl)-6-(4-azidobutoxy)tetrahydro-2H-pyran-3,4,5-triyl triacetate (**13**) 11

(2R,3R,4S,5S,6S)-2-(acetoxymethyl)-6-(2-azidoethoxy)tetrahydro-2H-pyran-3,4,5-triyl triacetate (**17**) 11

(2R,3S,4S,5R,6R)-2-(acetoxymethyl)-6-(3-azidopropoxy)tetrahydro-2H-pyran-3,4,5-triyl)triacetate (**15**) 12

General procedure 5 (for synthesis of **Glc-C4-N$_3$**, **Gal-C3-N$_3$** and **Man-C2-N$_3$**) 12

(2S,3S,4S,5S,6R)-2-(2-azidoethoxy)-6-(hydroxymethyl)tetrahydro-2H-pyran-3,4,5-triol (**Man-C2-N$_3$**) 13

(2R,3R,4S,5R,6R)-2-(3-azidopropoxy)-6-(hydroxymethyl)tetrahydro-2H-pyran-3,4,5-triol (**Gal-C3-N_3**) 13
Prepration of model peptides **Pep14** and **Pep15**: 13
Resin loading: 14
Automated SPPS 15
Prepration of model peptide **Pep16**: 17
Resin loading: 17
SPPS 17
Synthesis of model modified peptide **Pep1** 19
Synthesis of glycosylated peptide **Pep10**: 21
Resin loading: 22
Automated SPPS 22
Standard protocols for SP3 Peptide Synthesizer: 23
General procedure 7: Cleavage-off of TentaGel-S-NH_2 resin with CNBr (for analytical samples) 23
Modification of resin-bound protected peptide **Pep4** 23
Click I 23
TES removal: 24
Click II 24
TIPS removal: 25
Click III 25
Deprotection of the modified peptide: 26
Synthesis of **Pep11** 28
Resin loading: 28
Automated SPPS 29
Modification of resin-bound protected peptide 29
Click I – 3-azido-7-hydroxycoumarin 29
TES removal: 30
Click II - N-ethyl-N-(2-azidoethyl)-4-(2-chloro-4-nitrophenylazo)phenylamine 30
Deprotection of the modified peptide: 31
Cleavage-off from the resin: 31
Synthesis of **18** – the positive standard for fluorescence assay 32
Methods optimizations: 33
Optimization of TES removal in the presence of TIPS: 33
Optimization of TES removal from resin bound peptides: 35
Optimization of CuAAC reaction for resin-bound peptides: 39

Typical procedure: 39
Fluorescence assay – Cleavage of fluorogenic substrate **Pep11** by trypsin 40
Conditions: 41
Copies of NMR spectra 43
Compound **4** 43
Compound **7** 44
Compound **8** 45
Compound **16** 46
Compound **19** 47
Compound **5** 48
Compound **6** 49
Compound **11** 50
Compound **9** 51
Compound **10** 52
Compound **1** 53
Compound **2** 54
Compound **3** 55
Compound **12** 56
Compound **14** 57
Compound **13** 58
Compound **17** 59
Compound **15** 60
Compound **Glc-C4-N_3** 61
Compound **Man-C2-N_3** 62
Compound **Gal-C3-N_3** 63
Abbreviations 64
References: 65

General information

The chemicals were obtained from *Sigma Aldrich*, *Alfa Aesar*, *Acros Organics*, *ABCR, Flouorochem, Iris Biochem, Carbosynth* or *VWR* and were used without further purification. Reactions with air- and moisture-sensitive reactants were performed under argon atmosphere and in anhydrous solvents.

Solutions were concentrated on a rotary evaporator from *Heidolph* equipped with a PC3001 VARIOpro pump from *Vacuubrand.* Column chromatography was carried out on silica gel 60Å (particle size: 40-60 µm) from *Acros Organics*. Solvents in the p.a. quality from *Lach-Ner* and *Penta* were used for elution. Mixtures of solvents are each stated as volume fractions. For flash column chromatography a Combi*Flash*® Rf+ from *Teledyne ISCO* was used. Thin-layer chromatography was performed on aluminum sheets from *Merck* (silica gel 60 F254, 20 × 20 cm). Chromatograms were visualized by UV light (λ = 254 nm/ 366 nm) or by staining with $KMnO_4$ solution, $(NH_4)_2Ce(NO_3)_6/(NH_4)_6Mo_7O_{24}*4H_2O$ solution, or PPh_3/ninhydrin method. For amino acid analysis or determination of resin loading, samples were hydrolyzed in 6 M HCl at 110~C overnight and analyzed on a Biochrom 30 amino acid analyzer (*Biochrom Ltd.,* UK). ^{1}H- and ^{13}C-NMR spectra were measured on a Bruker Avance III™ HD 400 MHz NMR system equipped with Prodigy cryo-probe or on a Bruker Avance III™ HD 500 MHz Cryo. $CDCl_3$, methanol-d_4 deuterium oxide and DMSO-d_6 from *Sigma Aldrich* or *Eurisotop* were used as solvents. Chemical shifts δ are quoted in ppm in relation to the chemical shift of the residual non-deuterated solvent peak ($CDCl_3$: $\delta(^1H) = 7.26$, $\delta(^{13}C) = 77.2$; methanol-d4: $\delta(^1H) = 4.87$, $\delta(^{13}C) = 49.0$; DMSO-d_6: $\delta(^1H) = 2.50$, $\delta(^{13}C) = 39.5$; deuterium oxide: $\delta(^1H) = 4.79$). *J* values are given in Hz. High-resolution mass spectra were recorded on an *Agilent* 5975C MSD Quadrupol, Q-Tof micro from *Waters* or LTQ Orbitrap XL from *Thermo Fisher Scientific*. HPLC-MS measurements were performed either on an LCMS-2020 system from *Shimadzu* equipped with Luna® C18(2) column (3µm, 100A, 100 × 4.6 mm), or on HPLC-MS Infinity 1260 system equipped with 6120 Quadrupole LC/MS detector from *Agilent Technologies* and either preparative column Luna® 5 µm C18 (2), 100 Å, 250 x 21.2 mm (Phenomenex) or analytical column Poroshell 120, EC-C18 4 µm, 4.6 x 100 mm *(Agilent Technologies)*. UV/VIS spectroscopy was performed on a Cary 60 UV/Vis spectrophotometer from *Agilent Technologies*. Data from experiments were processed using Microsoft Excell 2016 MSOs software. Fluorescence measurements were performed on Spark® microplate reader from *Tecan,* in 96-well half area black polystyrene microplates *(Corning)*. Automated peptide synthesis was done on PS3™ Peptide Synthesizer, *Protein Technologies, Inc.*. For microwave irradiation, the standard kitchen microwave oven Daewoo KOR-9GPBC (*Daewoo Electronics*) was used.

Synthetic procedures

Compounds prepared according to literature:

tert-Butyl-4-pentynoate[1, 2] (**4**); HRMS $[M+H]^+$ m/z calcd. for $[C_9H_{15}O_2]^+$ 155.1072, found 155.1077

5-(Triisopropylsilyl)-4-pentynoic acid[3] (**7**); HRMS $[M+H]^+$ m/z calcd. for $[C_{14}H_{25}O_2Si]^+$ 253.16293, found 253.16335

5-(Triethylsilyl)-4-pentynoic acid[3] (**8**); HRMS $[M+H]^+$ m/z calcd. for $[C_{11}H_{19}O_2Si]^+$ 211.11598, found 211.11591

2'-Bromoethyl-2,3,4,6-tetra-O-acetyl-α-D-mannopyranoside[4] (**16**); HRMS $[M+Na]^+$ m/z calcd. for $[C_{16}H_{23}O_{10}BrNa]^+$ 477.03668, found 477.03668

(E)-N-(2-azidoethyl)-4-((2-chloro-4-nitrophenyl)diazenyl)-N-ethylaniline[5] (**19**) HRMS $[M+H]^+$ m/z calcd. for $[C_{16}H_{17}O_2N_7Cl]^+$ 374.11268, found 374.11253

AA building blocks synthesis:

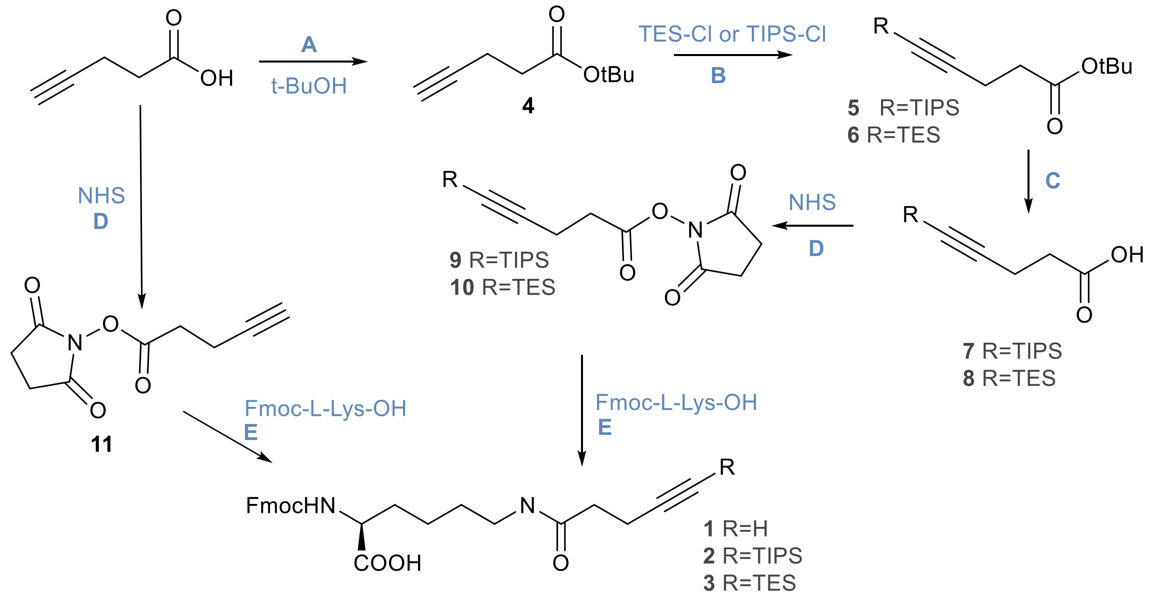

A: DCC, DMAP, DCM, r.t., 15h, 74% - 99%; **B**: n-BuLi, dry THF, -78°C-0°C-r.t., 3.5h, 70% (TIPS), 29% (TES); **C**: 15% TFA in dry DCM, r.t., 74% (TIPS), 30% (TES); **D**: DCC, dry THF, 0°C 40 min, r.t. 2-4.5h, 86% (free alkyne), 79% (TIPS), 64% (TES); **E**: dioxane/NaHCO3 aq. sat, 0°C-r.t., 17-20h, 62% (free alkyne), 84% (TIPS), 72% (TES).

Scheme S1: Synthesis of amino acid building blocks

General procedure 1 (for synthesis of 5 and 6):

According to lit.[3], with slight modifications. Alkyne was taken into dry THF and cooled to -78 °C in acetone/dry ice bath. The solution of n-BuLi (2.5M in hexane; 1.3 eq.) was added slowly and reaction mixture was stirred for 10 min. The cooling bath was replaced with ice bath (0 °C) and Silyl-Cl (1.2 eq.) was added dropwise. The reaction proceeded for 3 hrs at r.t., was quenched with sat. aq. NH_4Cl, THF was gently evaporated, resulting slurry diluted with water, extracted with EtOAc, and concentrated. The crude product was purified by column chromatography.

*tert-Butyl 5-(triisopropylsilyl)pent-4-ynoate (**5**)*

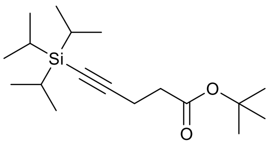

The general procedure 1 was followed, using 13 mmol of **4**. The reaction was quenched with sat. aq. NH_4Cl (80 ml), THF was removed, resulting slurry diluted with water (100 ml), extracted with EtOAc (3 x 60 ml) and purified by silicagel column using PE-Et_2O (50 : 1). Yield: 2.8 g, 70 %. NMR data correspond to lit.[3]; HRMS $[M+Na]^+$ m/z calcd. for $[C_{18}H_{34}O_2NaSi]^+$ 333.22203, found 333.22212.

*tert-Butyl 5-(triethylsilyl)pent-4-ynoate (**6**)*

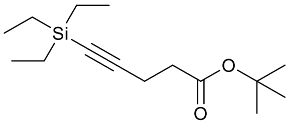

The general procedure 1 was followed, using 43 mmol of **4**. The reaction was quenched with sat. aq. NH_4Cl (160 ml), THF was removed, resulting slurry diluted with water (200 ml), extracted with EtOAc (3 x 100 ml) and purified by silicagel column using PE-Et_2O (50 : 1). Yield: 3.3 g, 29 %. NMR data correspond to lit.[3]; HRMS $[M+Na]^+$ m/z calcd. for $[C_{15}H_{28}O_2NaSi]^+$ 291.17508, found 291.17531, 292.17898.

General procedure 2 (for synthesis of **9**, **10**, and **11**)

According to lit.[6], with slight modifications. To the solution of acid and N-hydroxy succinimide (1.05 eq.) in dry THF at 0 °C DCC (1.05 eq.) dissolved in dry THF was added slowly. The mixture was stirred for 40 min. at 0 °C, warmed gradually to r.t. and stirred for additional 2h. The reaction mixture was filtered, concentrated, dissolved in EtOAc, filtered, washed with saturated solution of $NaHCO_3$ and brine, and dried over Na_2SO_4. The residue was swiftly purified on column of silica.

*2,5-dioxopyrrolidin-1-yl pent-4-ynoate[6] (pent-4-ynoic acid 1-oxysuccinimidyl ester (**11**)*

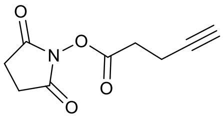

The general procedure 2 was followed, using 4 mmol of 4-pentynoic acid in dry THF (14 ml) and DCC. For the workup, the reaction mixture was dissolved in EtOAc (100 ml), filtered, washed with $NaHCO_3$ sat. (30 ml) and brine (30 ml), dried over Na_2SO_4 and swiftly purified on column of silica in EtOAc - PE (1:1). Yield 670 mg, 86 %. Analytical data corresponded to lit.[6]

*2,5-dioxopyrrolidin-1-yl 5-(triisopropylsilyl)pent-4-ynoate (TIPS-pent-4-ynoic acid 1-oxysuccinimidyl ester (**9**)*

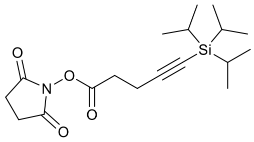

The general procedure 2 was followed, using 0.94 mmol of **7** in dry THF (3 ml). For the workup, the reaction mixture was dissolved in EtOAc (100 ml), filtered, washed with $NaHCO_3$ sat. (30 ml) and brine (30 ml), dried over Na_2SO_4 and swiftly purified on column of silica in EtOAc – PE (1 : 3). Yield 263 mg, 79 %.

δ_H (400 MHz, Chloroform-*d*) 2.92 – 2.84 (2 H, m, OC(O)C$\boldsymbol{H_2}$CH$_2$), 2.83 (4 H, s, NC(O)C$\boldsymbol{H_2}$C$\boldsymbol{H_2}$), 2.71 – 2.61 (2 H, m, OC(O)CH$_2$C$\boldsymbol{H_2}$), 0.97 (9 H, t, *J* 7.9, CH$_3$), 0.57 (6 H, qd, *J* 7.9, 0.6, C$\boldsymbol{H_2}$CH$_3$).

δ_C (101 MHz, Chloroform-*d*) 169.01 (N***C***(O)CH$_2$), 167.15 (O***C***(O)CH$_2$CH$_2$), 105.02 (SiC≡***C***), 82.56 (Si***C***≡C), 30.99 (OC(O)CH$_2$***C***H$_2$), 25.71 (NC(O)CH$_2$CH$_2$), 18.70 (6C, CH$_3$), 15.60 (OC(O)***C***H$_2$CH$_2$), 11.29 (***C***H$_2$CH$_3$).

HRMS [M+Na]$^+$ m/z calcd. for [$C_{18}H_{29}NNaO_4Si$]$^+$ 374.17581, found 374.17597.

*2,5-dioxopyrrolidin-1-yl 5-(triethylsilyl)pent-4-ynoate (TES-pent-4-ynoic acid 1-oxysuccinimidyl ester (**10**)*

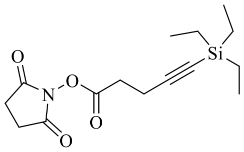

The general procedure 2 was followed, using 0.75 mmol of **8** in dry THF (4 ml). For the workup, the reaction mixture was dissolved in EtOAc (100 ml), filtered, washed with $NaHCO_3$ sat. (30 ml) and brine (30 ml), dried over Na_2SO_4 and swiftly purified by flash chromatograply on silica in DCM - MeOH (200:1). Yield 148 mg, 64 %.

δ_H (400 MHz, Chloroform-*d*) 2.94 – 2.87 (2 H, m, OC(O)C$\boldsymbol{H_2}$CH$_2$)), 2.85 (4 H, s, NC(O)C$\boldsymbol{H_2}$C$\boldsymbol{H_2}$), 2.72 – 2.64 (2 H, m, OC(O)CH$_2$C$\boldsymbol{H_2}$), 0.99 (9 H, t, *J* 7.9, CH$_3$), 0.65 – 0.54 (6 H, m, C$\boldsymbol{H_2}$CH$_3$).

δ_C (101 MHz, Chloroform-*d*) 169.00 (N***C***(O)CH$_2$), 167.16 (O***C***(O)CH$_2$CH$_2$), 104.40 (SiC≡***C***), 83.86 (Si***C***≡C), 30.86 (OC(O)***C***H$_2$CH$_2$), 25.70 (NC(O)***C***H$_2$CH$_2$), 15.58 (OC(O)CH$_2$***C***H$_2$), 7.53 (CH$_3$), 4.47 (***C***H$_2$CH$_3$).

HRMS [M+Na]$^+$ m/z calcd. for [$C_{15}H_{23}NNaO_4Si$]$^+$ 332.12902, found 332.12886.

General procedure 3 (for synthesis of **1**, **2** and **3**)

A suspension of Fmoc-L-Lys-OH in the mixture of dioxane and sat. aq. $NaHCO_3$ (1 : 3) was cooled to 0 °C. A solution of active ester (1.2 eq.) in dioxane was added dropwise. In 5 min the ice bath was removed and reaction proceeded at r.t. for 20 hrs. Reaction mixture was diluted with EtOAc, washed

with sat. aq. citric acid, water, brine and dried over Na_2SO_4. Concentrated residue was purified by flash chromatography on C18 phase and lyophilized.

*N²-(((9H-fluoren-9-yl)methoxy)carbonyl)-N⁶-(pent-4-ynoyl)-L-lysine (Fmoc-L-Lys(pentynoyl)-OH, (**1**)*

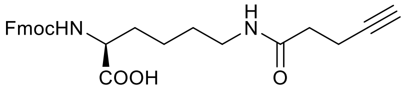

The general procedure 3 was followed, using 0.54 mmol of Fmoc-*L*-Lys-OH in the mixture of dioxane and sat. aq. $NaHCO_3$ (1 : 3; 4 ml) and **11** in dioxane (2 ml). For the workup, the reaction mixture was diluted with EtOAc (30 ml), washed with sat. aq. citric acid (20 ml), water (20 ml), brine (20 ml) and dried over Na_2SO_4. Concentrated residue was purified by flash chromatography on C18 phase in H_2O-MeOH (5 →95 %). Yield: 152 mg, 62 %.
Analytical data correspond to lit.[7]; HRMS $[M+Na]^+$ m/z calcd. for $[C_{26}H_{28}N_2NaO_4Si]^+$ 471.18904, found 471.18865.

*N²-(((9H-fluoren-9-yl)methoxy)carbonyl)-N⁶-(5-(triisopropylsilyl)pent-4-ynoyl)-L-lysine ((Fmoc-L-Lys(pentynoyl-TIPS)-OH, (**2**)*

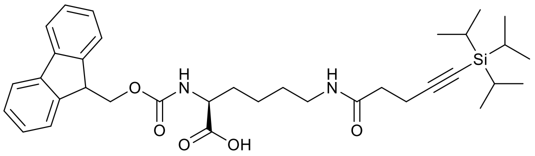

The general procedure 3 was followed, using 1.81 mmol of Fmoc-*L*-Lys-OH in the mixture of dioxane and sat. aq. $NaHCO_3$ (1 : 3, 12 ml) and **9** in dioxane (6 ml). For the workup, the reaction mixture was diluted with EtOAc (100 ml), washed with sat. aq. citric acid (25 ml), water (25 ml), brine (25 ml) and dried over Na_2SO_4. Concentrated residue was purified by flash chromatography on C18 phase in H_2O - ACN (60 →90 % 20 min, 90 % 15 min). Yield: 1.1 g, 84 %.

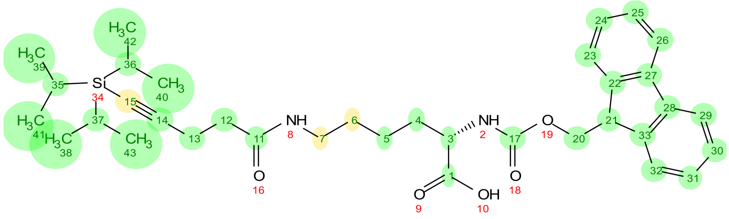

δ_H (400 MHz, Methanol-d_4) 7.78 (2 H, d, *J* 7.5, 26, 29), 7.67 (2 H, t, *J* 6.7, 25, 30), 7.42 – 7.34 (2 H, m, 23, 32), 7.30 (2 H, td, *J* 7.5, 1.2, 24, 31), 4.87 (7 H, s), 4.44 – 4.33 (1 H, m), 4.34 (1 H, d, *J* 2.2, 20), 4.22 (1 H, t, *J* 7.0, 21), 4.13 (1 H, dd, *J* 9.4, 4.6, 3), 3.30 (3 H, p, *J* 1.6), 3.17 (2 H, t, *J* 6.9, 7', 7''), 2.55 (2 H, t, *J* 7.2, 13', 13''), 2.37 (2 H, t, *J* 7.2, 12', 12''), 1.91 – 1.79 (1 H, m, 4), 1.70 (1 H, dtd, *J* 14.1, 9.2, 5.5, 4), 1.50 (3 H, dtd, *J* 37.6, 14.3, 13.7, 6.9, 5', 5''), 1.46 (1 H, s, 6', 6''), 1.04 (18 H, d, *J* 6.0, 38, 39, 40, 41, 42, 43), 1.01 – 0.91 (2 H, m).

δ_C (101 MHz, Methanol-d_4) 175.95, 173.91, 145.19, 142.59, 128.78, 128.17, 128.14, 126.28, 126.25, 120.91, 67.96, 55.19, 48.42, 40.24, 36.35, 32.31, 29.91, 24.34, 19.05, 17.10, 12.43.

HRMS $[M+Na]^+$ m/z calcd. for $[C_{35}H_{48}N_2NaO_5Si]^+$ 627.32247, found 627.32270.

*N^2-(((9H-fluoren-9-yl)methoxy)carbonyl)-N^6-(5-(triethylsilyl)pent-4-ynoyl)-L-lysine ((Fmoc-L-Lys(pentynoyl-TES)-OH, **3**)*

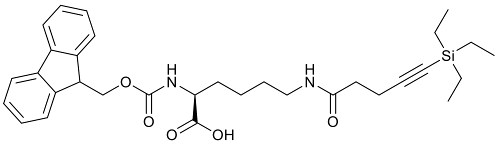

The general procedure 3 was followed, using 2.72 mmol of Fmoc-*L*-Lys-OH in the mixture of dioxane and sat. aq. $NaHCO_3$ (1 : 3, 20 ml) and **10** in dioxane (10 ml). For the workup, the reaction mixture was diluted with EtOAc (100 ml), washed with sat. aq. citric acid (25 ml), water (25 ml), brine (25 ml) and dried over Na_2SO_4. Concentrated residue was purified by flash chromatography on reverse phase in H_2O – ACN (60 $\rightarrow$90 % 20 min, 90 % 15 min). Yield: 1.1 g, 72 %.

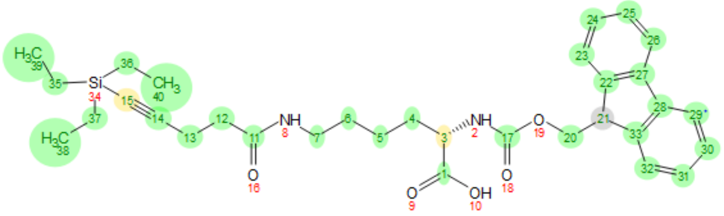

δ_H (400 MHz, Methanol-*d*$_4$) 7.78 (2 H, d, *J* 7.5, 26, 29), 7.67 (2 H, t, *J* 6.7, 25, 30), 7.42 – 7.34 (2 H, m, 23, 32), 7.30 (2 H, td, *J* 7.4, 1.2, 24, 31), 4.44 – 4.33 (1 H, m), 4.34 (1 H, d, *J* 2.4, 20), 4.22 (1 H, t, *J* 7.0, 21), 4.13 (1 H, dd, *J* 9.4, 4.6, 3), 3.17 (2 H, t, *J* 6.9, 7), 2.52 (2 H, t, *J* 7.2, 13), 2.36 (2 H, t, *J* 7.2, 12), 1.91 – 1.79 (1 H, m, 4''), 1.70 (1 H, dtd, *J* 14.1, 9.3, 5.7, 4'), 1.61 – 1.36 (3 H, m, 5'', 6''), 0.96 (9 H, t, *J* 7.9, 38, 39, 40), 0.53 (6 H, q, *J* 7.9, 35'', 36'', 37'').

δ_C (101 MHz, Methanol-*d*$_4$) 175.97 , 1, 173.96 , 11, 158.71 , 17, 145.35 , 22, 33, 145.19, 142.59 , 27, 28, 128.78 , 25, 30, 128.17 , 24, 31, 128.14, 126.28, 126.25 , 23, 32, 120.91 , 26, 29, 107.93 , 15, 82.86, 14, 67.95 , 20, 55.20 , 3, 48.42 , 21, 40.16 , 7, 36.28 , 12, 32.29 , 4, 29.94 , 6, 24.31 , 5, 17.14 , 13, 7.78 , 38, 39, 40, 5.38 , 35, 36, 37.

HRMS $[M+Na]^+$ m/z calcd. for $[C_{32}H_{42}N_2NaO_5Si]^+$ 585.27552, found 585.27595.

Saccharide building blocks synthesis:

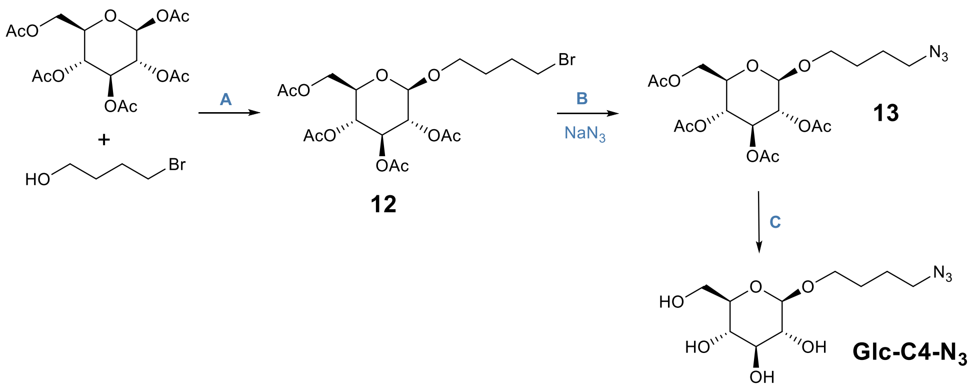

A: $BF_3.Et_2O$, dry DCM, r.t., 2.5h, 16%; **B**: NaN_3, $[Bu_4N]^+Br^-$, DMSO, r.t., 19.5h, 97%;
C: DowexR 1x8, MeOH, r.t., 16h, 93%.

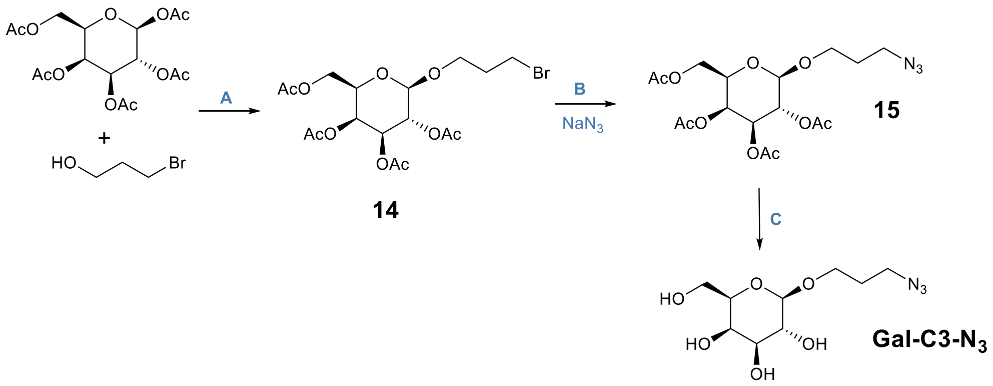

A: $BF_3.Et_2O$, dry DCM, 0°C-r.t, 23h, 50% **B**: NaN_3, $[Bu_4N]^+$Br-, DMSO, r.t., 29h, 90%;
C: DowexR 1x8, MeOH, r.t., 16h, 87%.

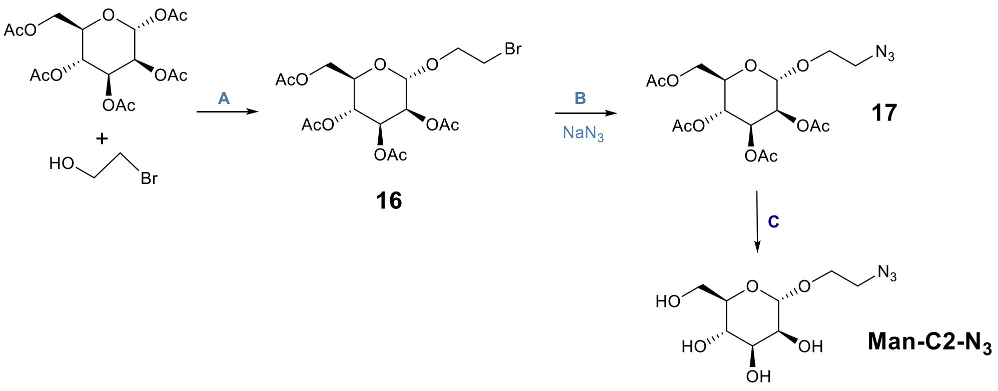

A: $BF_3.Et_2O$, dry DCM, 0°C-r.t, 23h, 67% **B**: NaN_3, $[Bu_4N]^+$Br-, DMSO, r.t., 23.5h, 99%; C: DowexR 1x8, MeOH, r.t., 16h, 84%.

Scheme S2: Synthesis of saccharide building blocks

(2R,3R,4S,5R,6R)-2-(acetoxymethyl)-6-(4-bromobutoxy)tetrahydro-2H-pyran-3,4,5-triyl triacetate (4-Bromobutyltetra-O-acetyl-β-D-glucopyranoside (12))

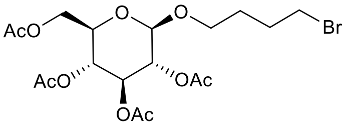

Procedure according to lit.[8], with slight modifications. To the solution of (2S,3R,4S,5R,6R)-6-(acetoxymethyl)tetrahydro-2H-pyran-2,3,4,5-tetrayl tetraacetate (3.9 g; 10 mmol) and 4-bromobutan-1-ol (2.15 ml; 15 mmol) in dry DCM (20 ml) boron trifluoride diethyl etherate (6.17 ml; 50 mmol) was added slowly under argon. After 74.5 hrs at room temperature the reaction mixture was poured into ice-cold H_2O (40 ml) and extracted with DCM (3 x 20 ml). Organic layer was washed with H_2O (25 ml), $NaHCO_3$ sat. (30 ml), H_2O (30 ml), and dried over Na_2SO_4.The product was purified on column of silica in DCM-MeOH (200 : 1) and by crystallization from iPrOH. Yield: 778 mg, 16 %.

δ_H (400 MHz, Chloroform-*d*) 5.20 (1 H, t, *J* 9.5, 2-H), 5.12 – 5.03 (1 H, m, 4-H), 4.98 (1 H, dd, *J* 9.6, 7.9, 3-H), 4.49 (1 H, d, *J* 7.9, 1-H), 4.19 (2 H, ddd, 6-$H_{a,b}$), 3.94 – 3.87 (1 H, m, OC$\boldsymbol{H_a}$$H_b$$CH_2$), 3.69 (1 H, ddd, *J* 10.0, 4.8, 2.5, 5-H), 3.57 – 3.48 (1 H, m, O$CH_a$$\boldsymbol{H_b}$$CH_2$), 3.45 – 3.37 (2 H, m, $CH_2$$\boldsymbol{CH_2}$Br), 2.08 (3 H, s, CH_3), 2.05 (3 H, s, CH_3), 2.02 (3 H, s, CH_3), 2.00 (3 H, s, CH_3), 1.96 – 1.86 (2 H, m, $\boldsymbol{CH_2}$$CH_2$Br), 1.78 – 1.68 (2 H, m, O$CH_a$$H_b$$\boldsymbol{CH_2}$).

δ_C (101 MHz, Chloroform-*d*) 170.82 (C=O), 170.43 (C=O), 169.54 (C=O), 169.44 (C=0), 100.87 (CH-1), 72.94 (CH-2), 71.94 (CH-5), 71.40 (CH-3), 69.06 (O**C**$H_2$$CH_2$), 68.55 (CH-4), 62.08 ($CH_2$-6), 33.45 ($CH_2$**C**$H_2$Br), 29.39 (**C**$H_2$$CH_2$Br), 28.12 (O$CH_2$**C**$H_2$), 20.90 ($CH_3$), 20.83 ($CH_3$), 20.76 ($CH_3$), 20.74 ($CH_3$).

HRMS $[M+Na]^+$ m/z calcd. for $[C_{18}H_{27}O_{10}BrNa]^+$ 505.06798, found 505.06811.

*(2R,3S,4S,5R,6R)-2-(acetoxymethyl)-6-(3-bromopropoxy)tetrahydro-2H-pyran-3,4,5-triyl triacetate (3-bromopropyl)-2,3,4,6-tetra-O-acetyl-β-D-galactopyranoside (**14**)*

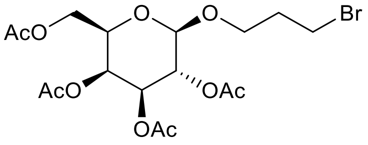

Procedure according to lit.[4], with slight modifications. To the solution of (2S,3R,4S,5S,6R)-6-(acetoxymethyl)tetrahydro-2H-pyran-2,3,4,5-tetrayl tetraacetate (2.16 g; 5.52 mmol) in dry DCM (10 ml) containing activated molecular sieves (cca 600 mg) 3-bromopropan-1-ol (97 %; 1 ml; 11.08 mmol) was added. Mixture was stirred for 1 h at room temperature, cooled to 0°C, and boron trifluoride diethyl etherate (3.48 ml; 28.67 mmol) was added in course of 10 min. The reaction mixture was stirred at room temperature for 23 hrs, diluted with DCM (200 ml), washed with H_2O (50 ml), $NaHCO_3$ sat. (40 ml), H_2O (40 ml), dried over Na_2SO_4 and concentrated. Crude product was purified on column of silica in toluene – EtOAc (20 to 25 %). Yield: 1.3 g, 50 %. Analytical data correspond to lit.[9]

General procedure 4 (for synthesis of **13**, **15** and **17**)

According to lit.[10], with slight modifications. To the well stirred solution of saccharide in DMSO, NaN_3 (6 eq) and tetrabutylammoium bromide (2 eq) were added. Reaction proceeded in darkness for 19-29h. Reaction mixture was diluted with DCM, washed with H_2O (2x), brine, dried over Na_2SO_4 and purified either on column of silica in toluene → EtOAc, or by flash chromatography in toluene → EtOAc.

(2R,3R,4S,5R,6R)-2-(acetoxymethyl)-6-(4-azidobutoxy)tetrahydro-2H-pyran-3,4,5-triyl triacetate (**13**)

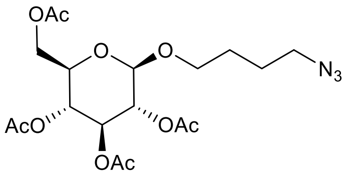

The general procedure 4 was followed, using 1.57 mmol of **12** in DMSO (3.7 ml). For the workup, the reaction mixture was diluted with DCM (150 ml), washed with H_2O (2 x 40 ml)), brine (40 ml), dried over Na_2SO_4 and purified on column of silica in toluene → EtOAc (10 to 25 %). Yield: 680 g, 97 % of white crystal-like compound.

δ_H (400 MHz, Chloroform-*d*) 5.20 (1 H, t, *J* 9.5, 2-H), 5.08 (1 H, dd, *J* 10.0, 9.4, 4-H), 4.98 (1 H, dd, *J* 9.6, 8.0, 3-H), 4.49 (1 H, d, *J* 8.0, 1-H), 4.29 – 4.08 (2 H, m, 6-$H_{a,b}$), 3.94 – 3.85 (1 H, m, OC$\boldsymbol{H_a}$$CH_2$), 3.68 (1 H, ddd, *J* 9.9, 4.7, 2.5, 5-H), 3.51 (1 H, dt, *J* 9.5, OC$\boldsymbol{H_b}$$CH_2$), 3.28 (2 H, tt, *J* 5.6, 2.5, $CH_2$$\boldsymbol{CH_2}$$N_3$), 2.08 (3 H, s, CH_3), 2.04 (3 H, s, CH_3), 2.02 (3 H, s, CH_3), 2.00 (3 H, s, CH_3), 1.65 (4 H, tdd, *J* 6.0, 3.6, 2.0, $0CH_2$$\boldsymbol{CH_2}$+$\boldsymbol{CH_2}$$CH_2N_3$).

δ_C (101 MHz, Chloroform-*d*) 170.81 (C=O), 170.43 (C=O), 169.54 (C=O), 169.42 (C=O), 100.87 (C-1), 72.94 (C-2), 71.94 (C-5), 71.41 (C-3), 69.36 (O***C***H_2CH_2), 68.55 (C-4), 62.07 (C-6), 51.19 (CH_2***C***H_2N_3), 26.73 (***C***$H_2CH_2N_3$), 25.59 (OCH_2***C***H_2), 20.88 (CH_3), 20.78 (CH_3), 20.76 (CH_3), 20.74 (CH_3).

HRMS $[M+Na]^+$ m/z calcd. for $[C_{18}H_{27}N_3NaO_{10}]^+$ 468.15887, found 468.15899.

(2R,3R,4S,5S,6S)-2-(acetoxymethyl)-6-(2-azidoethoxy)tetrahydro-2H-pyran-3,4,5-triyl triacetate (**17**)

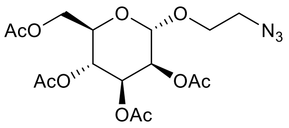

The general procedure 4 was followed, using 3.61 mmol of **16** in DMSO (9 ml). For the workup, the reaction mixture was diluted with DCM (250 ml), washed with H_2O (2 x 60 ml)), brine (60 ml), dried over Na_2SO_4 and purified by flash chromatography in PE → EtOAc (20→60 %). Yield: 1.5 g, 99 %
Analytical data corresponds to lit.[4]; HRMS $[M+Na]^+$ m/z calcd. for $[C_{16}H_{23}O_{10}N_3Na]^+$ 440.12756, found 440.12717.

(2R,3S,4S,5R,6R)-2-(acetoxymethyl)-6-(3-azidopropoxy)tetrahydro-2H-pyran-3,4,5-triyl)triacetate (**15**)

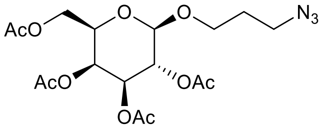

The general procedure 4 was followed, using 2.73 mmol of **14** in DMSO (8 ml). For the workup, the reaction mixture was diluted with DCM (200 ml), washed with H_2O (2 x 50 ml)), brine (50 ml), dried over Na_2SO_4 and purified by flash chromatography in PE – EtOAc (20$\rightarrow$60 %). Yield: 1.1 g, 90 %

Analytical data corresponds to lit.[11]

General procedure 5 (for synthesis of **Glc-C4-N_3**, **Gal-C3-N_3** and **Man-C2-N_3**)

According to lit.[12], with slight modifications. Dowex® 1x8 (in OH^- cycle, washed, dried) was placed into syringe equipped with sintered filter. The solution of acetylated saccharide in MeOH was added under argon, syringe was sealed and rotated for 16 hrs The solution was collected, the drained resin was washed with MeOH (12 x) and the washings together with collected solution were concentrated and co-distilled with toluene (2 x). Product was purified by flash chromatography.

(2R,3R,4S,5S,6R)-2-(4-azidobutoxy)-6-(hydroxymethyl)tetrahydro-2H-pyran-3,4,5-triol (**Glc-C4-N_3**)

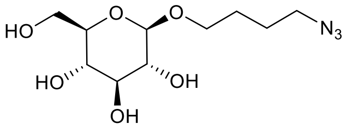

The general procedure 5 was followed using 1.48 mmol of **13**, 300 mg of Dowex® 1x8 and 10 ml of dry MeOH. The crude product was purified by flash chromatography in DCM - MeOH (5$\rightarrow$20 %) and lyophilized. Yield: 382, 93 %.

δ_H (500 MHz, Methanol-d_4) 1.65 – 1.75 (4 H, m, $OCH_2C\boldsymbol{H_2}C\boldsymbol{H_2}CH_2N_3$); 3.17 (1 H, dd, $J_{2,3}$ 9.2, $J_{2,1}$ 7.8, 2-H); 3.23 – 3.38 (5 H, m, 3,4,5-H, $OCH_2CH_2CH_2C\boldsymbol{H_2}N_3$); 3.58 (1 H, m, $OCH_a\boldsymbol{H_b}CH_2CH_2CH_2N_3$); 3.66 (1 H, dd, $J_{6b,6a}$ 11.9, $J_{6b,5}$ 5.4, 6_b-H); 3.87 (1 H, dd, $J_{6a,6b}$ 11.9, $J_{6a,5}$ 1.9, 6_a-H); 3.94 (1 H, m, $OC\boldsymbol{H_a}H_bCH_2CH_2CH_2N_3$); 4.25 (1 H, d, $J_{1,2}$ 7.8, 1-H).

δ_C (126 MHz, Methanol-d_4) 26.71, 27.93 ($OCH_2\boldsymbol{C}H_2\boldsymbol{C}H_2CH_2N_3$); 52.29 ($OCH_2CH_2CH_2\boldsymbol{C}H_2N_3$); 62.75 ($CH_2$-6); 70.06 ($O\boldsymbol{C}H_2CH_2CH_2CH_2N_3$); 71.63 (4-C); 75.09 (2-C); 77.93 (5-C); 78.10 (3-C); 104.33 (1-C).

HRMS $[M+Na]^+$ m/z calcd. for $[C_{10}H_{19}O_6N_3Na]^+$ 300.11661, found 300.11660.

(2S,3S,4S,5S,6R)-2-(2-azidoethoxy)-6-(hydroxymethyl)tetrahydro-2H-pyran-3,4,5-triol (***Man-C2-N$_3$***)

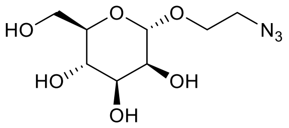

The general procedure 5 was followed, using 3.42 mmol of **17**, 850 mg of Dowex® 1x8 and 15 ml of MeOH (HPLC grade). The crude product was purified by flash chromatography on C18 phase in H_2O - ACN (10 %) and lyophilized. Yield: 714 mg, 84 %. Analytical data correspond to lit.[4]

δ_H (500 MHz, Methanol-d_4) 3.38 – 3.45 (2 H, m, CH_2N_3); 3.57 (1 H, ddd, $J_{5,4}$ 9.8, $J_{5,6}$ 5.8, 2.2, 5-H); 3.60 – 3.65 (2 H, m, 4-H, $CH_a\boldsymbol{H_b}O$); 3.71 (1 H, dd, $J_{6b,6a}$ 11.8, $J_{6b,5}$ 5.8, 6_b-H); 3.73 (1 H, dd, $J_{3,4}$ 9.0, $J_{3,2}$ 3.4, 3-H); 3.84 (1 H, dd, $J_{2,3}$ 3.4, $J_{2,1}$ 1.8, 2-H); 3.85 (1 H, dd, $J_{6a,6b}$ 11.8, $J_{6a,5}$ 2.2, 6_a-H); 3.92 (1 H, m , $C\boldsymbol{H_a}H_bO$); 4.81 (1 H, d, $J_{1,2}$ 1.8, 1-H).

δ_C (126 MHz, Methanol-d_4) 51.70 (CH_2N_3); 62.90 (6-C); 67.70 (CH_2O); 68.48 (4-C); 72.03 (2-C); 72.43 (3-C); 74.89 (5-C); 101.77 (1-C).

HRMS $[M+H]^+$ m/z calcd. for $[C_8H_{14}O_6N_3]^+$ 248.08881, found 248.08905.

(2R,3R,4S,5R,6R)-2-(3-azidopropoxy)-6-(hydroxymethyl)tetrahydro-2H-pyran-3,4,5-triol (***Gal-C3-N$_3$***)

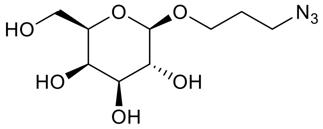

The general procedure 5 was followed, using 2.39 mmol of **15**, 550 mg of Dowex® 1x8 and 10 ml of MeOH (HPLC grade). The crude product was purified by flash chromatography on reverse phase in H_2O - ACN (5→30 %) and lyophilized. Yield: 627 mg, 87 %. Analytical data in accordance with lit.[13]

δ_H (500 MHz, Methanol-d_4) 1.87 (2 H, m, $OCH_2C\boldsymbol{H_2}CH_2N_3$); 3.43 – 3.5 (5 H, m, 2,3,5-H, $OCH_2CH_2C\boldsymbol{H_2}N_3$); 3.64 (1 H, dt, J_{gem} 10.0, J_{vic} 6.1, $OCH_a\boldsymbol{H_b}CH_2CH_2N_3$); 3.72 (1 H, dd, $J_{6b,6a}$ 11.3, $J_{6b,5}$ 5.6, H-6b); 3.75 (1 H, dd, $J_{6a,6b}$ 11.3, $J_{6a,5}$ 6.8, 6_a-H); 3.83 (1 H, dd, $J_{4,3}$ 3.3, $J_{4,5}$ 1.1, 4-H); 3.97 (1 H, dt, J_{gem} 10.0, J_{vic} 6.0, $OC\boldsymbol{H_a}H_bCH_2CH_2N_3$); 4.21 (1 H, d, $J_{1,2}$ 7.5, 1-H).

δ_C (126 MHz, Methanol-d_4) 30.28 ($OCH_2\boldsymbol{C}H_2CH_2N_3$); 49.38 ($OCH_2CH_2\boldsymbol{C}H_2N_3$); 62.44 (6-C); 67.50 ($O\boldsymbol{C}H_2CH_2CH_2N_3$); 70.25 (4-C); 72.51 (2-C); 74.94 (3-C); 76.64 (5-C); 105.07 (1-C).

Prepration of model peptides **Pep14** and **Pep15**:

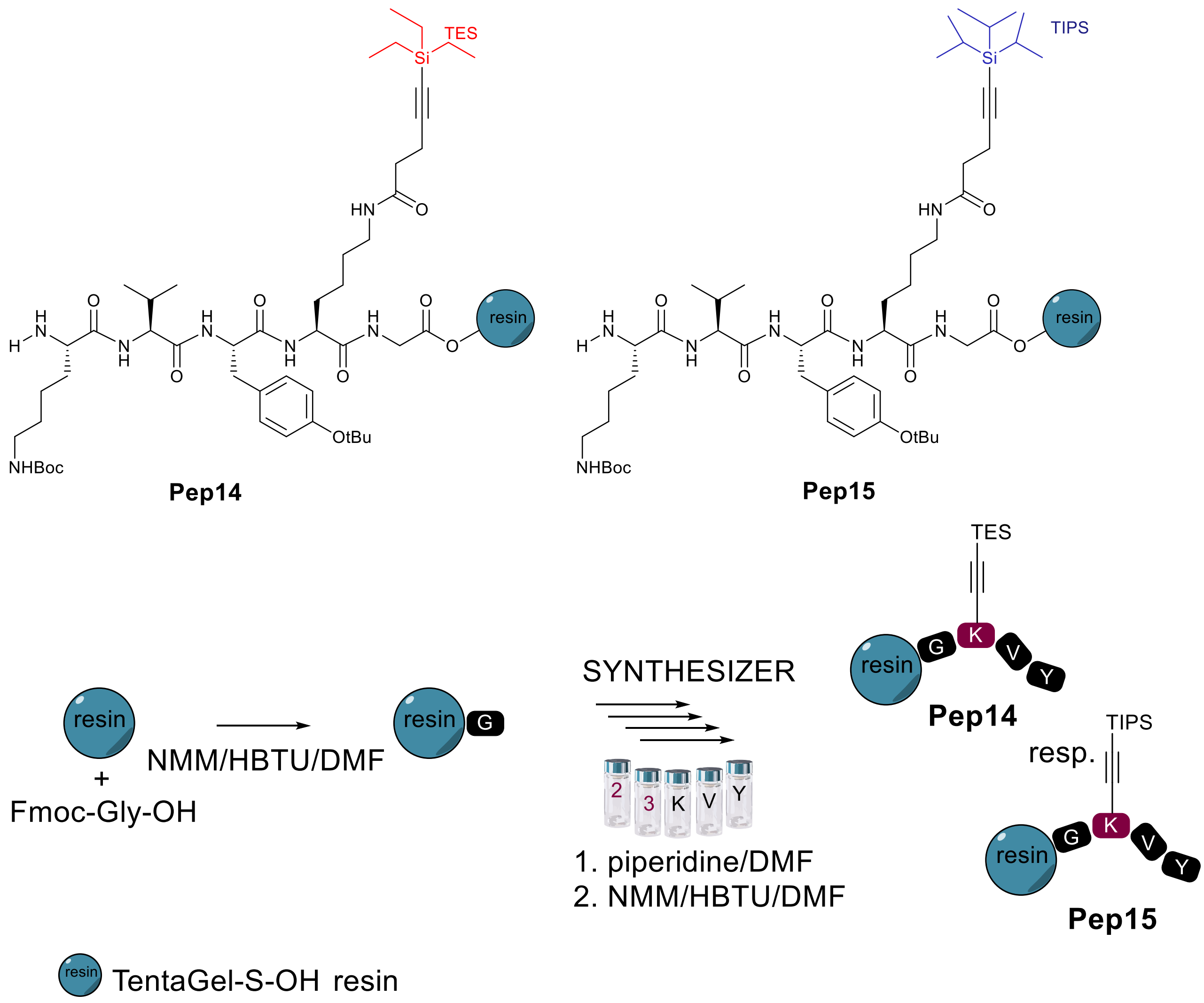

Scheme S3: Synthesis of model peptides **Pep14** and **Pep15**

Resin loading:

TentaGel-S-OH resin (269 mg, theor. 0.065 mmol) was soaked in dry DMF (2 ml) for 1.5 h and drained. In separate flask, Fmoc-Gly-OH (594.6 mg; 2 mmol) was taken into dry DMF (1.5 ml), dry DCM (6 ml) was added, the solution was cooled to 0 ºC, DIC (155 µl; 1 mmol) was added. The reaction mixture was stirred at 0 ºC for 45 min. DCM was removed, the residue was dissolved in dry DMF, and the resulting solution was added to the drained resin. DMAP (2.44 mg; 0.02 mmol) was added, the syringe was flushed with argon and rotated on rotary shaker for 2 hrs. Resin was drained, washed with DMF (5x), DCM (5x) and dried. Yield: 257 mg of dried loaded resin. Loading by Fmoc estimation: 0.188 mmol/g; AA analysis: 0.137 mmol/g.

Fmoc estimation method:

Done according lit.[14] Dry loaded resin (approx. 5 mg, weighted precisely) was treated by DBU (2 % in DMF; 2 ml) for 40 min. The solution was collected, diluted to 10 ml with ACN, resulting solution was diluted for the second time (0.8 ml of solution to 10 ml by ACN), and its' optical density measured on

UV spectrometer in optical cell (1 cm) at 304 nm. Blanc control sample was used to determine optical background. The resin load was calculated as

$$loading\left[\frac{mmol}{g}\right] = A^{304} * \frac{16.4}{m_{resin}[mg]}$$

Automated SPPS

Automated peptide synthesis was performed on PS3 peptide synthesizer, *Protein Technologies, Inc.*. Synthesizer was loaded with glycine substituted resin (128 mg; 0.024 mmol for **Pep14**, 157 mg; 0.03 mmol for **Pep15**). Peptide was synthesized under standard automated Fmoc protocols (see below), using either 4 equivalents of each commercial amino acid and 4 equivalents of HBTU as coupling agent, or 2 equivalents of modified amino acids **2** or **3** and 2 equivalents of HBTU, in both cases activated by NMM (0.4 M in DMF). The remaining Fmoc-group was removed after the last coupling step using the automated protocol, the deprotection solution was piperidine : DMF (1 : 4).

The following Fmoc protected amino acids were utilized:
Step 1: Fmoc-L-Lys(pentynoyl-TES)-OH (**2**) for **Pep14**, Fmoc-L-Lys(pentynoyl-TIPS)-OH (**3**) for **Pep15**
Step 2: Fmoc-L-Tyr(tBu)-OH
Step 3: Fmoc-L-Val-OH
Step 4: Fmoc-L-Lys(Boc)-OH
Step 5: Deprotection – removal of Fmoc group

The resin was transferred from synthesizer reaction vessel to syringe (10 ml) equipped with sintered filter, washed with DCM (6x), drained, and dried. Yield: 143 mg (**Pep14**), resp. 166 mg (**Pep15**) of loaded resin. Small sample of resin (approx. 2 mg) was treated with NaOH following the general procedure bellow and the progress of the synthesis was verified by LC-MS analysis.

Standard protocols for SP3 Peptide Synthesizer:

Deprotection solution: piperidine (20 % in DMF)
Activation solution: NMM (0.4M in DMF)

Coupling step:

STEP	FUNCTION	DURATION	REPEATED
**1**	Wash with DMF	30 s (10 min for the first coupling)	3 (2 for the first coupling)
**2**	depr. of N-terminus by deprotection solution	5 min.	2
**3**	Wash with DMF	30 s	6
**4**	Amino acid + base dissolved in the activation solution	30 s	1
**5**	amino acid coupling	30 min	1
**6**	Wash with DMF	30 s	3

Deprotection step:

STEP	FUNCTION	DURATION	REPEATED
**1**	Wash with DMF	30 s	3
**2**	depr. of N-terminus by deprotection solution	5 min.	2
**3**	Wash with DMF	30 s	6

General procedure 6: Cleavage-off of TentaGel-S-OH resin with NaOH (for analytical samples)

To the loaded TentaGel-S-OH resin (approx. 1-2 mg), the solution of NaOH (0.1M in H_2O; 50 µl) was added, reaction mixture was shaken for 90 min., neutralized by HCl (0.2M in H_2O) and the liquids were collected. Drained resin was washed with warm ACN (2 x 20 µl), ACN washes were mixed with previously collected liquids and analyzed by LC-MS.

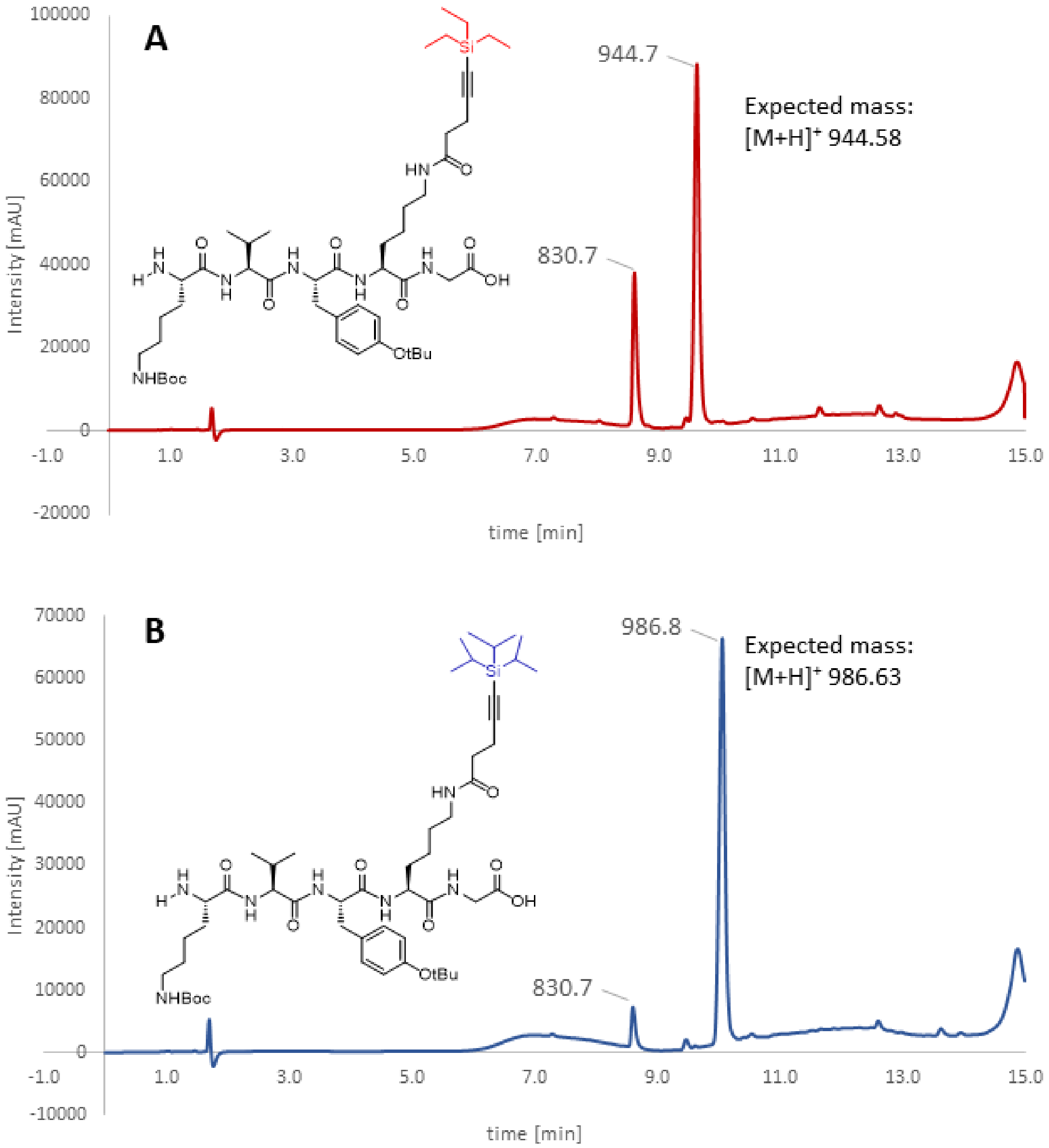

*Figure S1: LC-MS analyses of comtounds **Pep14** (**A**) and **Pep15** (**B**), at 270 nm. Molecular mass 830.7 corresponds to peptide with free alkyne. Note: silyl protective groups are not completely stable under cleavage conditions (0.1M NaOH for 1.5 hr)*

Prepration of model peptide **Pep16**:

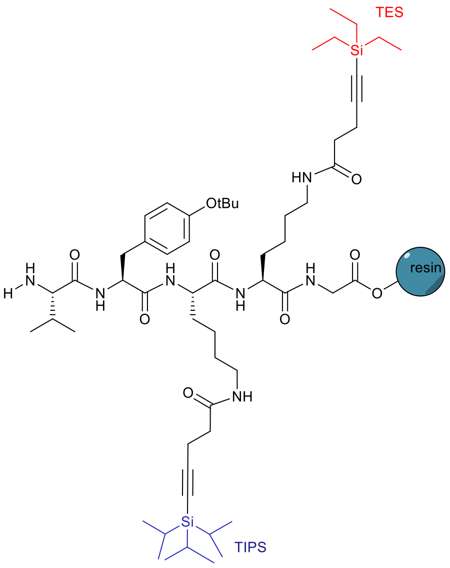

Resin loading:

TentaGel-S-OH resin (517 mg, theor. 0.124 mmol) was soaked in dry DMF (3 ml) for 1 hr and drained. In separate flask, Fmoc-Gly-OH (594.6 mg; 2 mmol) was taken into dry DMF (1 ml), dry DCM (8 ml) was added, the solution was cooled to 0 ºC, DIC (155 µl; 1 mmol) was added. The reaction mixture was stirred for at 0 ºC for 1 hr. DCM was removed, the residue was dissolved in dry DMF (4 ml), and 2/3 of the resulting solution was added to the drained resin. DMAP (1.6 mg; 0.013 mmol) was added, the syringe was flushed with argon and rotated on the rotary shaker for 1 hr. Resin was drained, the remaining 1/3 of anhydride solution together with DMAP (0.08 mg; 0.07 mmol) were transfered to the drained resin, the syringe was flushed with argon and rotated for 1 hr. The resin was drained, washed with DMF (5x), DCM (5x) and dried. Yield: 541.2 mg of dried loaded resin. Loading by Fmoc estimation: 0.139 mmol/g; AA analysis: 0.184 mmol/g.

SPPS

Solid phase peptide synthesis was performed "by hand" following the lit.[15].

Fmoc-Glycine substituted TentaGel-S-OH resin (259 mg; 0.033 mmol) was swelled in DCM (15 min), and washed with DMF (1.5 ml; 30 s).

Fmoc removal:
Piperidine (20 % v/v in DMF; 1.5 ml) was added, resin was shaken for 2 min and drained.
Piperidine (20 % v/v in DMF; 1.5 ml) was added, resin was shaken for 12 min and drained.
Resin washed with DMF (4 x 2 ml)

Coupling step:
Fmoc-L-Lys(pentynoyl-TES)-OH (**2**) (37.2 mg; 0.066 mmol) and HBTU (25 mg; 0.066 mmol) were dissolved in DMF (1.5 ml), and added to the drained resin.
NMM (35 ml; 0.32 mmol) was added.
The resin was mixed with N_2 stream for 30 min.
The resin was drained; analytical sample was washed coupling conversion verified by ninhydrin test.
The resin was washed with DMF (6 x 2 ml) and DCM (4 x 2 ml).

Deprotection-coupling cycle was repeated using:
2. coupling: Fmoc-L-Lys(pentynoyl-TIPS)-OH (**3**) (39.9 mg; 0.066 mmol)
3. coupling: Fmoc-L-Tyr(tBu)-OH (60.66 mg; 0.066 mmol)
4. coupling: Fmoc-L-Val-OH (44.8 mg; 0.132 mmol)

Last Fmoc removal:
Piperidine (20 % v/v in DMF; 1.5 ml) was added, resin was shaken for 2 min and drained.
Piperidine (20 % v/v in DMF; 1.5 ml) was added, resin was shaken for 12 min and drained.
Resin washed with DMF (6 x 2 ml), DCM (4 x 2 ml), dried 2 h under vacuum.

Yield: 335 mg of substituted resin. Small sample of resin (approx. 2 mg) was treated with NaOH following the general procedure 6 and the progress of the synthesis was verified by LC-MS analysis.

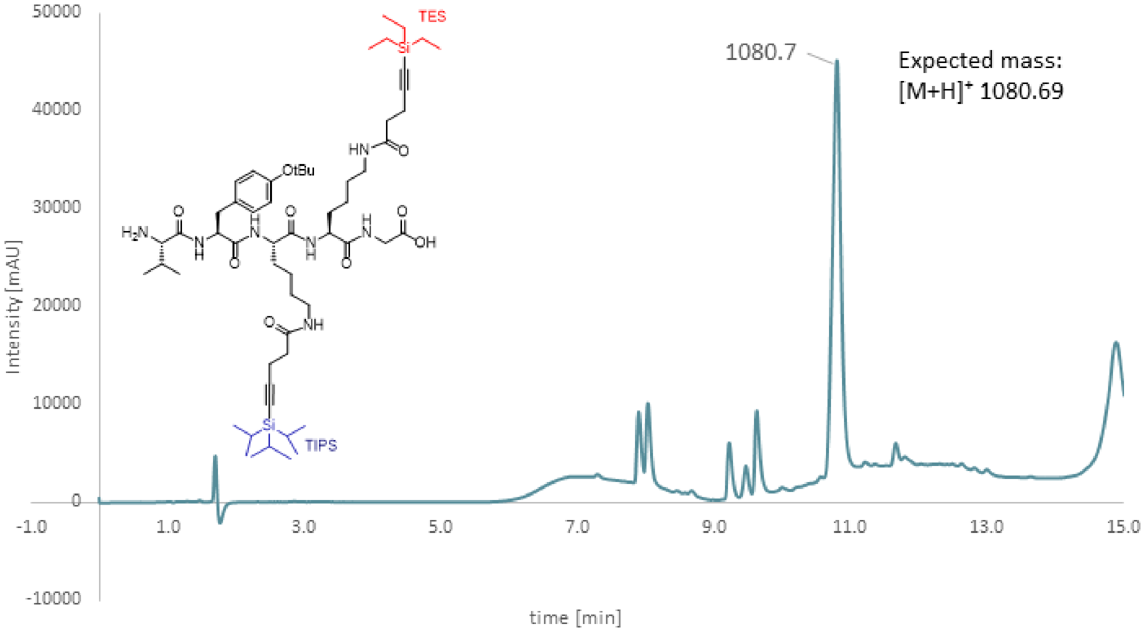

*Figure S2: LC-MS analysis of compound **Pep16** at 270 nm.*

Synthesis of model modified peptide **Pep1**

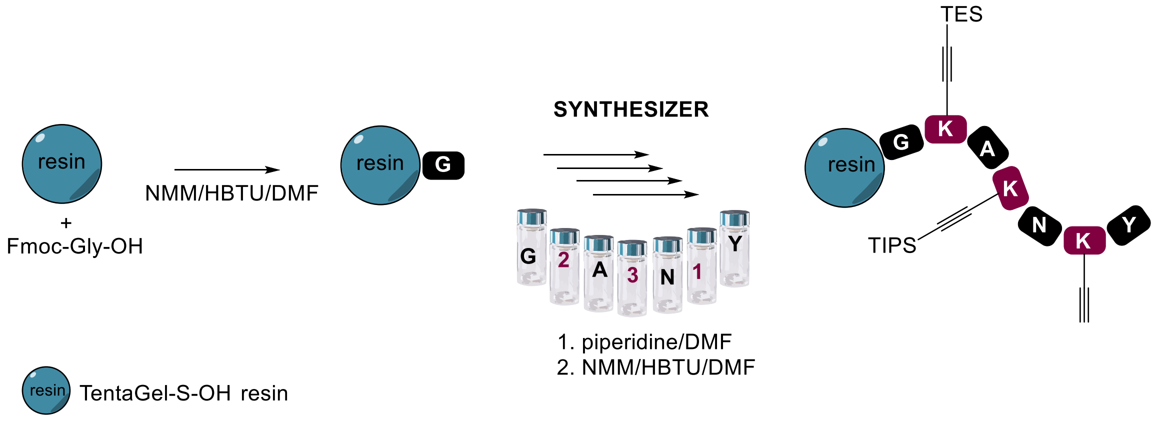

Scheme S4: Synthesis of model peptide **Pep1**

Peptide **Pep1** was synthesized on PS3 peptide synthesizer using L-glycine-loaded resin, standard Fmoc chemistry and 4 (resp. 2) equivalents of animo acid for coupling step. For detailed procedure see synthesis of **Pep14**.

The following Fmoc protected amino acids were utilized:
Step 1: Fmoc-L-Lys(pentynoyl-TES)-OH (**2**)
Step 2: Fmoc-L-Ala-OH *H_2O
Step 3: Fmoc-L-Lys(pentynoyl-TIPS)-OH (**3**)
Step 4: Fmoc-L-Asn(Trt)-OH
Step 5: Fmoc-L-Lys(pentynoyl)-OH (**1**)
Step 6: Fmoc-L-Tyr(tBu)-OH
Step 7: Deprotection – removal of Fmoc group

The resin was transferred from synthesizer reaction vessel to syringe (10 ml) equipped with sintered filter, washed with DCM (4x), drained, and dried. Yield: 327 mg of loaded resin. Small sample of resin (approx. 2 mg) was treated with NaOH following the general procedure 6, using significantly shorter cleavage time (10 min), and analyzed by LC-MS (*Agilent*).

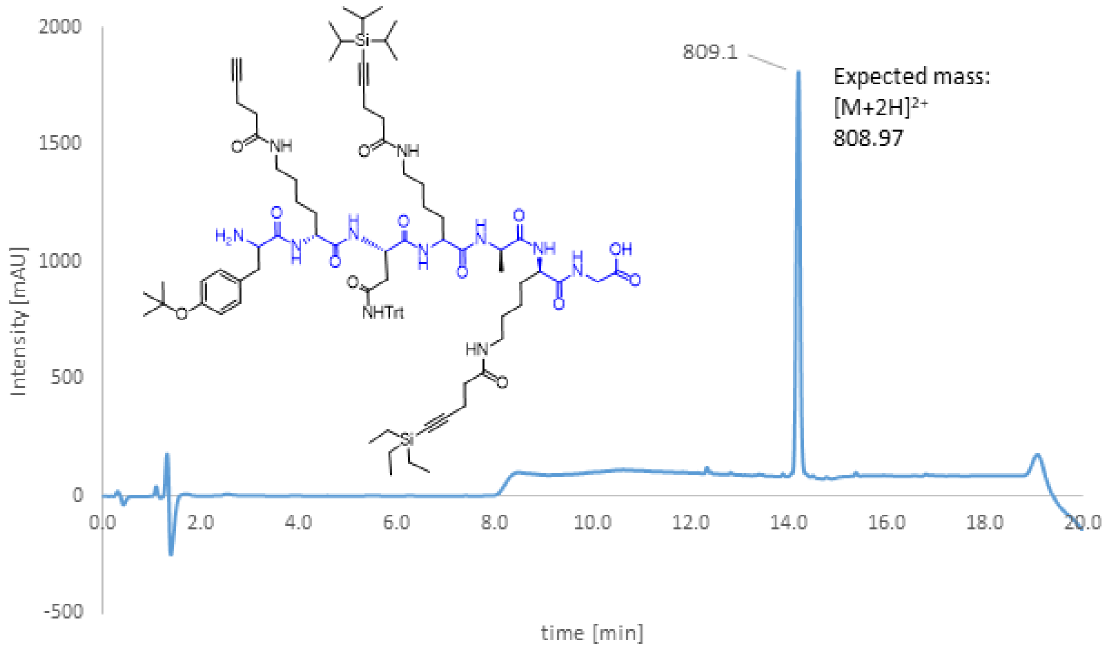

*Figure S3: LC-MS analysis of compound **Pep1** at 214 nm.*

Synthesis of glycosylated peptide **Pep10**:

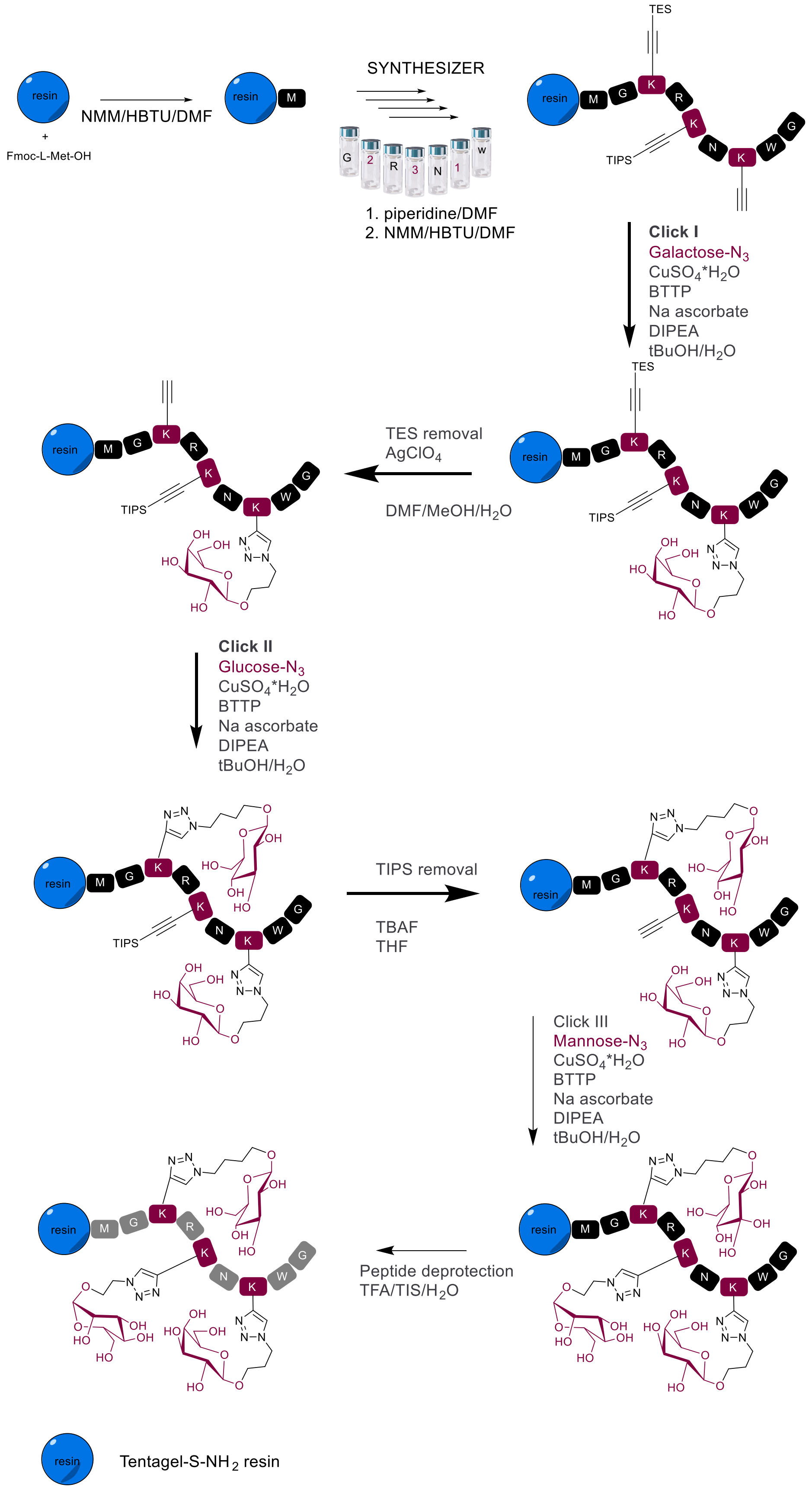

Scheme S5: Synthesis of tripple-glycosylated peptide **Pep10**

Resin loading:

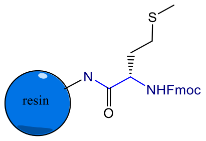

TentaGel-S-NH_2 resin (514 mg, theor. 0.134 mmol) was soaked in DMF (5ml) for 1 h and drained. The solution of Fmoc-L-Met-OH (201.2 mg; 0.53 mmol) and HBTU (203.3 mg; 0.54 mmol) in NMM (0.4 M in DMF, 3 ml) was added, syringe was flushed with Ar and rotated for 75 min. Resin was drained, washed with DMF (6x), DCM (6x) and dried. Yield: 536 mg of dried loaded resin. Loading by Fmoc estimation: 0.197 mmol/g; AA analysis: 0.164 mmol/g. For Fmoc estimation method see synthesis of **Pep14**.

Automated SPPS

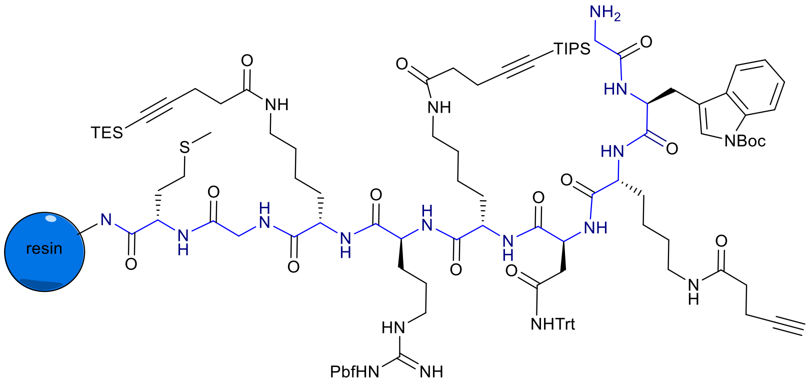

Automated peptide synthesis was performed on PS3 peptide synthesizer, Protein Technologies, Inc. Synthesizer was loaded with methionine substituted resin (250 mg; 0.049 mmol). Resin was capped with acetic anhydride under standard automated protocol, than peptide was synthesized under standard automated Fmoc protocols, using either 4 equivalents of each commercial amino acid and 4 equivalents of HBTU as coupling agent, or 2 equivalents of modified amino acid **2** or **3** and 2 equivalents of HBTU. The remaining Fmoc-group was removed after the last coupling step by piperidine : DMF (1 : 4).

The following Fmoc protected amino acids were utilized:
Step 1: Capping with acetic anhydride
Step 1: Fmoc-Gly-OH
Step 2: Fmoc-L-Lys(pentynoyl-TES)-OH (**2**)
Step 3: Fmoc-L-Arg(Pbf)-OH
Step 4: Fmoc-L-Lys(pentynoyl-TIPS)-OH (**3**)
Step 5: Fmoc-L-Asn(Trt)-OH
Step 6: Fmoc-L-Lys(pentynoyl)-OH (**1**)
Step 7: Fmoc-L-Trp(Boc)-OH
Step 8: Fmoc-Gly-OH
Step 9: deprotection – removal of Fmoc group

The resin was transferred from synthesizer reaction vessel to syringe (10 ml) equipped with sintered filter, washed with DCM (6x), drained, and dried. Yield: 324 mg of loaded resin. Small sample of resin (approx. 2 mg) was treated with CNBr following the general procedure and the progress of the synthesis was verified by LC-MS analysis.

Standard protocols for SP3 Peptide Synthesizer:

For deprotection and coupling protocol details see synthesis of **Pep14**.

Capping step:

Capping solution: acetic anhydride (2 ml)

STEP	FUNCTION	DURATION	REPEATED
**1**	Wash with DMF	30 s	6
**2**	Mixing of capping solution in vial	30 s	1
**3**	Capping	20 min.	1
**6**	Wash with DMF	30 s	3

General procedure 7: Cleavage-off of TentaGel-S-NH_2 resin with CNBr (for analytical samples)

Small amount of the analyzed resin (approx. 2 mg) was soaked in water for 15 min and drained. H_2O (20 μl) and CNBr (0.5 M in 0.2M HCl; 20 μl) were added, the vial was flushed with argon, sealed and MW irradiated (900W 2 x 3 s with 27 s break). The resin was drained, washed with warm ACN (approx. 40-50°C; 2x), ACN fractions were collected and measured on LC-MS.

Modification of resin-bound protected peptide **Pep4**

Click I

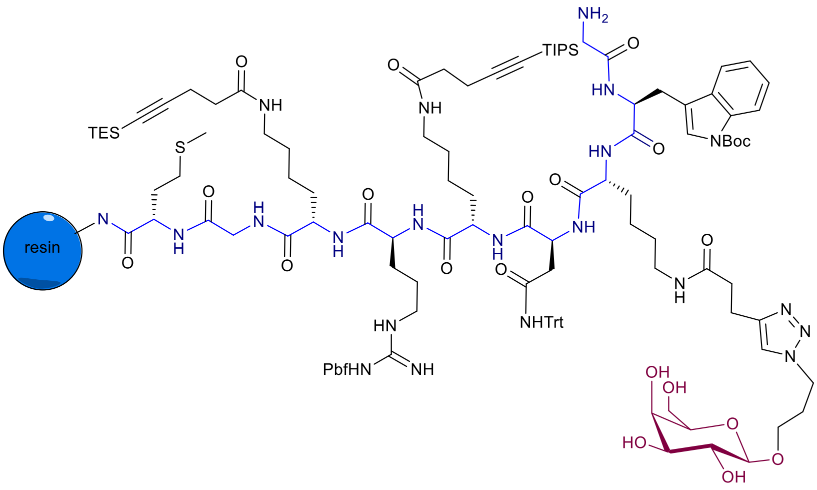

Dry resin (71 mg; 0.014 mmol) was soaked in t-BuOH : H_2O (2 : 3) for 50 min. and drained. The solution of 3-azidopropyl β-D-galactopyranoside **Gal-C3-N_3** (0.4 M in H_2O; 58.2 μl; 0.023 mmol) was added and syringe was thoroughly agitated. The coupling solution was prepared in separate vial: to the solution of $CuSO_4$ *$5H_2O$ (0.1M in H_2O; 29,1 μl) the solution of BTTP (0.05M in t-BuOH; 116.4 μl) was added. Solution of sodium ascorbate (0.05M in H_2O; 116.4 μl) was added and the resulting colorless solution was transferred to the resin-azide mixture. DIPEA (4.46 ml; 0.026 mmol) was added, the vial was flushed with argon, sealed and gently rotated for 4h. The resin was drained, small sample treated with CNBr following the general procedure and the progress of the synthesis was verified by LC-MS analysis. The click procedure was repeated again, using the same amount of reagent and catalyst. The resin was washed with DMSO (10x), H_2O (3x), DMSO (3x), DCM (4x) and dried.

TES removal:

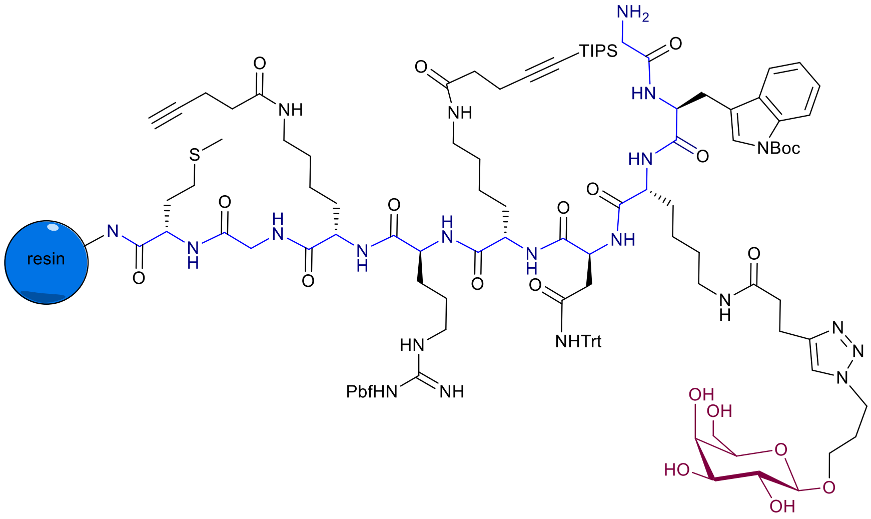

Dried resin was soaked in DMF : MeOH : H_2O (60 : 32 : 8) for 25 min. and drained. The solution of $AgClO_4$ (0.2M in DMF : MeOH : H_2O (60 : 32 : 8); 325 μl) was added under argon, the vial was sealed and rotated slowly for 2h with exclusion of light. Resin was drained, washed with DMF (5x), H_2O (2x), KCN (0.5 M in H_2O; 4x), H_2O (2x), DMSO (2x), DCM (3x) and dried.

Click II

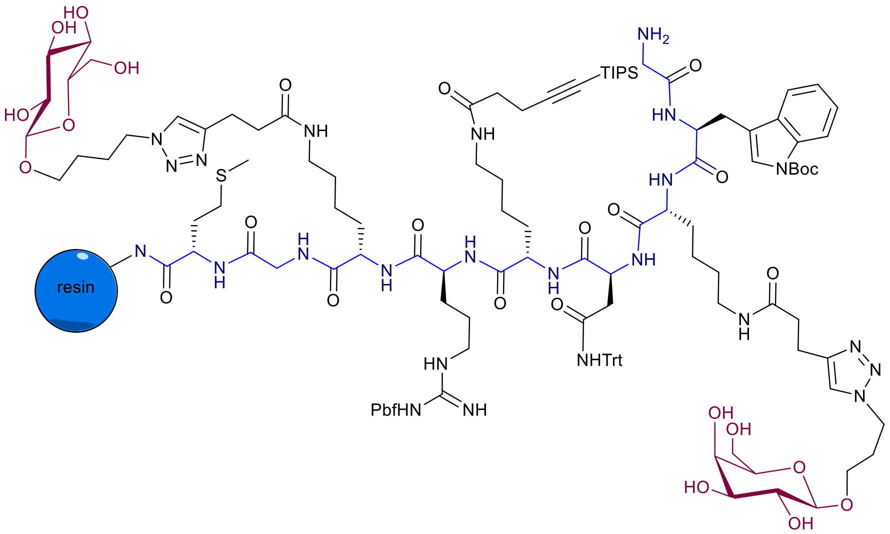

Dry resin (48 mg; 0.0079 mmol) was soaked in t-BuOH : H_2O (2 : 3) for 50 min. and drained. The solution of 4-azidobutyl β-D-glucopyranoside **Glc-C4-N$_3$** (0.4 M in H_2O; 39.4 μl; 0.016 mmol) was added and syringe was thoroughly agitated. The coupling solution was prepared in separate vial: to the solution of $CuSO_4$ *5H_2O (0.1M in H_2O; 18.7 μl) the solution of BTTP (0.05M in t-BuOH; 78.7 μl) was added. Solution of sodium ascorbate (0.05M in H_2O; 78.7 μl) was added and the resulting colorless solution was transferred to the resin-azide mixture. DIPEA (3.02 ml; 0.017 mmol) was added, the vial was flushed with argon, sealed and gently rotated for 4h. The resin was drained, small sample treated with CNBr following the general procedure and the progress of the synthesis was verified by LC-MS analysis. The resin was washed with DMSO (10x), H_2O (3x), DMSO (3x), DCM (4x) and dried.

TIPS removal:

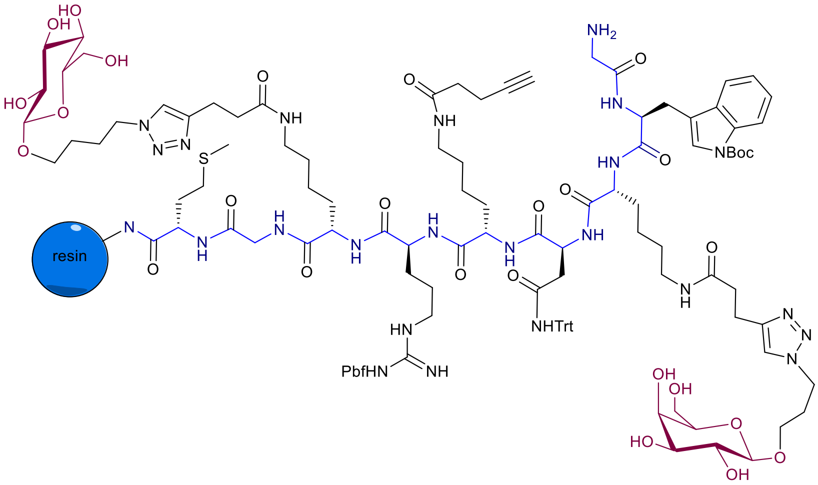

To the dry resin (48 mg; 0.0079 mmol) TBAF (1 M in THF; 80 μl) was added. The vial was flushed with argon, sealed and shaken for 3h. The resin was drained, washed with DMSO (2x), H_2O (2x), DMF (2x) DCM (2x) and dried. Small sample was treated by CNBr following the general procedure and the progress of the synthesis was verified by LC-MS analysis.

Click III

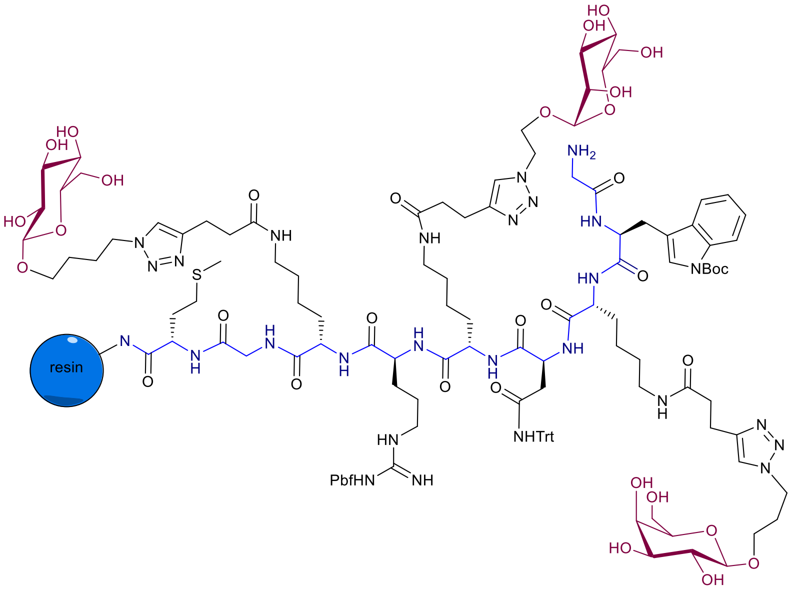

Dry resin (34 mg; 0.0056 mmol) was soaked in t-BuOH : H_2O (2 : 3) for 45 min. and drained. The solution of 4-azidobutyl α-D-mannopyranoside **Man-C2-N$_3$** (0.4 M in H_2O; 27.9 μl; 0.011 mmol) was added and the mixture was thoroughly agitated. The coupling solution was prepared in separate vial: to the solution of $CuSO_4$ *5H_2O (0.1M in H_2O; 13.9 μl) the solution of BTTP (0.05M in t-BuOH; 55.8 μl) was added. Solution of sodium ascorbate (0.05M in H_2O; 55.8 μl) was added and the resulting colorless solution was transferred to the resin-azide mixture. DIPEA (2.14 ml; 0.012 mmol) was added, the vial was flushed with argon, sealed and gently rotated for 16h. The resin was drained, small sample treated with CNBr following the general procedure and the progress of the synthesis was verified by LC-MS analysis. The resin was washed with DMSO (10x), H_2O (3x), DMSO (3x), DCM (4x) and dried.

Deprotection of the modified peptide:

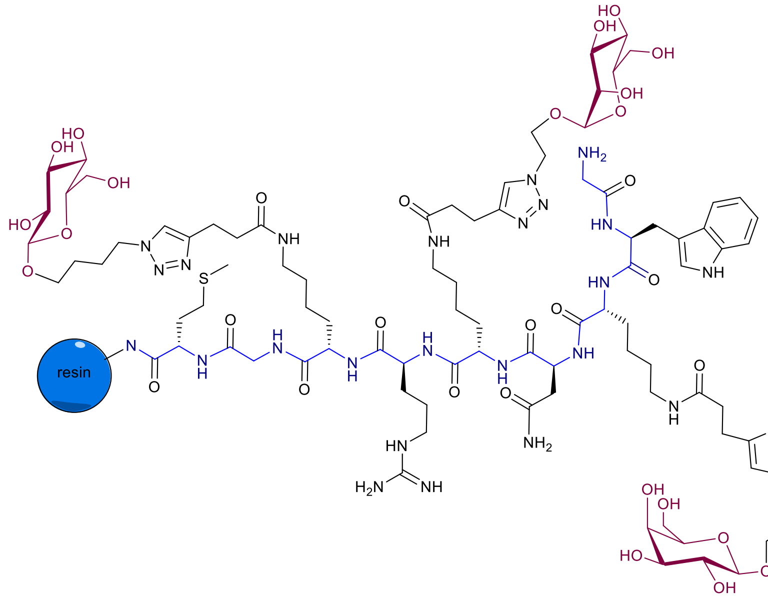

To the dry resin, the mixture of TFA : H_2O : TIS (95 : 2.5 : 2.5; 0.4 ml) was added. The syringe was gently rotated for 1 hr, TFA cocktail was replaced by a fresh batch (0.4 ml), reaction mixture was rotated for additional 1 hr, drained, washed with DMSO (5x), DCM (5x), DMSO (3x), H_2O (3x). NaOH (1.1M on H_2O; 0.3 ml) was added and reaction mixture was rotated for 1 hr. The resin was drained, washed with H_2O (5x), DMSO (3x), DCM (5x) and dried. Small sample was treated with CNBr following the general procedure, and resulting solution analyzed by LC-MS analysis.

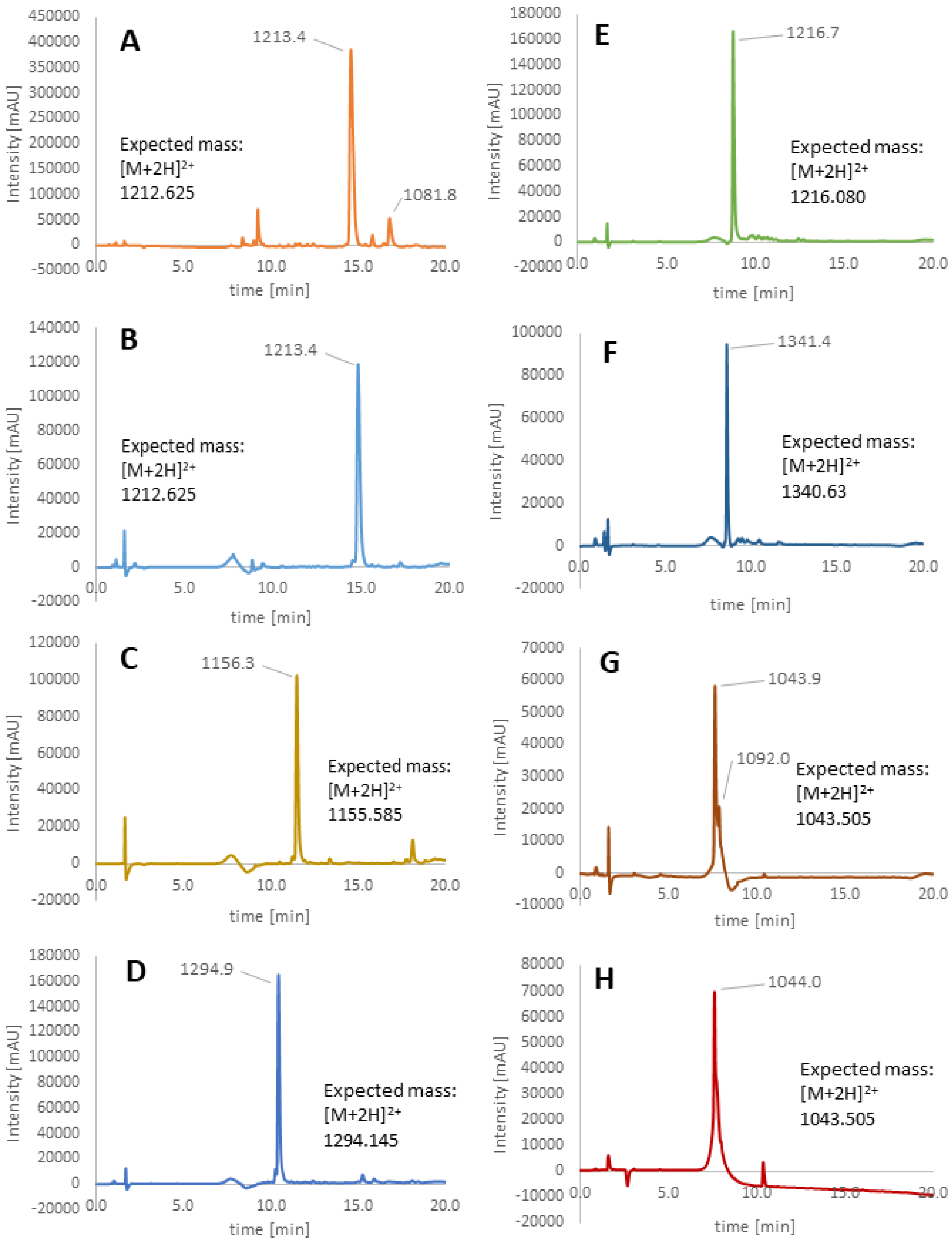

*Figure S4: Synthesis of **Pep10**, LC-MS analyses of peptide modifications. **A**: Click I (galactose), after first CuAAC round - detected mass 1081.8 corresponds to remaining non-modified peptide; **B**: Click I (galactose), after repeated CuAAC reaction; **C**: TES removal; **D**: Click II (glucose); **E**: TIPS removal; **F**: Click III (mannose); **G**: peptide deprotection in TFA cleavage coctail - detected mass 1092.0 corresponds to TFA acetate; **H**: deprotected peptide after hydrolysis of TFA acetate by NaOH. UV absorption measured at 266 nm.*

Synthesis of **Pep11**

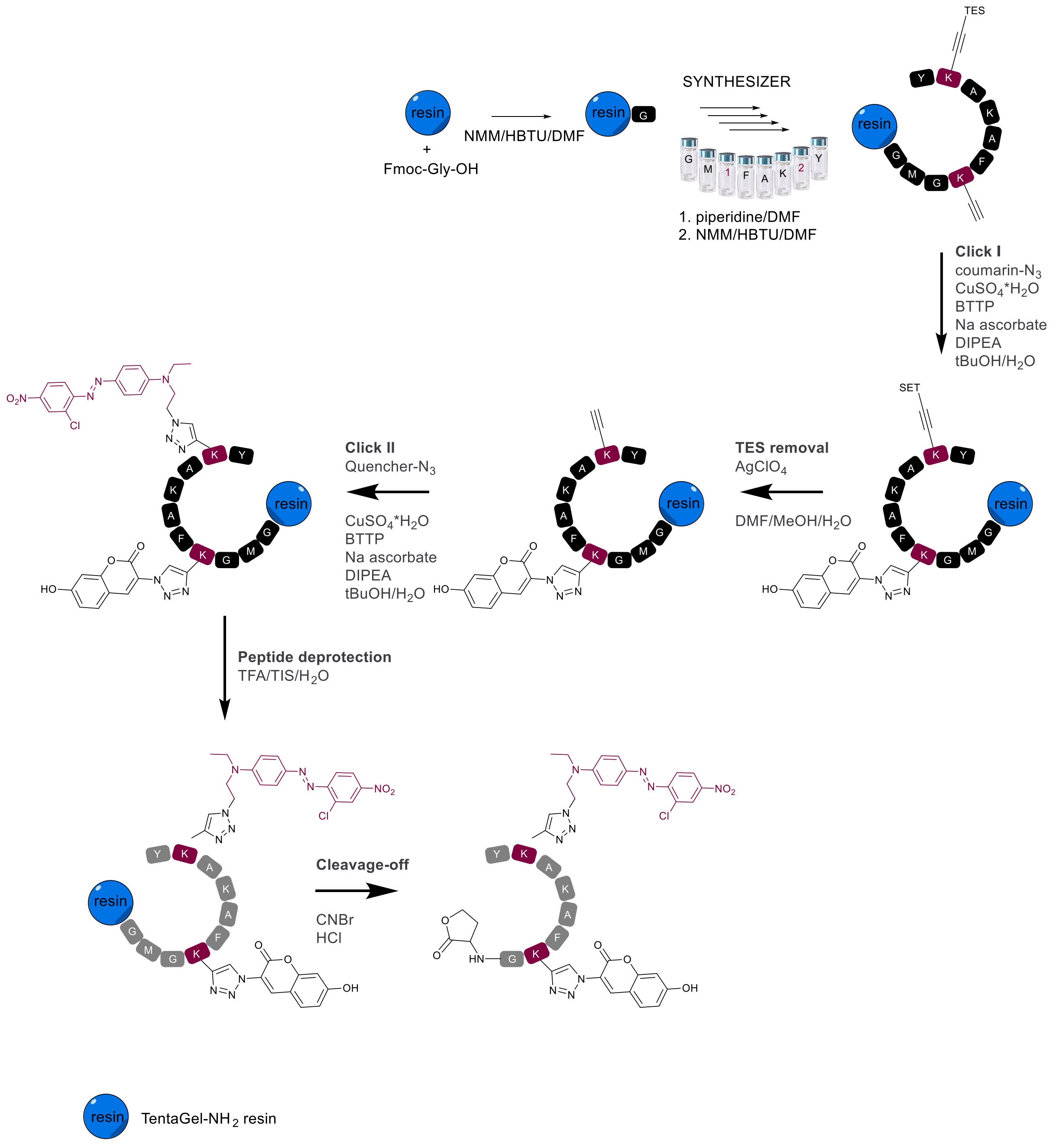

Scheme S6: Synthesis of doubble modified fluorogenic peptide AK-280

Resin loading:

TentaGel-S-NH$_2$ resin (500 mg, theor. 0.13 mmol) was soaked in DMF (5ml) for 40 min and drained. The solution of Fmoc-Gly-OH (154.6 mg; 0.52 mmol) and HBTU (197.2 mg; 0.52 mmol) in NMM (0.4 M in DMF, 3 ml) was added, syringe was flushed with Ar and rotated for 75 min. Resin was drained, washed with DMF (6x), DCM (6x) and dried. Yield: 571 mg of dried loaded resin. Loading by Fmoc estimation (for details see synthesis of **Pep14**): 0.184 mmol/g; AA analysis: 0.176 mmol/g.

Automated SPPS

Automated peptide synthesis was performed on PS3 peptide synthesizer, *Protein Technologies, Inc.*. Synthesizer was loaded with glycine-substituted resin (275 mg; 0.046 mmol). Resin was capped with acetic anhydride under standard automated protocol, than peptide was synthesized under standard automated Fmoc protocols, using either 4 equivalents of each commercial amino acid and 4 equivalents of HBTU as coupling agent, or 2 equivalents of modified amino acid **1** or **2** and 2 equivalents of HBTU. The remaining Fmoc-group was removed after the last coupling step, the deprotection solution was piperidine : DMF (1 : 4). For the standard protocol details see synthesis of **Pep14** (coupling and deprotection and capping step)

The following Fmoc protected amino acids were utilized:
Step 1: Fmoc-L-Met-OH
Step 2: Fmoc-Gly-OH
Step 3: Fmoc-L-Lys(pentynoyl)-OH (**1**)
Step 4: Fmoc-L-Phe-OH,
Step 5: Fmoc-L-Ala-OH * H_2O
Step 6: Fmoc-L-Lys(Boc)-OH
Step 7: Fmoc-L-Ala-OH * H_2O
Step 8: Fmoc-L-Lys(pentynoyl-TES)-OH (**2**)
Step 9: Fmoc-L-Tyr(tBu)-OH

The resin was transferred from synthesizer reaction vessel to syringe (10 ml) equipped with sintered filter, washed with DCM (6x), drained, and dried. Yield: 362 mg of loaded resin. Small sample of resin (approx. 2 mg) was treated with CNBr following the general procedure and the progress of the synthesis was verified by LC-MS analysis.

Modification of resin-bound protected peptide

Click I – 3-azido-7-hydroxycoumarin

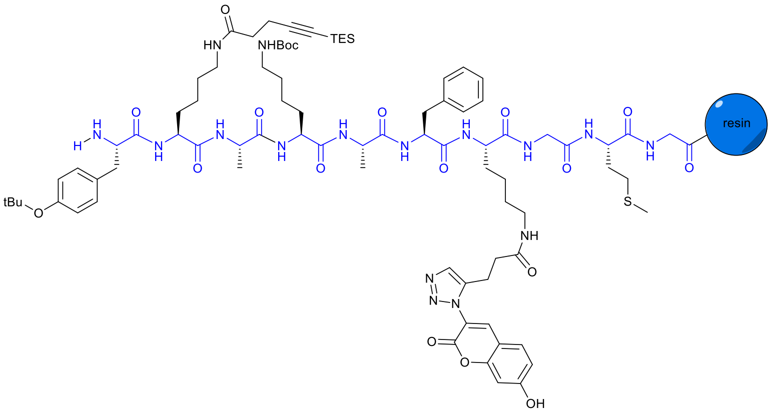

Dry resin was soaked in t-BuOH : H_2O (2 : 3) for 30 min. and drained. The solution of 3-azido-7-hydroxycoumarin (0.4 M in DMSO; 253 μl) was added under argon and syringe was shortly agitated. The coupling solution was prepared in separate vial: to the solution of $CuSO_4$ *5H_2O (0.1M in H_2O; 127 μl) the solution of BTTP (0.05M in t-BuOH, 506 μl) was added. Solution of sodium ascorbate (0.05M in H_2O; 506 μl) was added and the resulting colorless solution was transferred to the resin-azide mixture under argon. The syringe was sealed and gently shaken each approx. 30 min (4x), than stood overnight. The resin was drained, small sample treated with CNBr following the general procedure, and the progress of the synthesis was verified by LC-MS analysis. The click procedure was repeated again with the same amount of reactants and reagents. The resin was washed with DMSO (10x), H_2O (3x), DMSO (3x), DCM (4x) and dried. Yield: 361 mg of loaded resin.

TES removal:

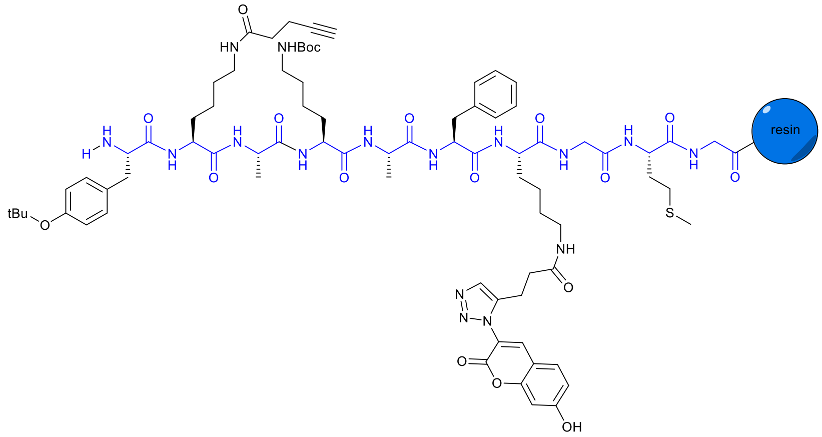

Dried resin was soaked in DMF : MeOH : H_2O (60 : 32 : 8) for 40 min. and drained. The solution of $AgClO_4$ (0.2M in DMF : MeOH : H_2O (60 : 32 : 8); 1956 μl) was added under argon, syringe with resin was slowly rotated for 2 hr with exclusion of light. Resin was drained, washed with DMF (5x), H2O (2x), KCN (0.5 M in H_2O; 4x), H_2O (2x), DMSO (2x), DCM (3x), blow dried with nitrogen, for 60 min. Yield: 337 mg of loaded resin. The progress of the synthesis was verified by LC-MS analysis.

Click II - N-ethyl-N-(2-azidoethyl)-4-(2-chloro-4-nitrophenylazo)phenylamine

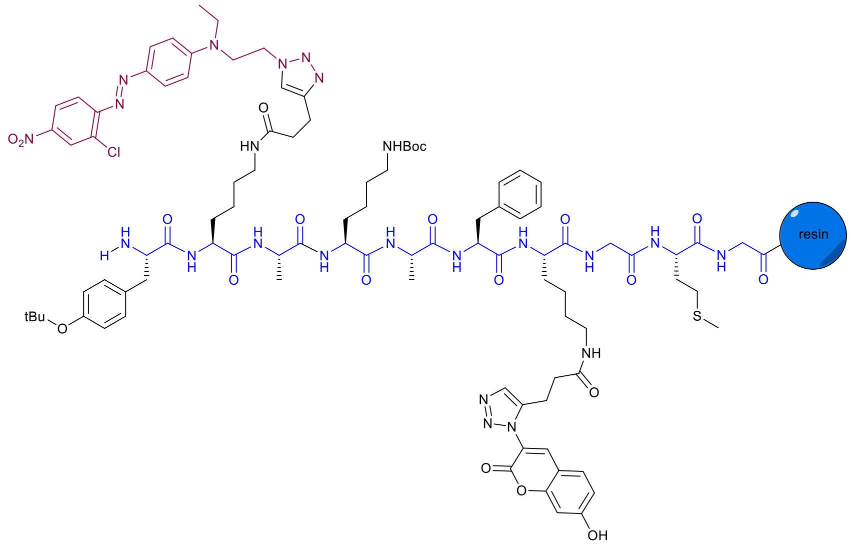

Dry resin was soaked in t-BuOH : H_2O (2 : 3) for 20 min. and drained. The solution of N-ethyl-N-(2-azidoethyl)-4-(2-chloro-4-nitrophenylazo)phenylamine[5] (0.4 M in DMSO; 253 μl) was added under argon and syringe was shortly agitated. The coupling solution was prepared in separate vial: To the solution of $CuSO_4$ *$5H_2O$ (0.1M in H_2O; 127 μl) the solution of BTTP (0.05M in t-BuOH, 506 μl) was added. Solution of sodium ascorbate (0.05M in H_2O; 506 μl) was added and the resulting colorless solution was transferred to the resin-azide mixture under argon. The syringe was sealed and gently rotated overnight. The resin was drained, small sample treated with CNBr following the general procedure, and the progress of the synthesis was verified by LC-MS analysis. The resin was washed with DMSO (25x), DCM (10x) and dried. Yield: 420 mg of loaded resin.

Deprotection of the modified peptide:

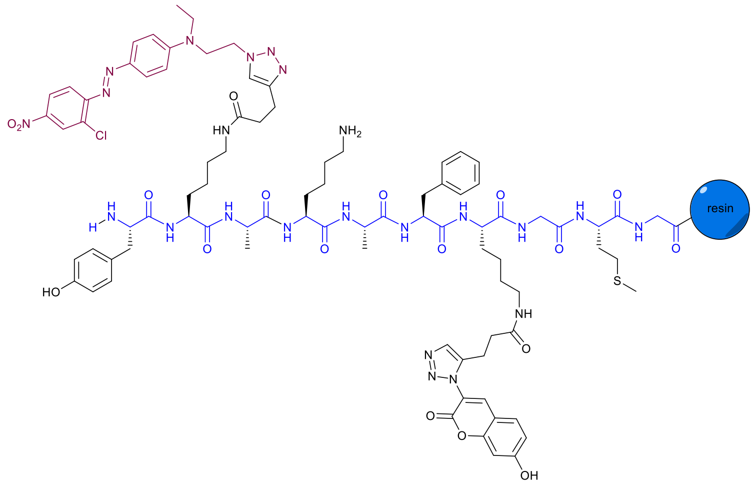

To the dry resin, the mixture of TFA : H_2O : TIS (95 : 2.5 : 2.5; 3 ml) was added. The syringe was gently rotated for 1 hr, TFA cocktail was replaced by a fresh batch (3 ml), reaction mixture was rotated for additional 1 hr, drained, washed with DMSO (5x), DCM (5x), DMSO (3x), H_2O (3x). Small sample was treated with CNBr following the general procedure, and resulting solution was analyzed by LC-MS analysis.

Cleavage-off from the resin:

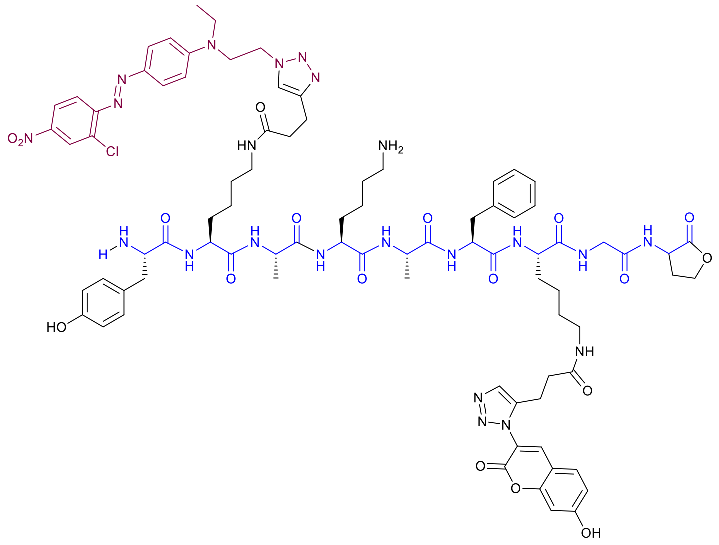

To the resin, soaked in H_2O from previous step, H_2O (2 ml) and CNBr (0.5 M in 0.2M HCl; 2 ml) was added under argon. Syringe was gently rotated overnight. The resin was drained and washed with H_2O. The resin was extracted by warm ACN (approx. 50°C; 3 ml, 3x), DMSO (cca 2 ml), organic fractions were collected and concentrated. HPLC purification (5 $\rightarrow$83 % ACN in H_2O (+ 0.1 % TFA)) afforded 26 mg (34.1 % over whole synthesis, calculated with resin load 0.176 mmol/g) of the modified peptide **Pep11**. HRMS $[M+Na]^+$ m/z calcd. for $[C_{83}ClH_{103}N_{22}NaO_{18}]^+$ 1753.74014, found 1753.74082.

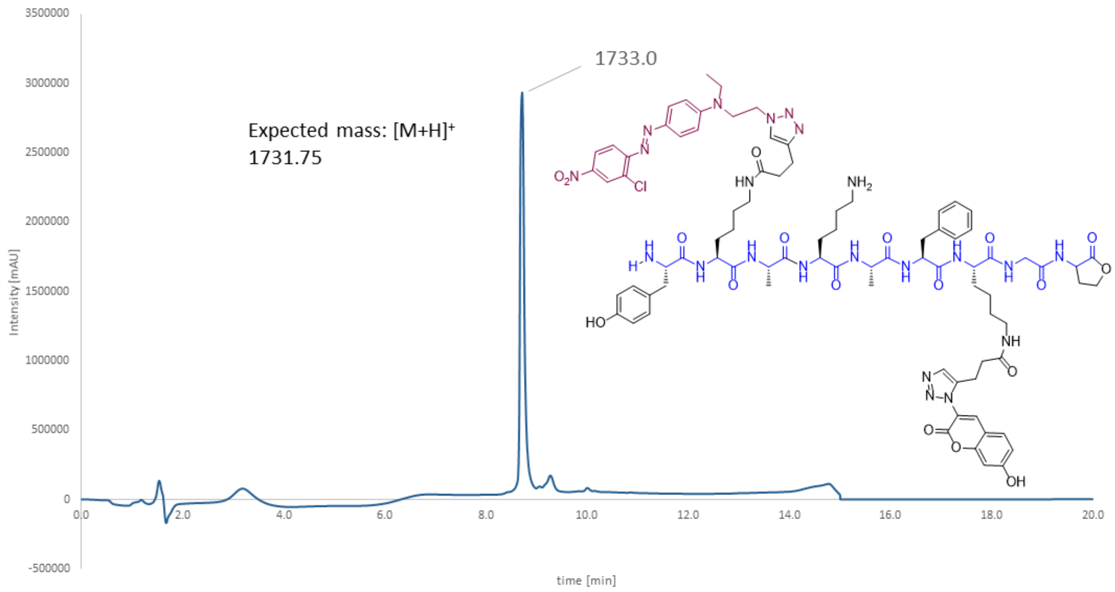

*Figure S5: LC-MS analysis of compound **Pep11** at 214 nm.*

Synthesis of **18** – the positive standard for fluorescence assay

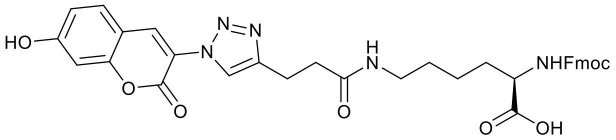

The solution of $CuSO_4$*$5H_2O$ (0.1M in H_2O, 383 µl; 0.038 mmol) was mixed with BTTP (0.05M in t-BuOH, 1.53 ml, 0.077 mmol), than with sodium ascorbate solution (0.05M in H_2O, 1.53 ml, 0.077 mmol). 3-azido-7-hydroxy-2H-chromen-2-one (3-azido-7-hydroxy-coumarin, Santiago) (0.4 M in DMSO, 420.8 µl; 0.168 mmol) was added. To the well stirred solution **1** (0.4 M in DMSO; 382.5 µl; 0.153 mmol) was dropped, reaction mixture was stirred for 2h and stored for 14h at -12 ºC for 14h. Liquid was discarded, remaining syrupy residue was extracted with DCM (2x), EDTA (1x), H_2O (1x), EtOH (1x) and concentrated. Crude residue was taken into DMF (3 ml), precipitated with Et_2O (30 ml), centrifuged and dried. Yield: 104 mg, (dark green foam) $[M+Na]^+$ m/z calcd. for $[C_{35}H_{33}\ O_8N_5Na]^+$ 674.22213, found 2674.2188.

^{1}H NMR spectra of **18** showed very broad signals that made signal assignment impossible. In order to improve spectrum resolution, we measured 1H and 13C spectra at elevated temperatures (50, 80, 100 °C), however we observed decomposition of the sample. Therefore, identity and purity of Fmoc-protected Pep12 was proven by HPLC/MS (see below).

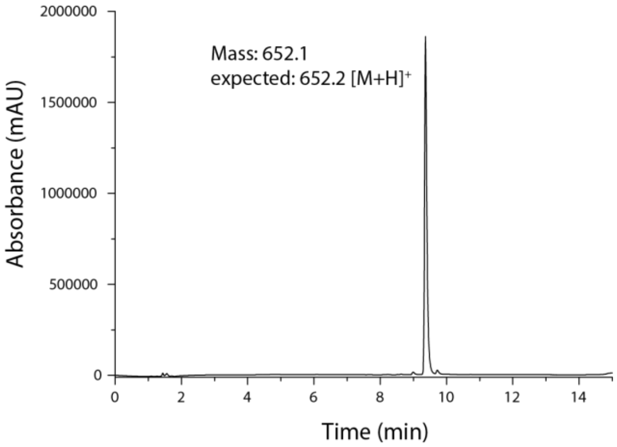

HPLC chromatogram of compound **18** used as positive control during trypsin digestion experiment with **Pep11**. The observed and expected mass is indicated.

Methods optimizations:

Optimization of TES removal in the presence of TIPS:

Based on lit.[16]

In order to find optimal conditions for selective removal of TES protective group in presence of TIPS, Initially tested in solution, various substrate/silver salts ratios were used to reach maximal efficiency. $AgNO_3$, $AgClO_4$ and AgF were selected as promising cleavage agents. The other silver salts were either of limited efficiency ($AgNO_2$) or not effective at all (AgOCN, Ag_2SO_4) at given concentration. In our hands, bases (1,8-Diazabicyclo[5.4.0]undec-7-ene (DBU), Cs_2CO_3) in combination with MeOH did not give useful results.

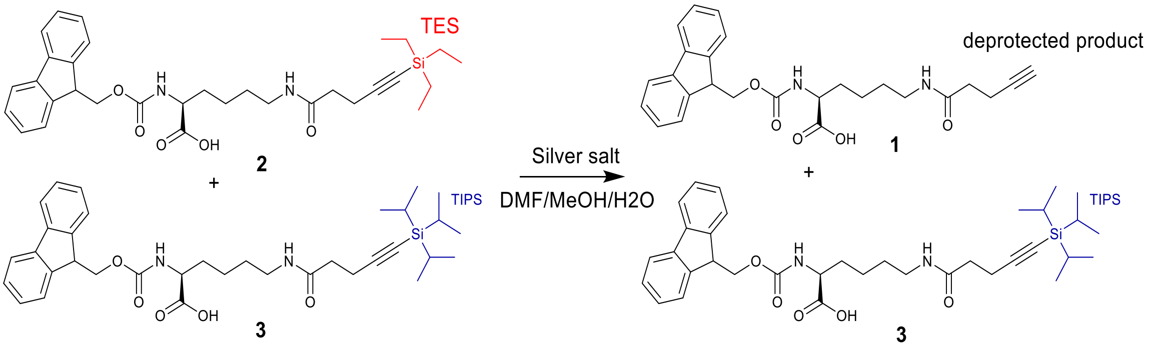

Scheme S7: Orthogonal removal of TES group using equimolar mixture of modified amino acids **2** and **3**.

Sample preparation: In the 200 µl HPLC sample vial, **2** (0.8 µmol; 1 eq.) and **3** (if used - 0.8 µmol; 1 eq.) were dissolved in DMF (60 µl). Silver salt (0.1-10 eq, in MeOH : H_2O (4 : 1); 40 µl) was added and the reaction mixture was monitored on LC-MS system (Shimadzu) in indicated time points. Equivalents of salt marked in graph headings are related to amino acid **2**.

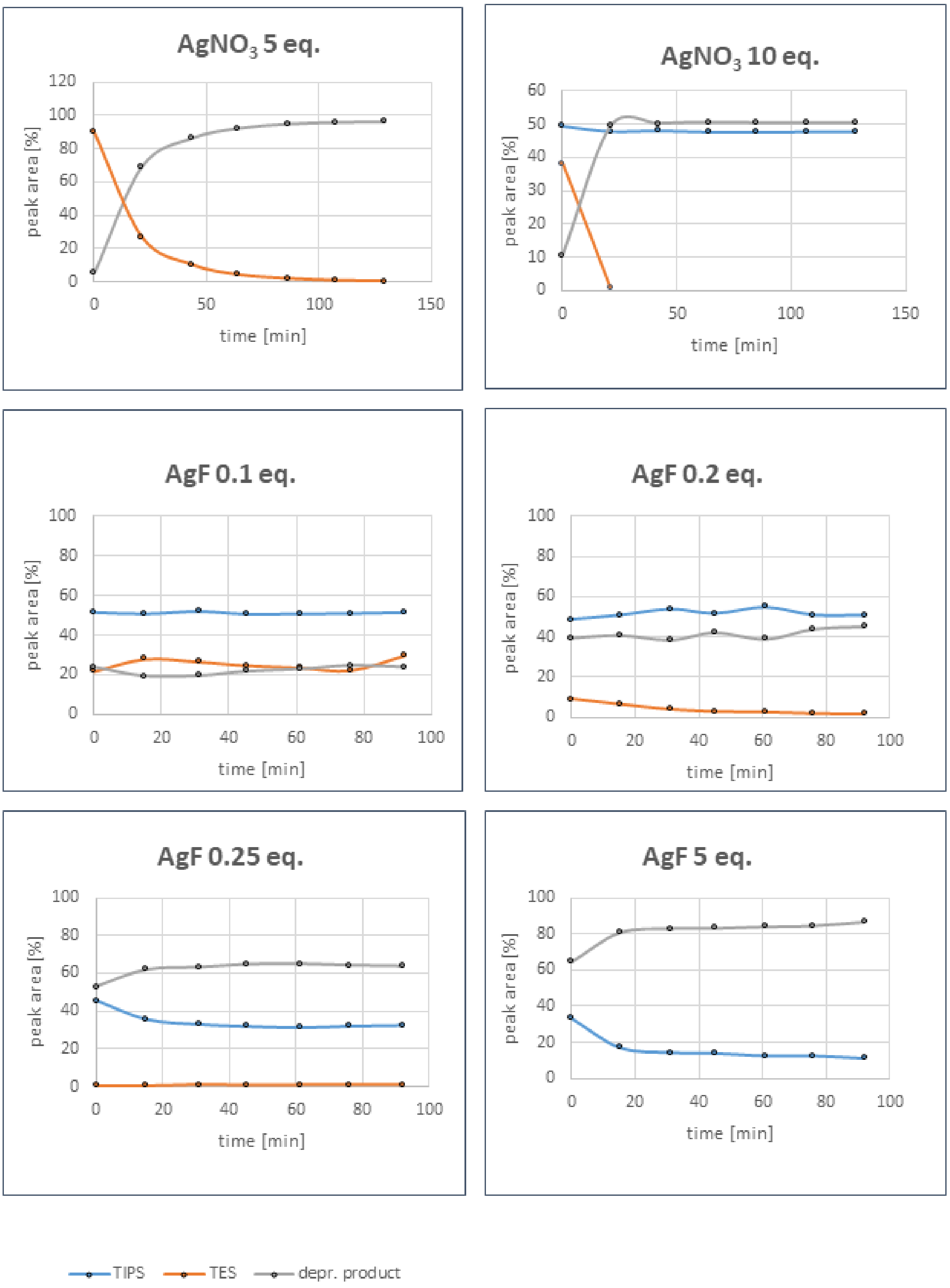
AgNO$_3$ 5 eq.
AgNO$_3$ 10 eq.
AgF 0.1 eq.
AgF 0.2 eq.
AgF 0.25 eq.
AgF 5 eq.
peak area [%]
time [min]
TIPS
TES
depr. product

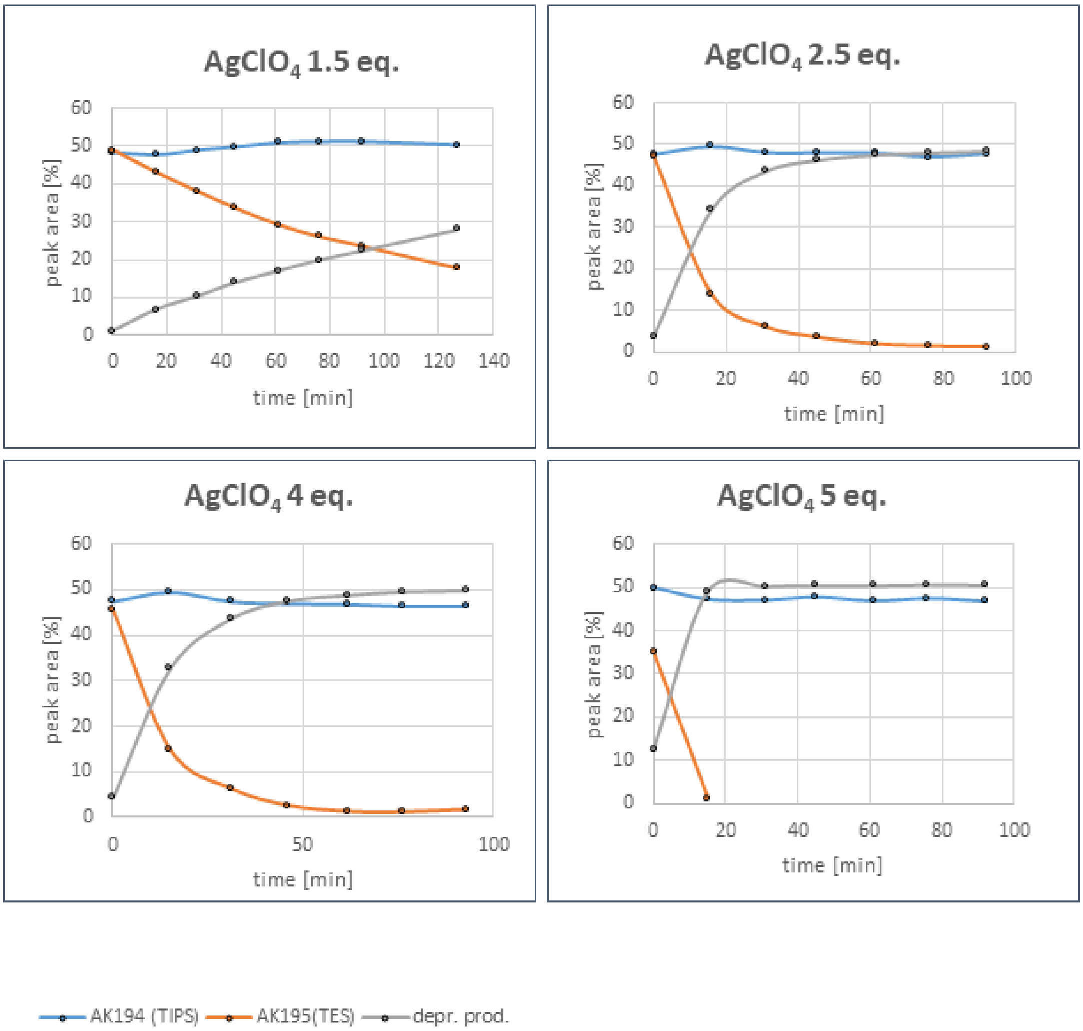

Figure S6: LC-MS traced selective removal of triethylsilyl (TES) protective group in solution. To achieve semiorthogonality between TES and triisopropylsilyl protective group (TIPS), optimal conditions were investigated. Concentration of both TES and TIPS derivatives was 8 mmol/l in solvent mixture DMF : MeOH : H_2O (60 : 32 : 8). Crude LC-MS data were processed, integrated peak areas were plotted.

Optimization of TES removal from resin bound peptides:

As $AgClO_4$ showed up to be the most convenient cleaving agent for removal of TES in the presence of TIPS, we used it to establish the procedure for semiorthogonal deprotection of resin-bound glycopeptides. We prepared the model resin-bound alkyne-modified peptides **Pep14** (TES), **Pep15** (TIPS), and **Pep16** (TES + TIPS) and exposed them to $AgClO_4$ solutions of varying concentrations. Deprotected peptides were cleaved-off from the resin and analyzed by LC-MS (Shimadzu).

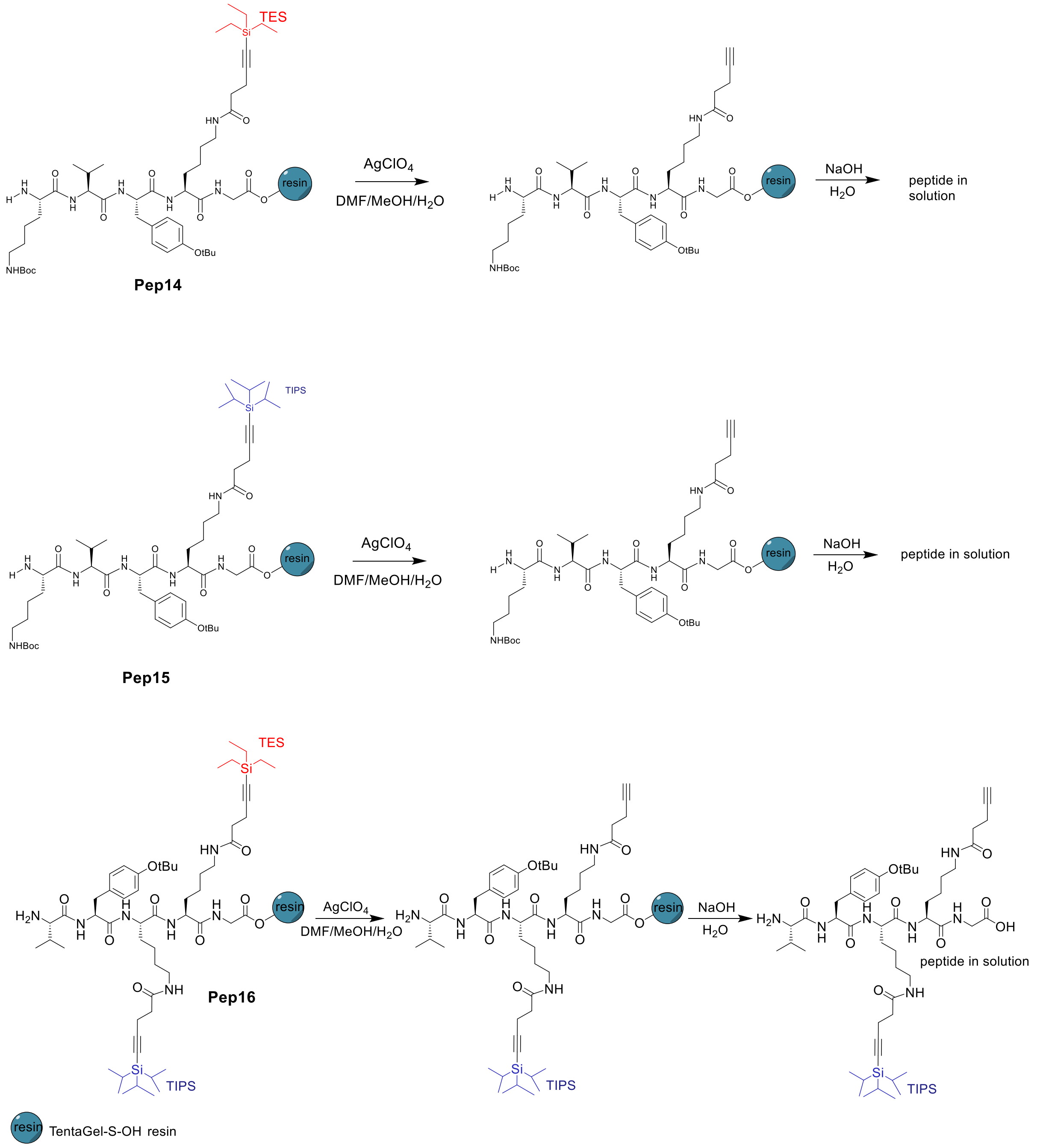

Scheme S8: Orthogonal TES removal on resin-bound model substrates **Pep14**, **Pep15** (used as the control to confirn the TIPS stability under investigated conditions) and **Pep16**, modified both with TES and TIPS protected terminal alkynes

Typical procedure:

Sample of resin-bound peptide (2 mg; 0.37 μmol) was soaked in DMF : MeOH : H_2O (60 : 32 : 8) for 20 min., drained, the solution of $AgClO_4$ (of given concentration in DMF : MeOH : H_2O (60 : 32 : 8); 10 μl) was added, reaction vial was flushed with argon and gently shaken each 15 min for overall time of 2h with exclusion of light. The resin was drained, washed with DMF : MeOH : H_2O (60 : 32 : 8, 5x), KCN (0.1M in H_2O; 4x), H_2O (3x), and drained. For cleavage of the peptide from the TentaGel-S-OH resin, the solution of NaOH (0.1M in H_2O; 30 μl) was added, reaction mixture was shaken for 10 min.,

neutralized by HCl (0.2M in H_2O) and the liquids were collected. Drained resin was washed with warm ACN (2x), ACN washes were mixed with previously collected liquids and analyzed by LC-MS.

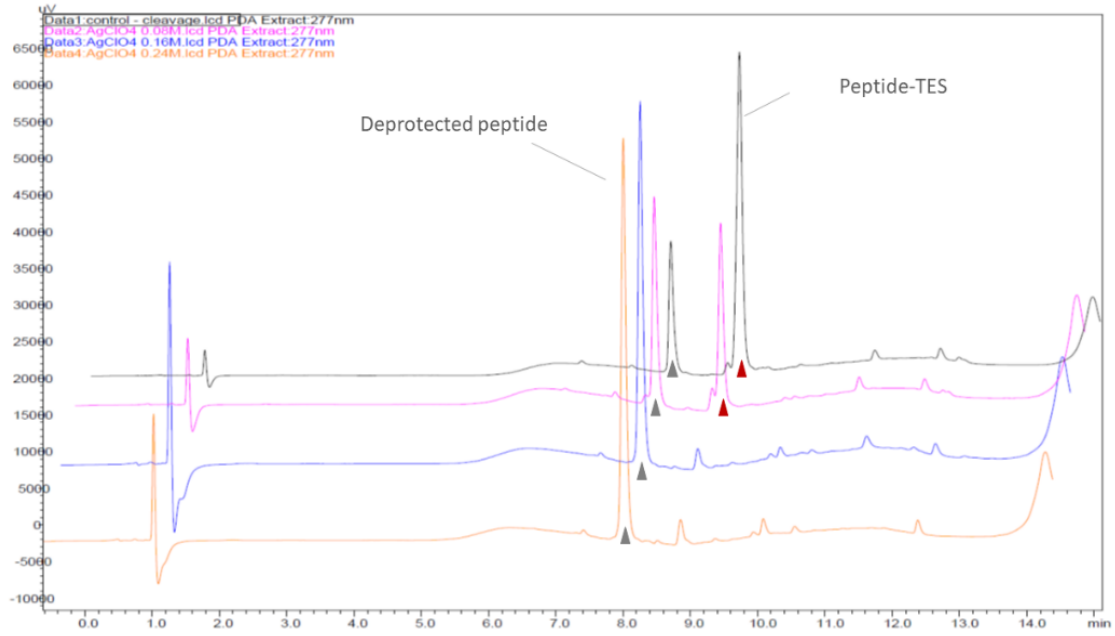

*Figure S7: Deprotection of resin bound TES-containing peptide **Pep14** using different concentrations of $AgClO_4$ for 2h in DMF:MeOH:H_2O (60:32:8). The figure shows HPLC chromatograms of the crude peptide after cleavage from the resin using 0.1M NaOH. Crude peptide without treatment with $AgClO_4$ (black line), using 0.08M $AgClO_4$ (purple line), using 0.16M $AgClO_4$ (blue line) and using 0.24M $AgClO_4$ (orange line). Complete deprotection was observed using 0.16M solution of $AgClO_4$. Note. The TES group is not completely stable to the clevage conditions (0.1M NaOH for 90 min) and therefore the crude peptide (black line) also contains the deprotected peptide.*

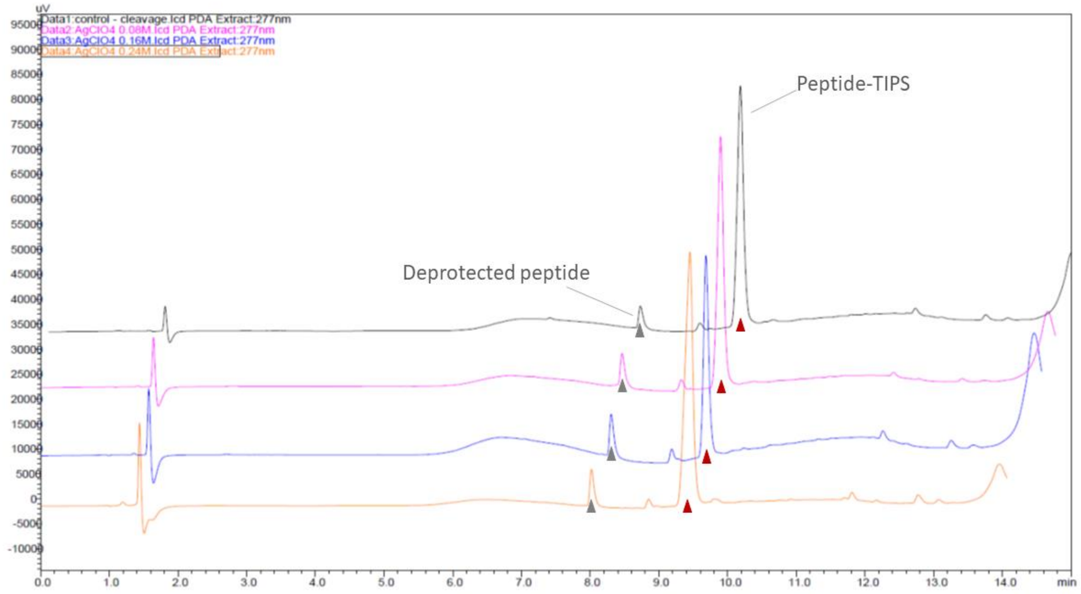

*Figure S8: Deprotection of resin bound TES-containing peptide **Pep15** using different concentrations of $AgClO_4$ for 2h in DMF : MeOH : H_2O (60 : 32 : 8). The figure shows HPLC chromatograms of the crude peptide after cleavage from the resin using 0.1M NaOH. Crude peptide without treatment with $AgClO_4$ (black line), using 0.08M $AgClO_4$ (purple line), using 0.16M $AgClO_4$ (blue line) and using 0.24M $AgClO_4$ (orange line). The TIPS group is stable towards $AgClO_4$. Note. The TIPS group is not completely stable to the clevage conditions (0.1M NaOH for 90 min) and therefore the crude peptide (black line) also contains traces of the deprotected peptide.*

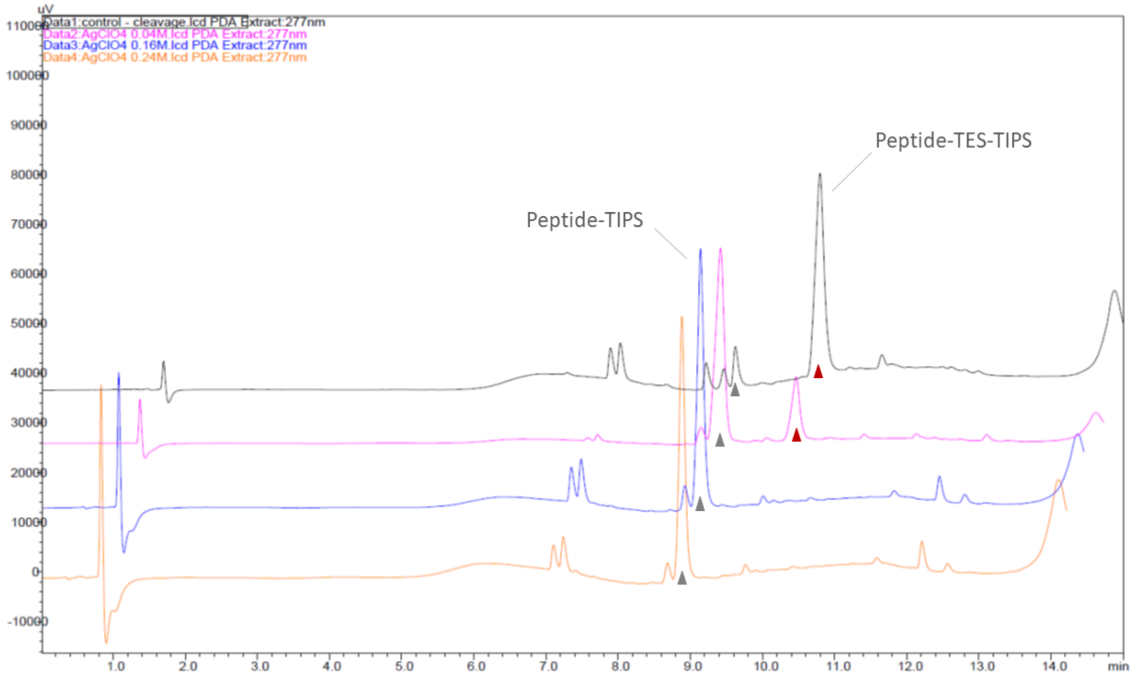

*Figure S9: Conversion of TES removal from resin-bound peptide **Pep16**, traced by LC-MS after cleavage-off. Concentrations of $AgClO_4$ solutions: 0.08M, 0.16M, and 0.24M in DMF:MeOH:H_2O (60:32:8), 5ml/g or dry substitued resin, 2h. TES group was once more fully removed by 0.16M solution of $AgClO_4$.*

Optimization of CuAAC reaction for resin-bound peptides:

In order to find the optimal conditions to modify peptides by CuAAC on solid phase, model resin-bound heptapeptide **Pep1** was "clicked" with saccharide analog **Gal-C3-N$_3$**. In our hands, the first modification of peptide backbone was the most challenging one. LC-MS was used to analyze reaction mixture after product was cleaved-off from the resin, conversion was determined from peak areas of product **Pep17** and remaining unmodified **Pep1**.

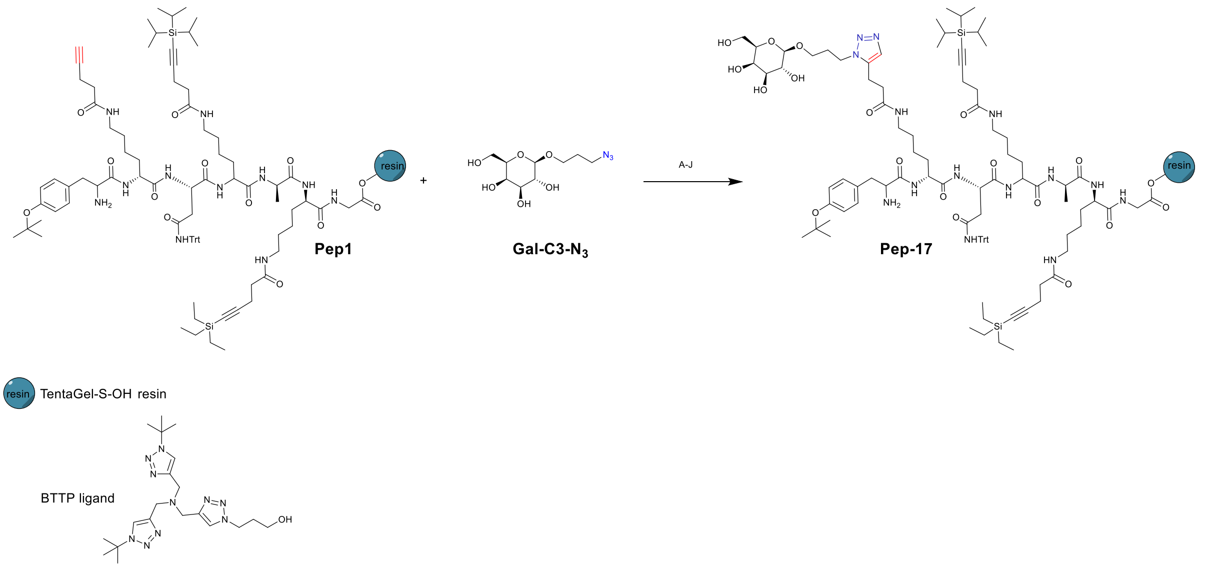

Scheme 9: Model reaction used for optimisation of first CuAAC click reaction on solid phase

Typical procedure:

Resin-bound **Pep1** (5 mg; 0.77 μmol) was soaked in solvent mixture for 30 min and drained. The solution of 3-azidopropyl β-D-galactopyranoside **Gal-C3-N$_3$** (0.4 M in H_2O; 3.83 μl; 1.53 μmol) was added and vial was thoroughly agitated. The click activation solution was prepared in separate vial: to the solution of $CuSO_4$ *5H_2O (0.1M in H_2O; 1.91 μl; 0.19 μmol) the solution of BTTP (0.05M in t-BuOH; 7.65 μl; 0.38 μmol) was added, solution of sodium ascorbate (0.05M in H_2O; 7.65 μl; 0.38 μmol) was added and the resulting colorless solution was transferred to the resin-azide mixture. Detergent or base was added (if relevant), the vial was flushed with argon, sealed and gently rotated for 24h. The resin was drained, washed with used solvent mixture (2x), DMSO (5x), H2O (3x) and drained. For cleavage of the peptide from the TentaGel-S-OH resin, the solution of NaOH (0.1M in H_2O; 50 μl) was added, reaction mixture was shaken for 10 min., neutralized by HCl (0.2M in H_2O) and the liquids were collected. Drained resin was washed with warm ACN (2x), ACN washes were mixed with previously collected liquids and analyzed by LC-MS.

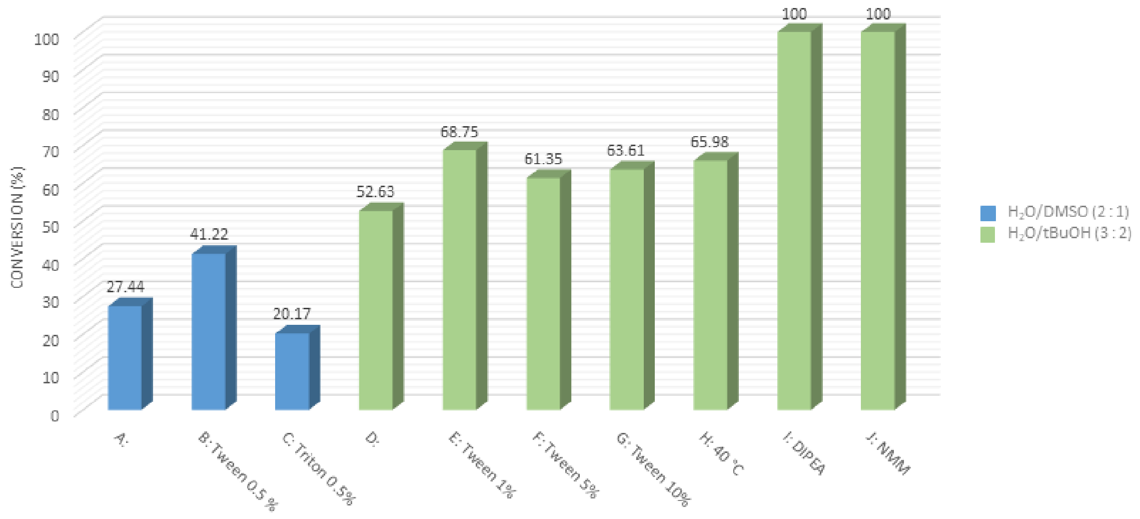

*Figure S10: Click modification of resin bound **Pep1** with **Gal-C3-N_3** by CuAAC following general procedure described above (2 equiv. of **Gal-C3-N_3**, 25mol% of $CuSO_4$, 50mol% of BTTP ligand and 50mol% of sodium ascorbate). The conversion of the peptide was followed by HPLC-MS after cleavage of the peptide from the resin by 0.1M NaOH. **A**) performed in DMSO : H_2O (1 : 2); **B**) performed in DMSO : H_2O (1 : 2) + 0.5% Tween 20; **C**) performed in DMSO : H_2O (1 : 2) + 0.5% Triton X; **D**) performed in t-BuOH : H_2O (2 : 3); **E**) performed in t-BuOH : H_2O (2 : 3) + 1% Tween 20; **F**) performed in t-BuOH : H_2O (2 : 3) + 5% Tween 20; **G**) performed in t-BuOH : H_2O (2 : 3) + 10% Tween 20; **H**) performed in t-BuOH : H_2O (2 : 3) and heated up at 40°C; **I**) performed in t-BuOH : H_2O (2 : 3) + DIPEA (2.2 equiv.); **J**) performed in t-BuOH : H_2O (2 : 3) + NMM (2.2 equiv.).*

.

Fluorescence assay – Cleavage of fluorogenic substrate **Pep11** by trypsin

Peptide **Pep11** was subjected to the brief "proof-of-concept" assay with trypsin. Enzyme trypsin is able to cleave peptide chains after polar amino acid residues (arginine and lysine). The peptide **Pep11**, contains the fluorophore and the quencher molecule in close proximity so that the fluorescence of the coumarin is quenched. Cleavage of the peptide **Pep11** by trypsin after the remaining lysine residue yields two short peptide sequences where the two molecules (the dye and the quencher) are apart. As consequence the fluorescence of the free coumarin can be observed and detected.

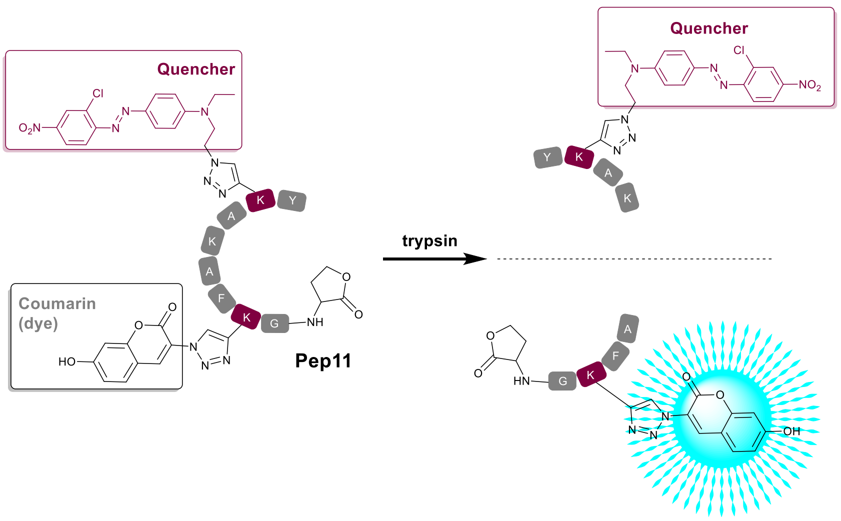

Controls:

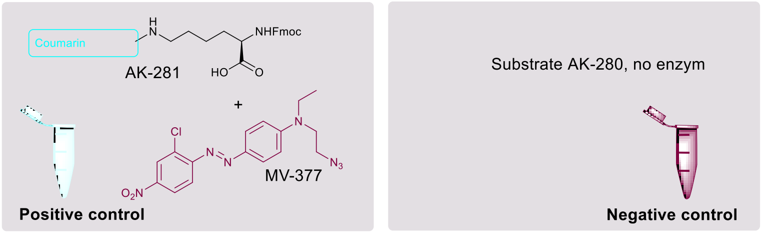

Scheme S10: Trypsine cleavage of fluorogenic peptide AK-280

Conditions:

The fluorescence assay of modified peptide **Pep11** cleaved by Trypsin (Trypsin Gold, Promega) was performed at 37°C in 96-well non-treated flat bottom half area black polystyrene plates (Corning). The conditions were as follows: 50 mM HEPES, pH 7.4, 150 mM NaCl, 5 % v/v DMSO, 0.02 % (w/v; corresponds to enzyme : substrate ratio 1 : 100) or 0.01 % (w/v; corresponds to enzyme : substrate ratio 1 : 200) trypsin, 10 µM fluorogenic peptide substrate in a final volume of 50 µl. Fluorescence was read continuously in a plate reader (Tecan Spark®). Excitation and emission wavelenghts were 404 and 477 nm. Controls: same conditions. Negative controls: no enzyme. Positive controls: fluorogenic substrate was surrogated with the mixture of **18** and **19** (1 : 1).

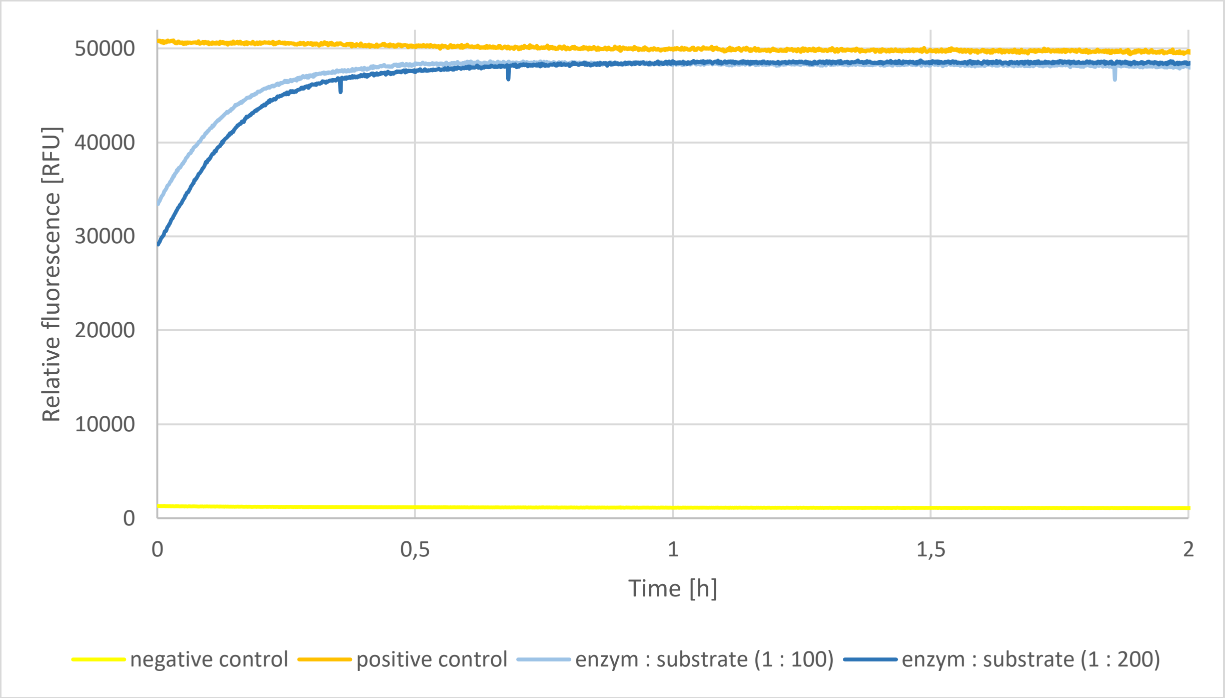

Figure S11: Fluorescence readings of ***Pep11****– typsin assay. Concentration of fluorogenic substrate 10 µM, measured at 477 nm.*

Copies of NMR spectra

Compound **4**

Parameter	Value
Solvent:	CDCl3
Temperature:	298.2018
Nucleus:	1H

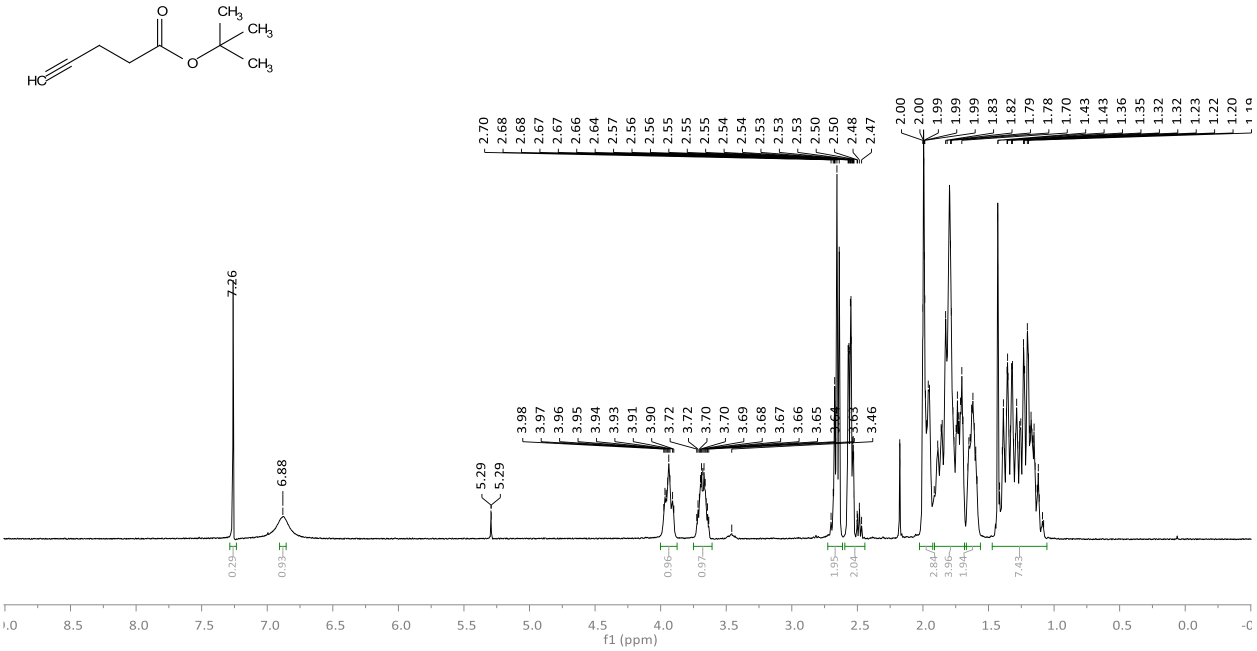

Parameter	Value
Solvent:	CDCl3
Temperature:	298.1967
Nucleus:	13C

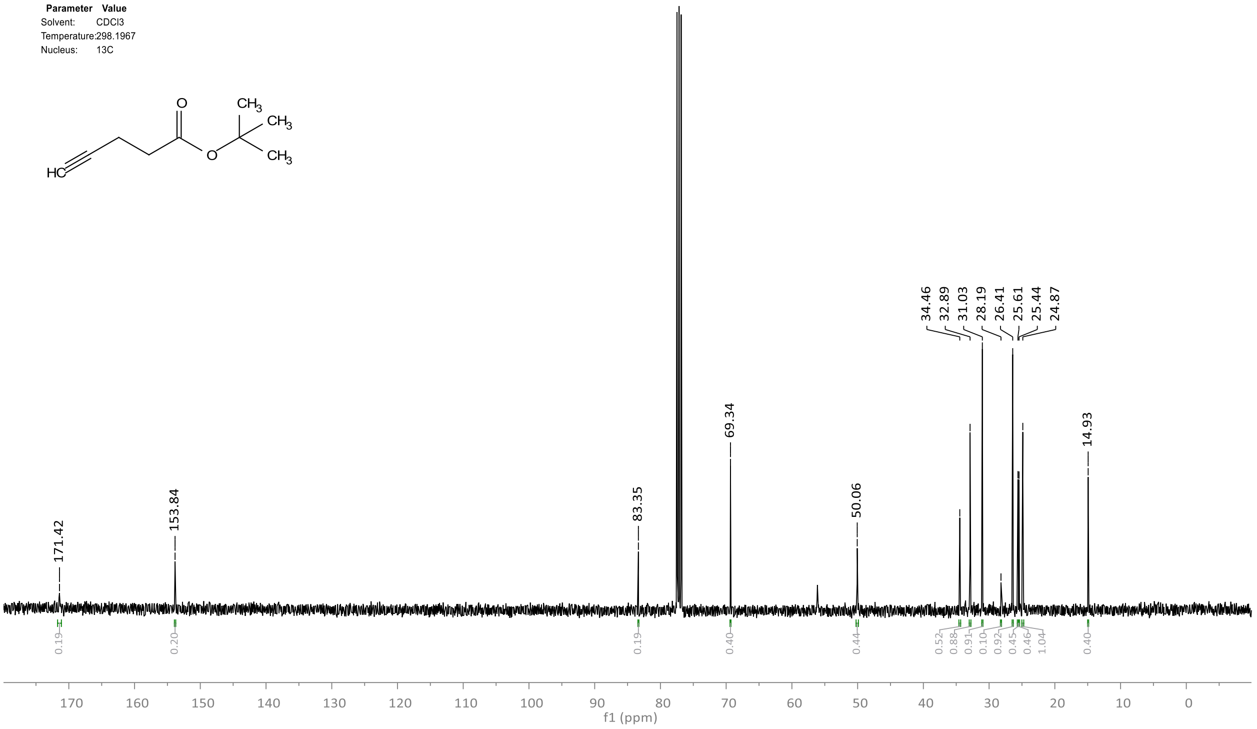

Compound 7

Parameter Value
Solvent: CDCl3
Temperature: 298.0031
Nucleus: 1H

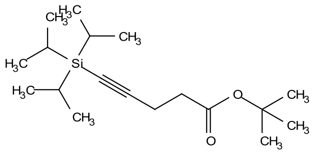

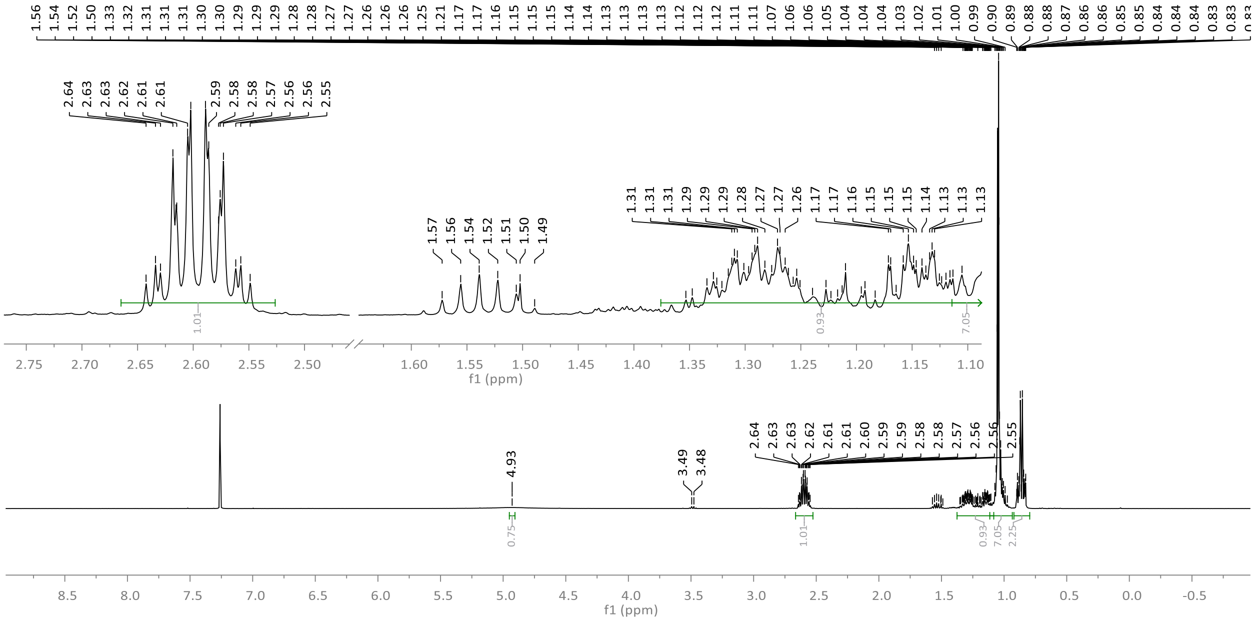

Parameter Value
Solvent: CDCl3
Temperature: 297.998
Nucleus: 13C

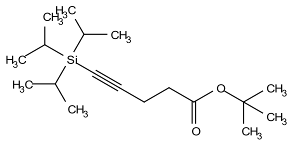

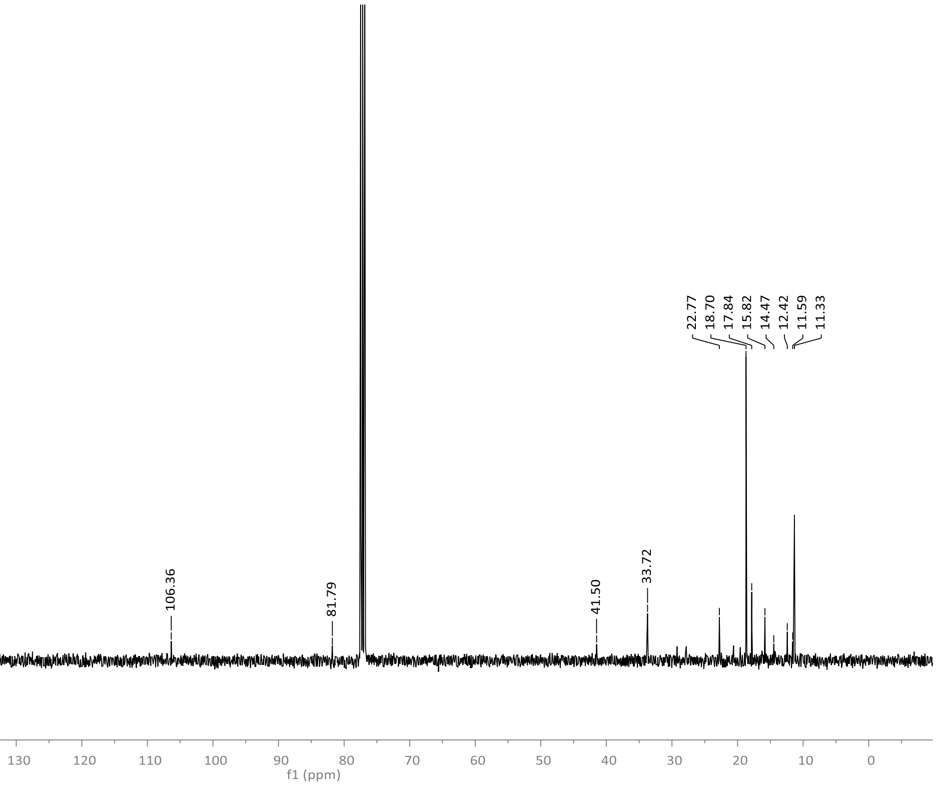

Compound 8

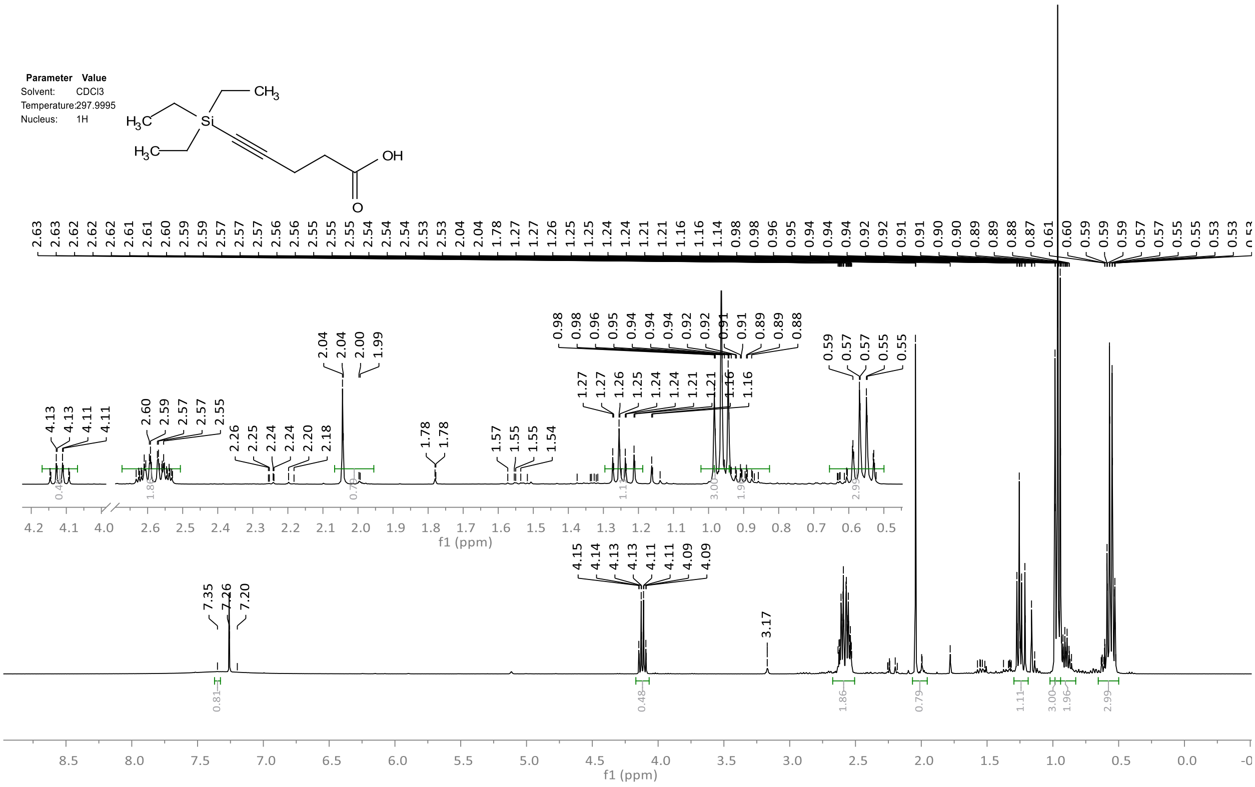
Parameter Value
Solvent: CDCl3
Temperature:297.9995
Nucleus: 1H
f1 (ppm)

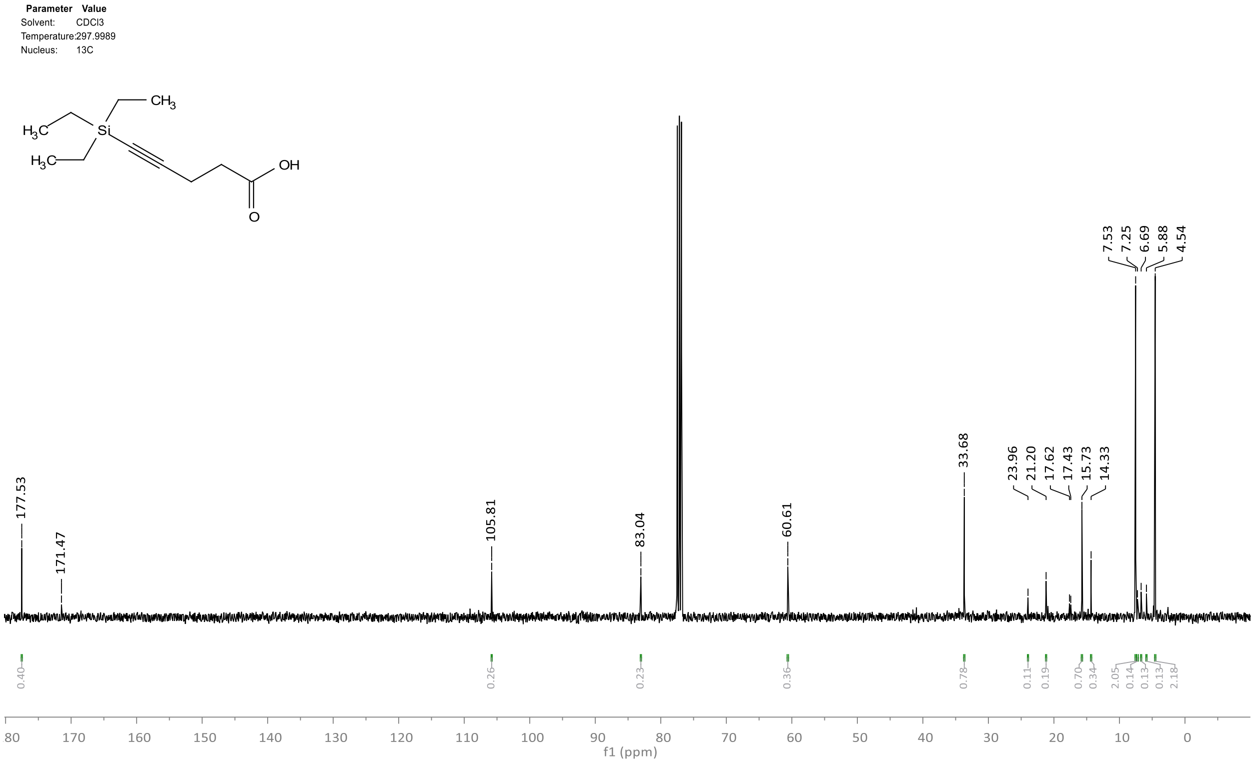
Parameter Value
Solvent: CDCl3
Temperature:297.9989
Nucleus: 13C
177.53
171.47
105.81
83.04
60.61
33.68
23.96
21.20
17.62
17.43
15.73
14.33
7.53
7.25
6.69
5.88
4.54
f1 (ppm)

Compound **16**

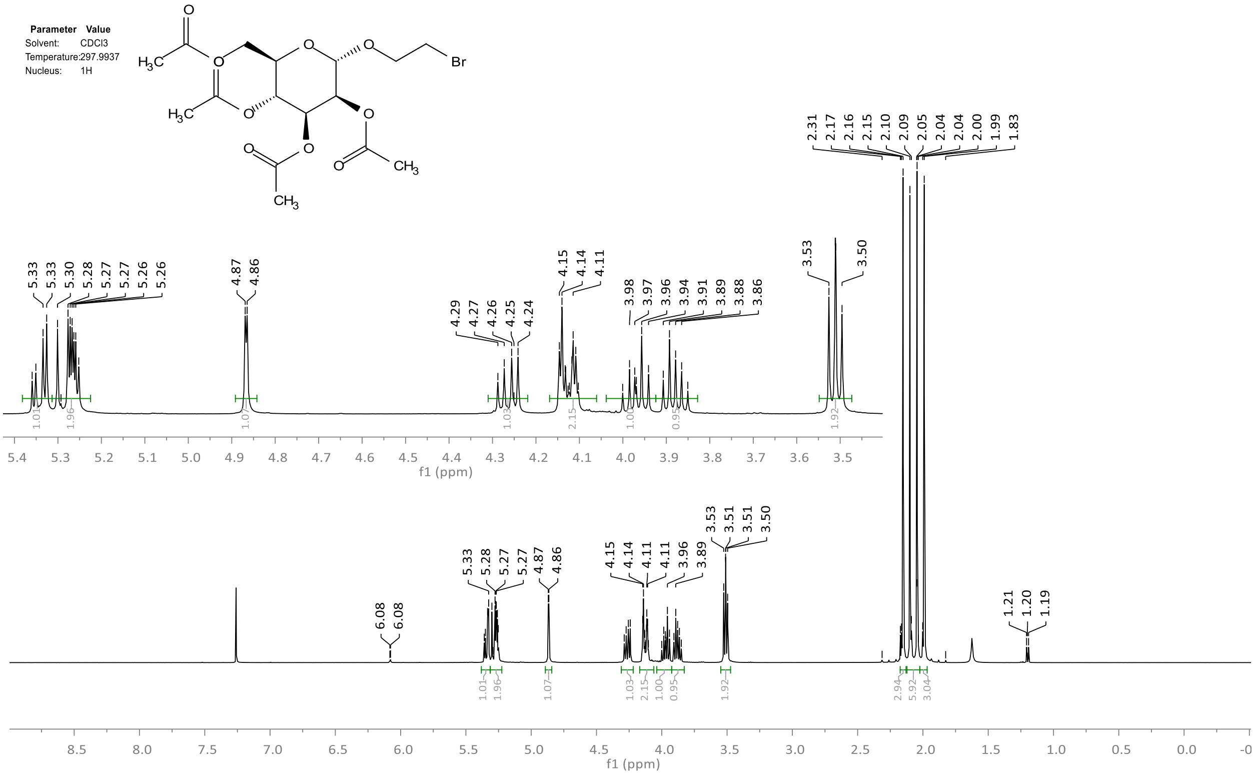
Parameter Value
Solvent: CDCl3
Temperature:297.9937
Nucleus: 1H
f1 (ppm)

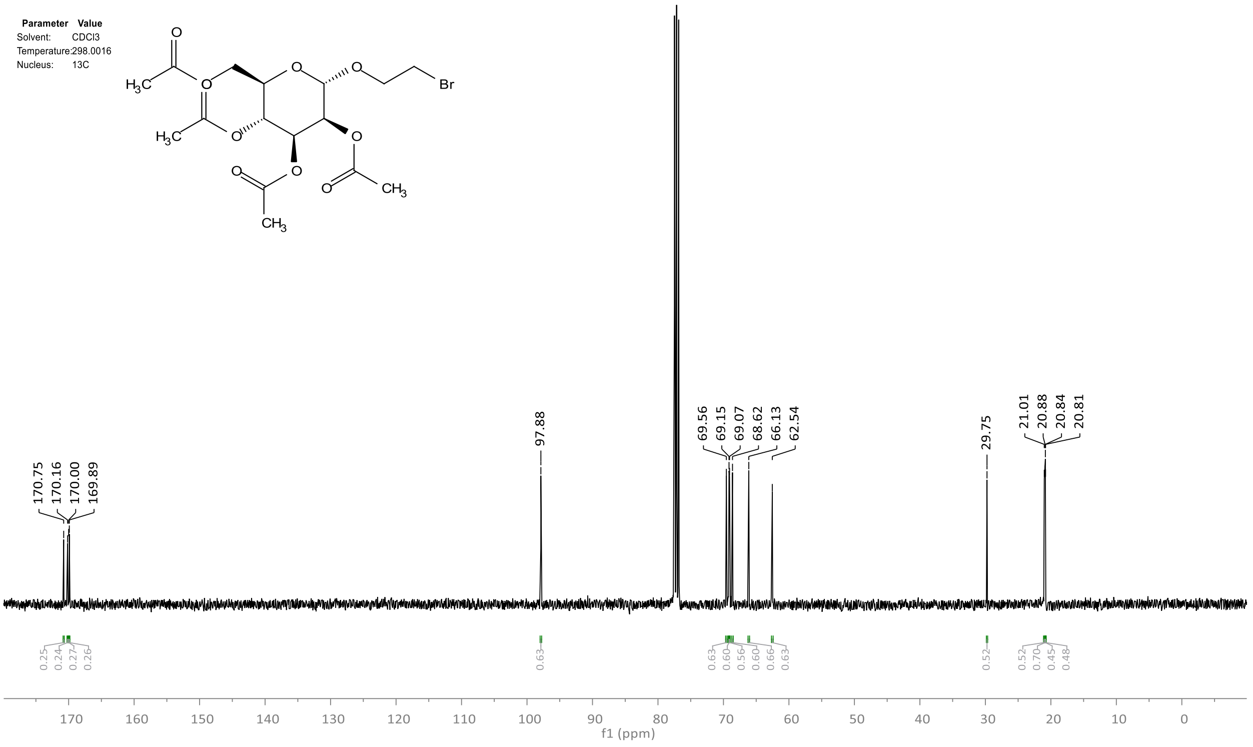
Parameter Value
Solvent: CDCl3
Temperature:298.0016
Nucleus: 13C
f1 (ppm)

Compound 19

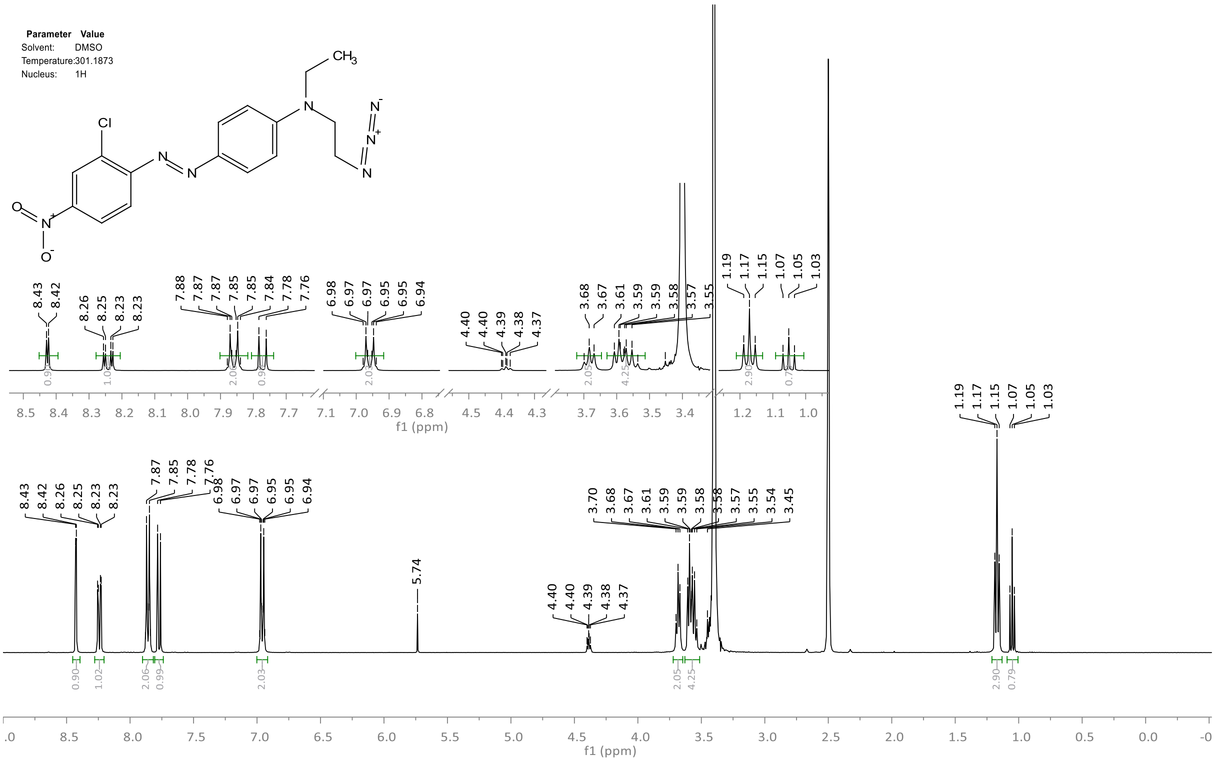
Parameter Value
Solvent: DMSO
Temperature:301.1873
Nucleus: 1H
CH3
Cl
N
N
N
N+
N-
O
O-
8.43 8.42 8.26 8.25 8.23 8.23 7.88 7.87 7.87 7.85 7.85 7.84 7.78 7.76 6.98 6.97 6.97 6.95 6.95 6.94 4.40 4.40 4.39 4.38 4.37 3.68 3.67 3.61 3.59 3.59 3.58 3.57 3.55 1.19 1.17 1.15 1.07 1.05 1.03
3.70 3.68 3.67 3.61 3.59 3.59 3.58 3.58 3.57 3.55 3.54 3.45
5.74
0.90 1.02 2.06 0.99 2.03 2.05 4.25 2.90 0.79
f1 (ppm)

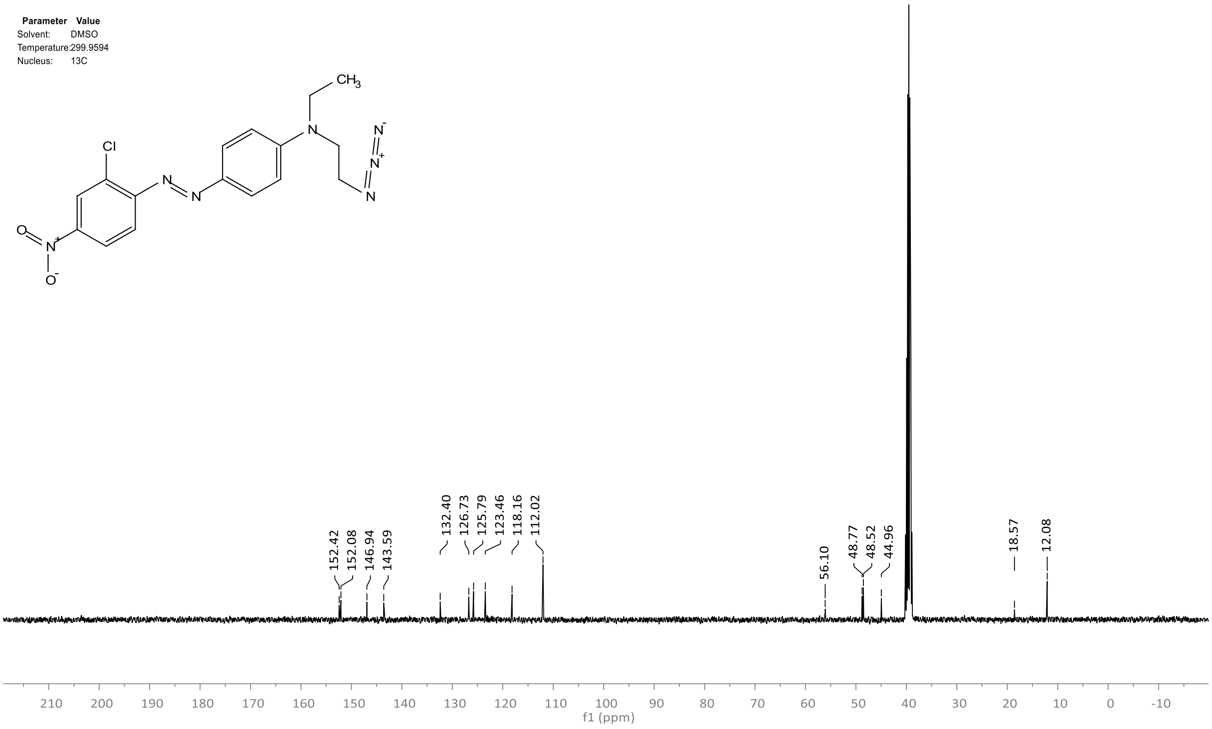
Parameter Value
Solvent: DMSO
Temperature:299.9594
Nucleus: 13C
CH3
Cl
N
N
N
N+
N-
O
O-
152.42 152.08 146.94 143.59 132.40 126.73 125.79 123.46 118.16 112.02 56.10 48.77 48.52 44.96 18.57 12.08
f1 (ppm)

Compound **5**

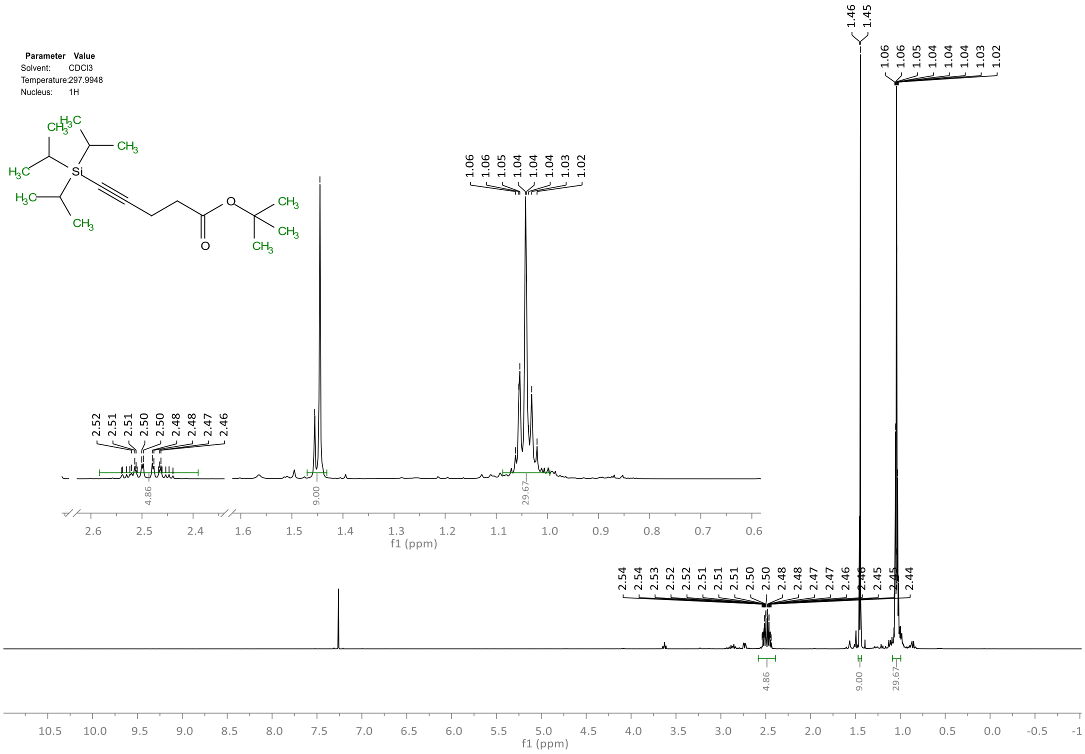
Parameter Value
Solvent: CDCl3
Temperature:297.9948
Nucleus: 1H
1.46
1.45
1.06
1.06
1.05
1.04
1.04
1.04
1.03
1.02
2.52
2.51
2.51
2.50
2.50
2.48
2.48
2.47
2.46
4.86
9.00
29.67
f1 (ppm)
2.54
2.54
2.53
2.52
2.52
2.51
2.51
2.51
2.50
2.50
2.48
2.48
2.47
2.47
2.46
2.46
2.45
2.45
2.44

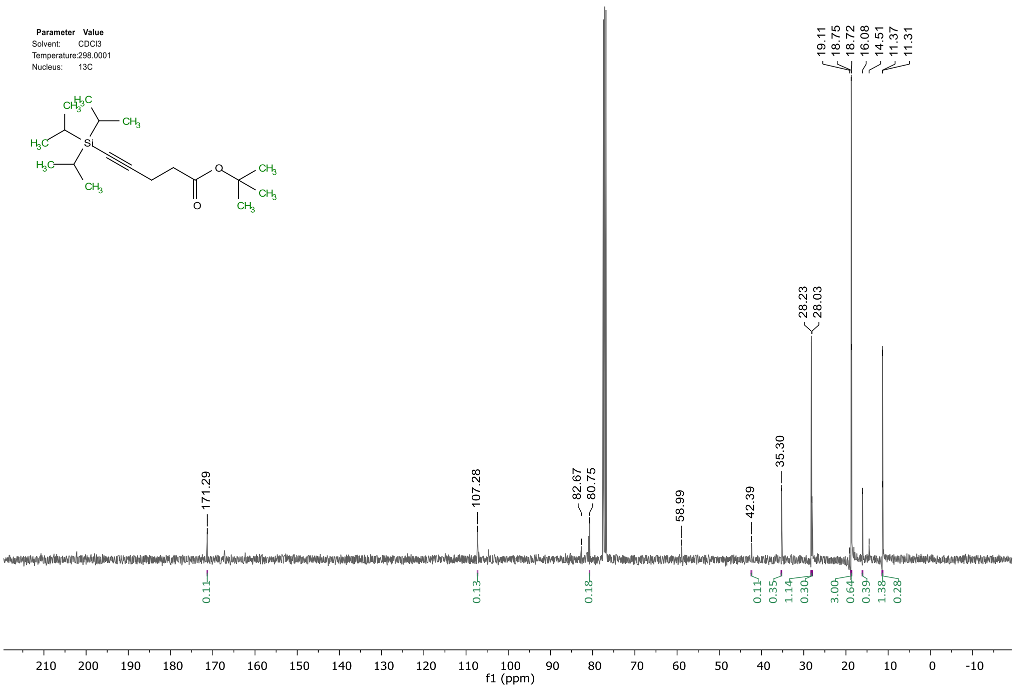
Parameter Value
Solvent: CDCl3
Temperature:298.0001
Nucleus: 13C
19.11
18.75
18.72
16.08
14.51
11.37
11.31
28.23
28.03
35.30
171.29
107.28
82.67
80.75
58.99
42.39
0.11
0.13
0.18
0.11
0.35
1.14
0.30
3.00
0.64
0.39
1.38
0.28
f1 (ppm)

Compound **6**

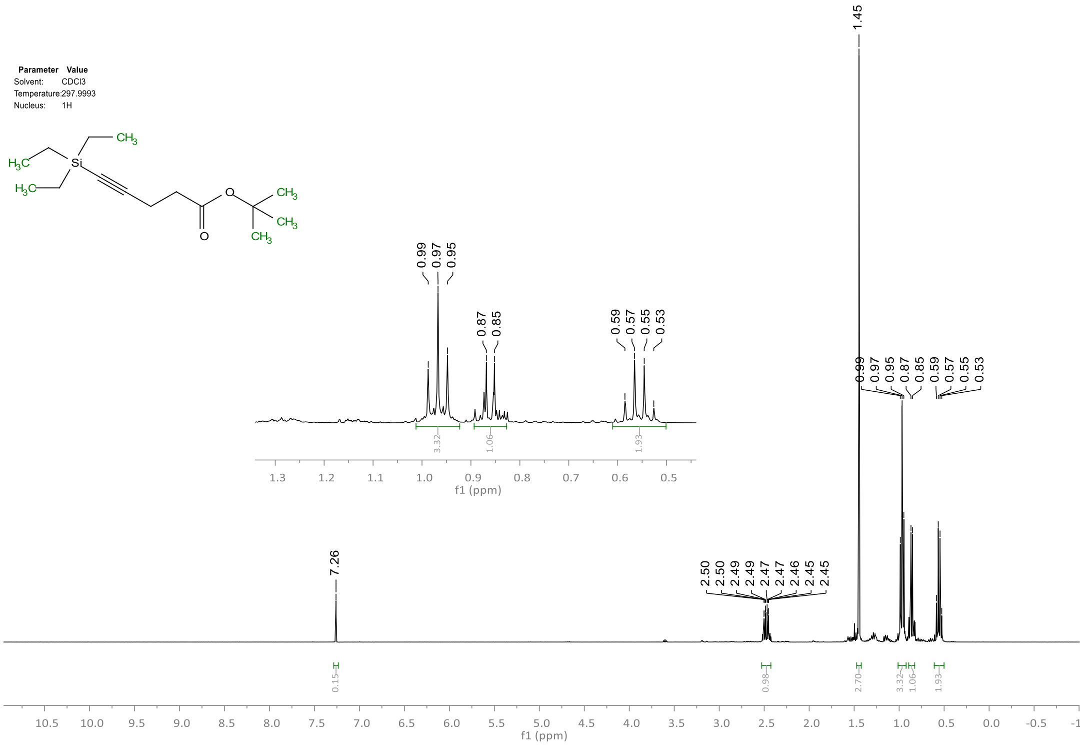
Parameter Value
Solvent: CDCl3
Temperature:297.9993
Nucleus: 1H
CH3
H3C
Si
H3C
O
CH3
CH3
O
CH3
1.45
0.99
0.97
0.95
0.87
0.85
0.59
0.57
0.55
0.53
7.26
2.50
2.50
2.49
2.49
2.47
2.47
2.46
2.45
2.45
0.15
0.98
2.70
3.32
1.06
1.93
f1 (ppm)

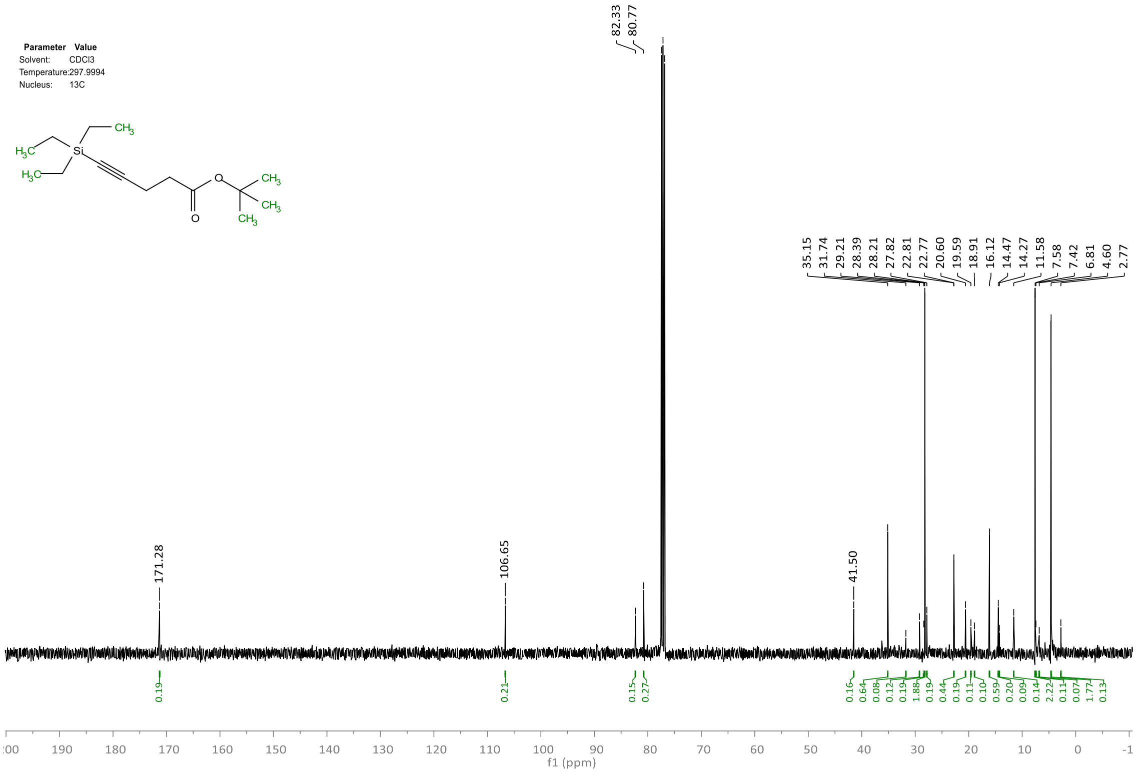
Parameter Value
Solvent: CDCl3
Temperature:297.9994
Nucleus: 13C
CH3
H3C
Si
H3C
O
CH3
CH3
O
CH3
82.33
80.77
171.28
106.65
41.50
35.15
31.74
29.21
28.39
28.21
27.82
22.81
22.77
20.60
19.59
18.91
16.12
14.47
14.27
11.58
7.58
7.42
6.81
4.60
2.77
f1 (ppm)

Compound **11**

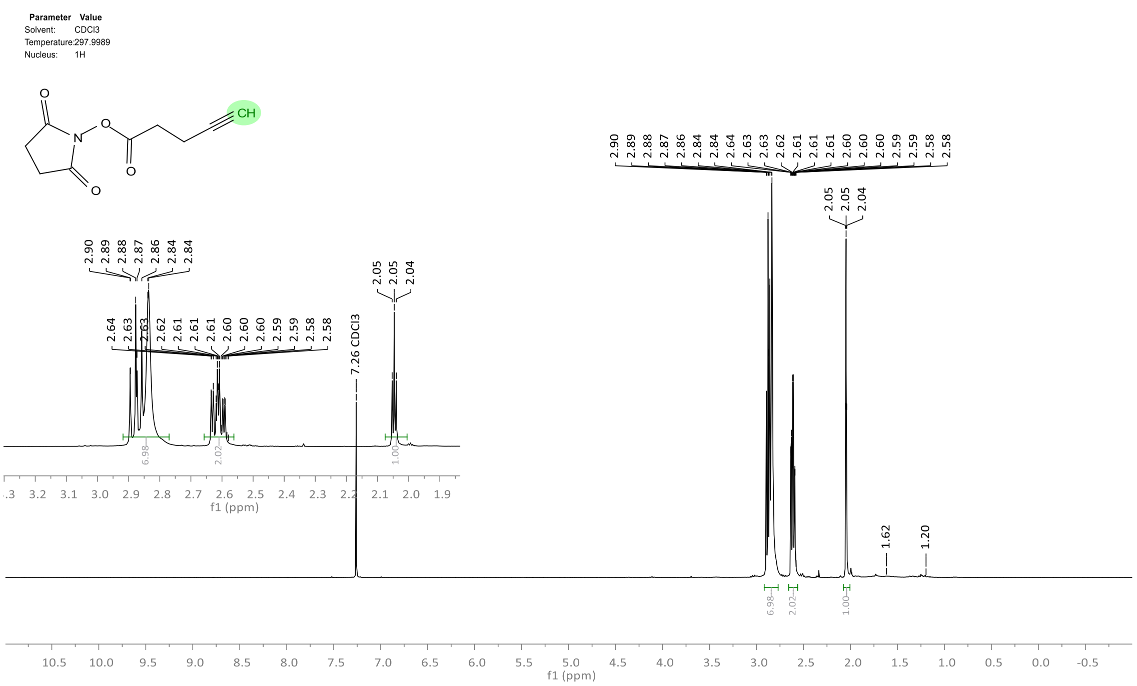

Parameter Value
Solvent: CDCl3
Temperature:297.9989
Nucleus: 1H
CH
7.26 CDCl3
1.62
1.20
f1 (ppm)

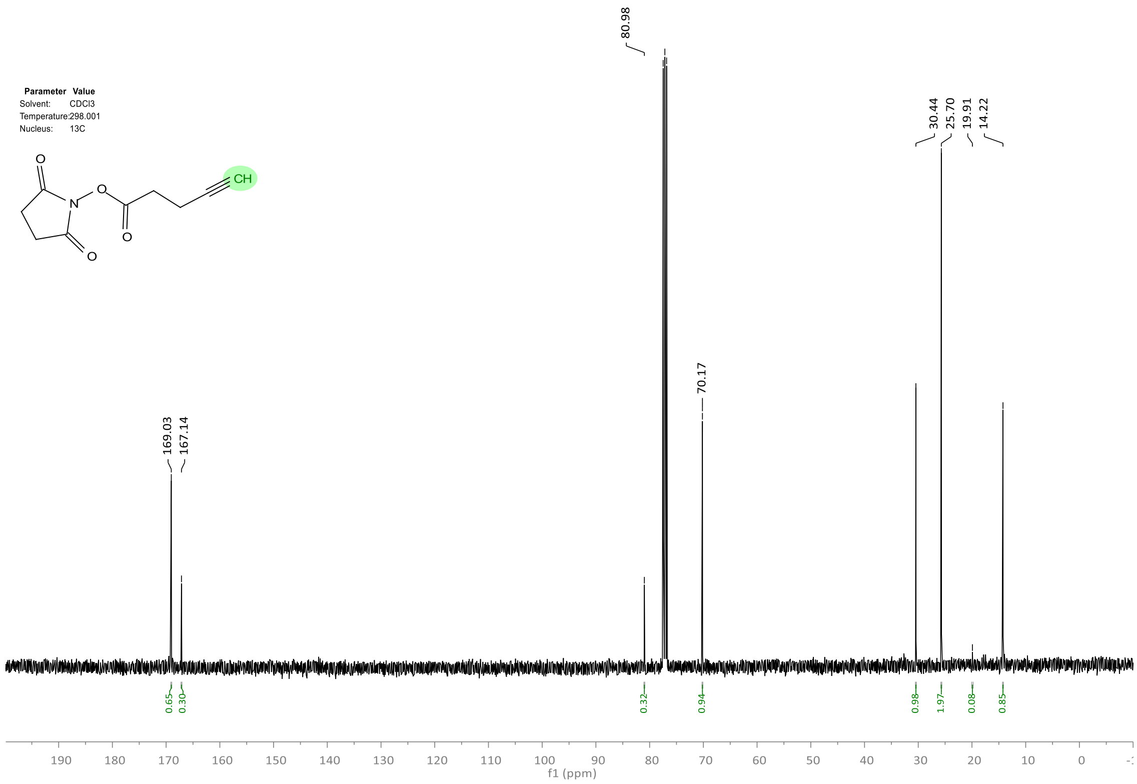

Parameter Value
Solvent: CDCl3
Temperature:298.001
Nucleus: 13C
CH
80.98
70.17
169.03
167.14
30.44
25.70
19.91
14.22
f1 (ppm)

Compound **9**

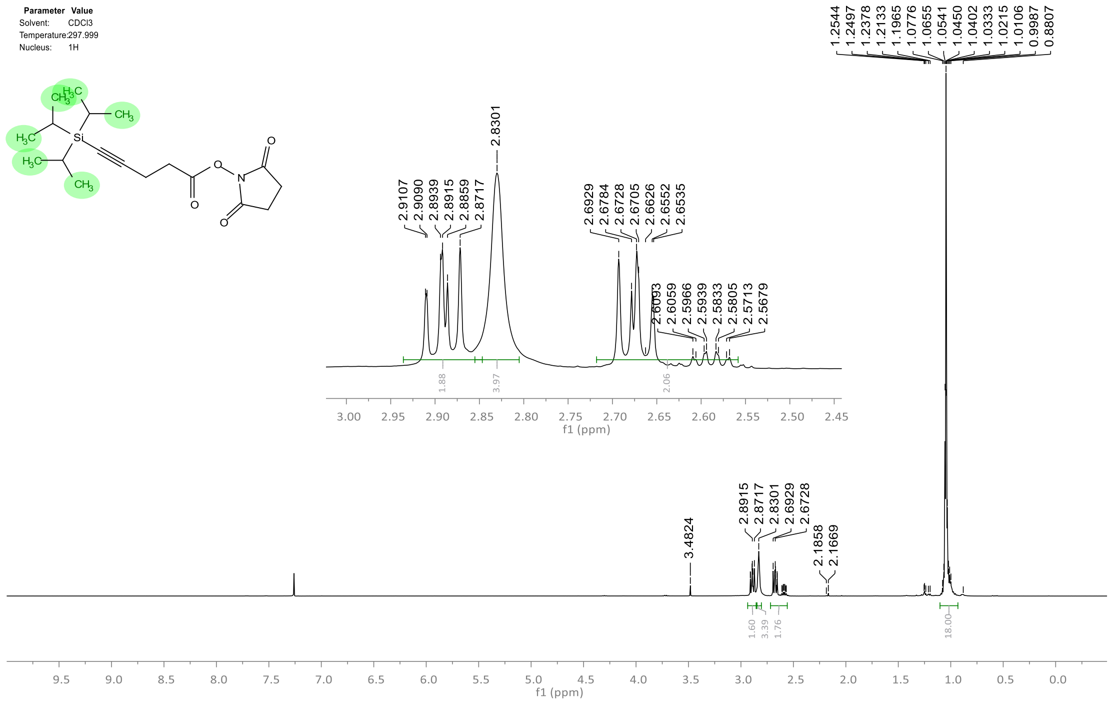
Parameter Value
Solvent: CDCl3
Temperature:297.999
Nucleus: 1H
f1 (ppm)

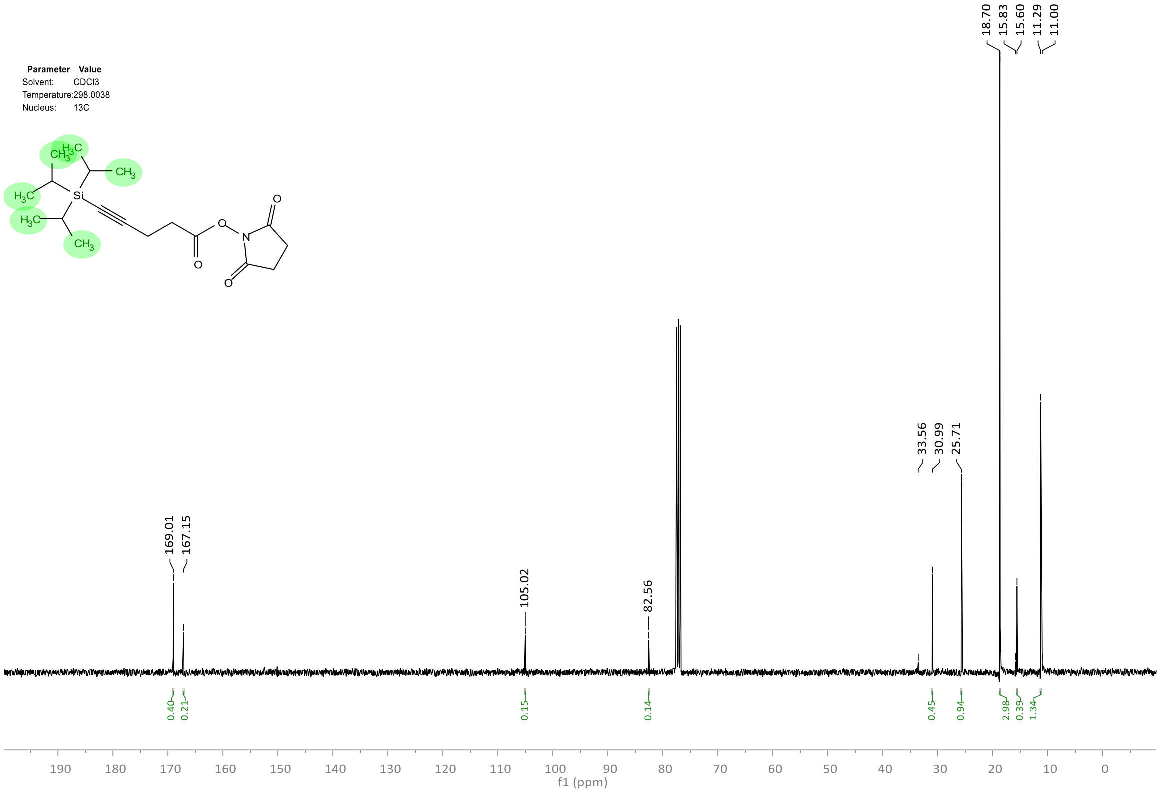
Parameter Value
Solvent: CDCl3
Temperature:298.0038
Nucleus: 13C
f1 (ppm)

Compound 10

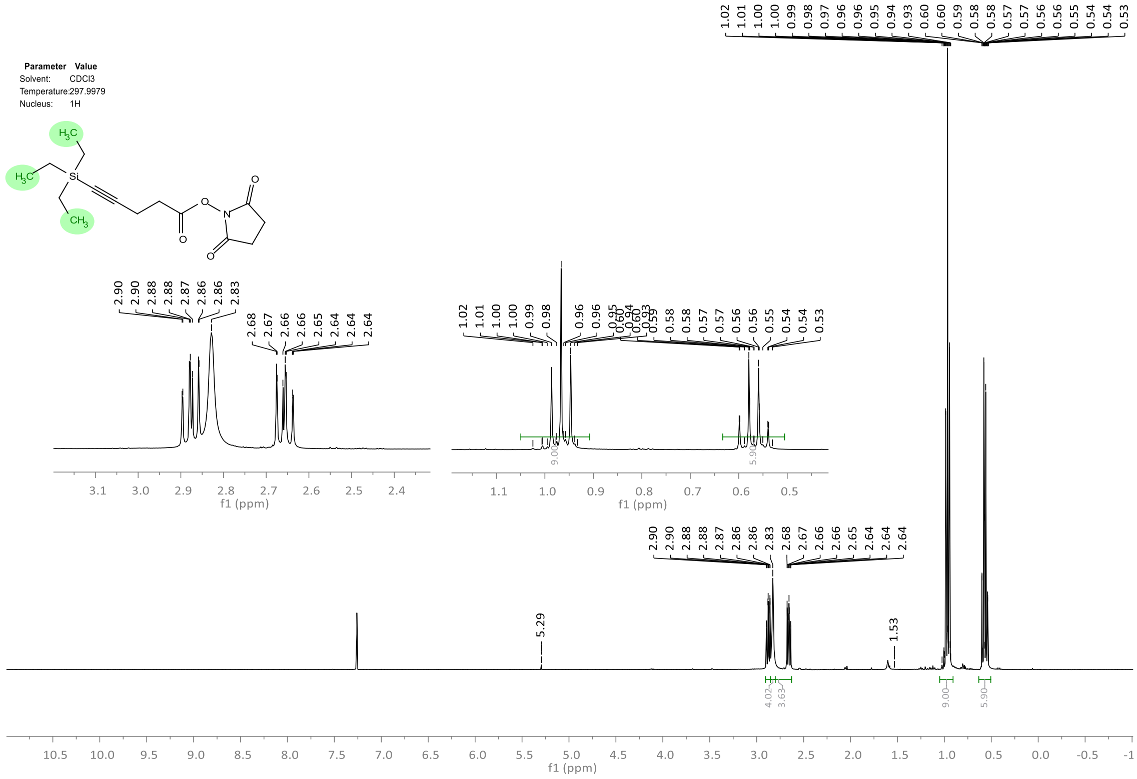
Parameter Value
Solvent: CDCl3
Temperature:297.9979
Nucleus: 1H
1.02 1.01 1.00 1.00 0.99 0.98 0.97 0.96 0.96 0.95 0.94 0.93 0.60 0.60 0.59 0.58 0.58 0.57 0.57 0.56 0.56 0.55 0.54 0.54 0.53
2.90 2.90 2.88 2.88 2.87 2.86 2.86 2.83 2.68 2.67 2.66 2.66 2.65 2.64 2.64 2.64
5.29
1.53
4.02
3.63
9.00
5.90
f1 (ppm)

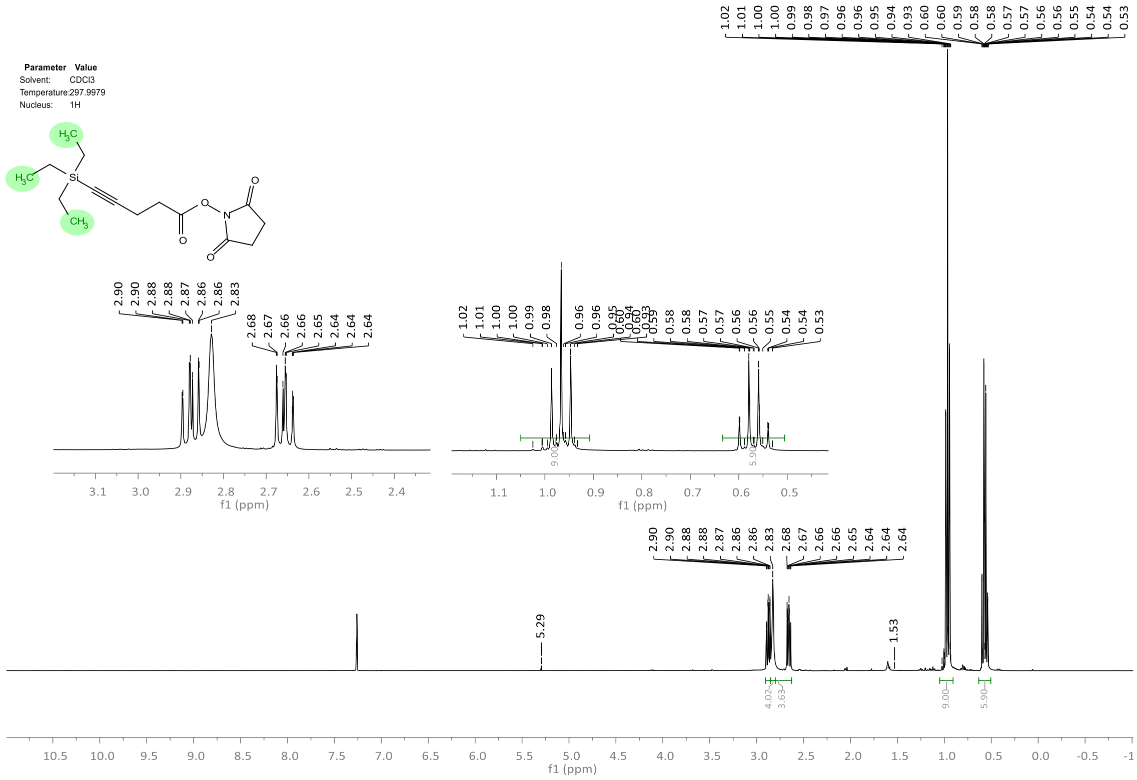
Parameter Value
Solvent: CDCl3
Temperature:297.9979
Nucleus: 1H
1.02 1.01 1.00 1.00 0.99 0.98 0.97 0.96 0.96 0.95 0.94 0.93 0.60 0.60 0.59 0.58 0.58 0.57 0.57 0.56 0.56 0.55 0.54 0.54 0.53
2.90 2.90 2.88 2.88 2.87 2.86 2.86 2.83 2.68 2.67 2.66 2.66 2.65 2.64 2.64 2.64
5.29
1.53
4.02
3.63
9.00
5.90
f1 (ppm)

Compound 1

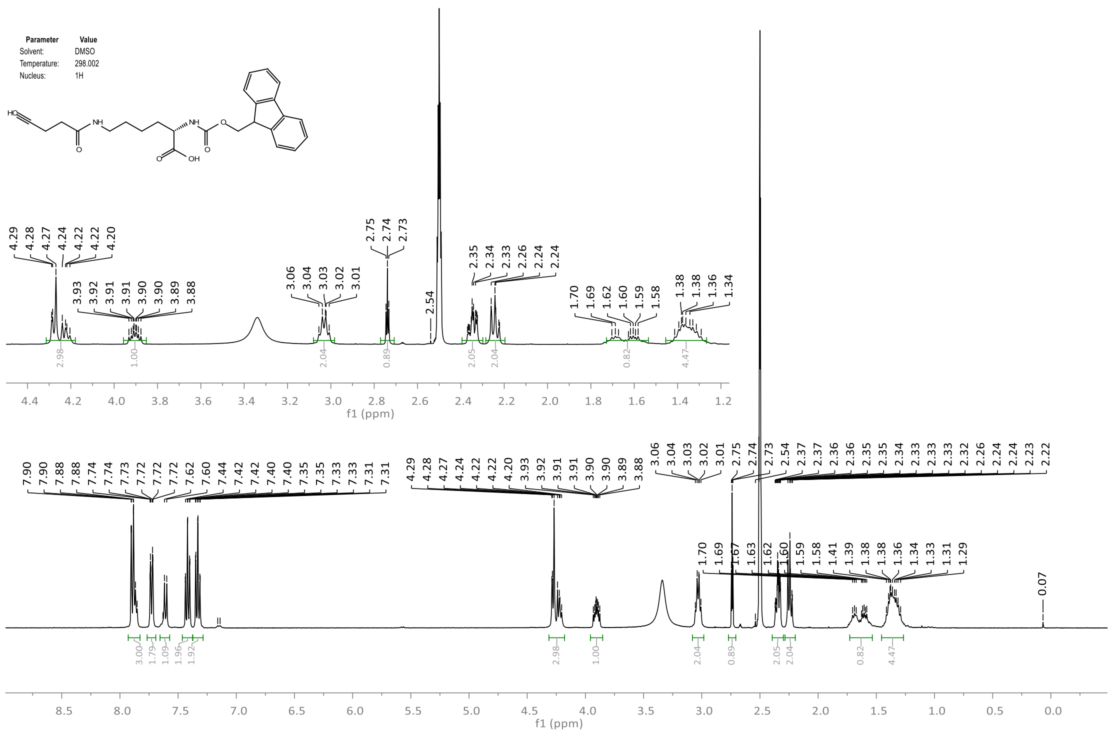

Parameter Value
Solvent: DMSO
Temperature: 298.002
Nucleus: 1H
f1 (ppm)

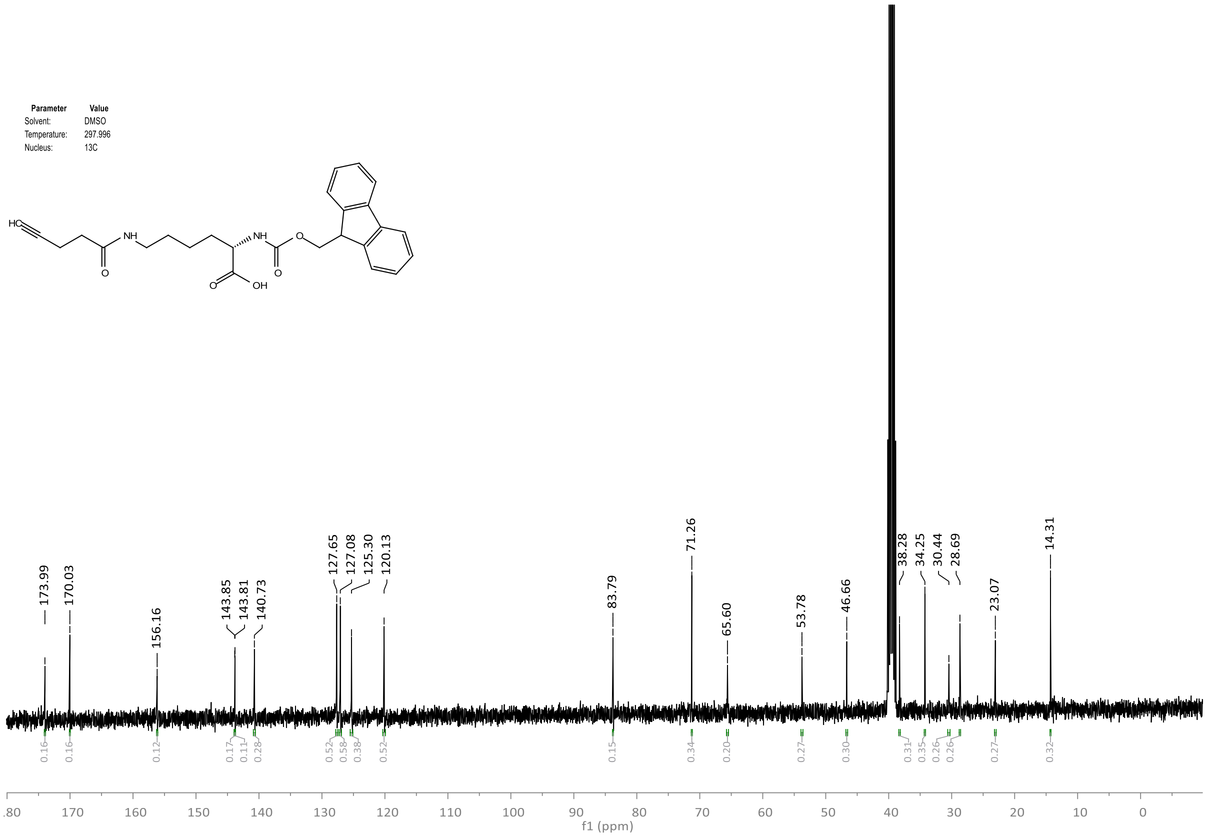

Parameter Value
Solvent: DMSO
Temperature: 297.996
Nucleus: 13C
173.99
170.03
156.16
143.85
143.81
140.73
127.65
127.08
125.30
120.13
83.79
71.26
65.60
53.78
46.66
38.28
34.25
30.44
28.69
23.07
14.31
f1 (ppm)

Compound **2**

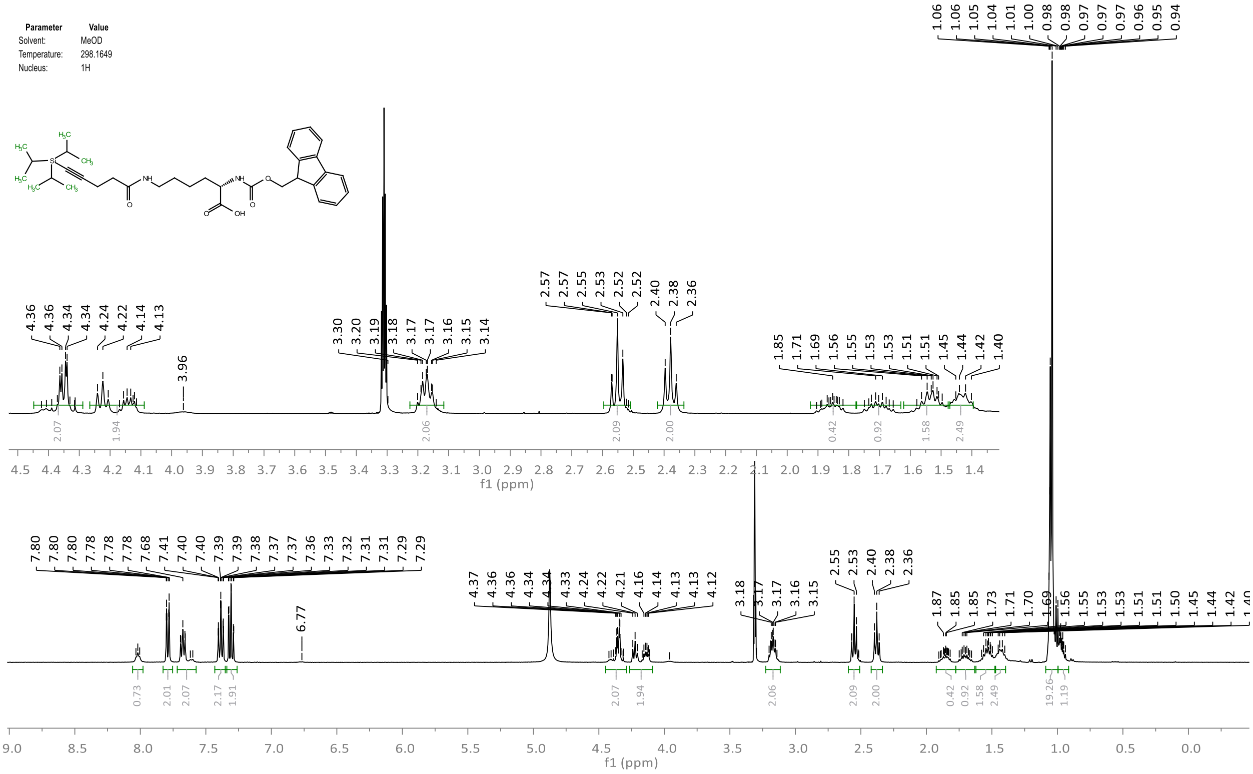
Parameter Value
Solvent: MeOD
Temperature: 298.1649
Nucleus: 1H
1.06 1.06 1.05 1.04 1.01 1.00 0.98 0.98 0.97 0.97 0.97 0.96 0.95 0.94
4.36 4.36 4.34 4.34 4.24 4.22 4.14 4.13
3.96
3.30 3.20 3.19 3.18 3.17 3.17 3.16 3.15 3.14
2.57 2.57 2.55 2.53 2.52 2.52 2.40 2.38 2.36
1.85 1.71 1.69 1.56 1.55 1.53 1.53 1.51 1.51 1.45 1.44 1.42 1.40
2.07 1.94 2.06 2.09 2.00 0.42 0.92 1.58 2.49
4.5 4.4 4.3 4.2 4.1 4.0 3.9 3.8 3.7 3.6 3.5 3.4 3.3 3.2 3.1 3.0 2.9 2.8 2.7 2.6 2.5 2.4 2.3 2.2 2.1 2.0 1.9 1.8 1.7 1.6 1.5 1.4
f1 (ppm)
7.80 7.80 7.80 7.78 7.78 7.78 7.68 7.41 7.40 7.40 7.39 7.39 7.38 7.37 7.37 7.36 7.33 7.32 7.31 7.31 7.29 7.29
6.77
4.37 4.36 4.36 4.34 4.34 4.33 4.24 4.22 4.21 4.16 4.14 4.13 4.13 4.12
3.18 3.17 3.17 3.16 3.15
2.55 2.53 2.40 2.38 2.36
1.87 1.85 1.85 1.73 1.71 1.70 1.69 1.56 1.55 1.53 1.53 1.51 1.51 1.50 1.45 1.44 1.42
0.73 2.01 2.07 2.17 1.91 2.07 1.94 2.06 2.09 2.00 0.42 0.92 1.58 2.49 19.26 1.19
9.0 8.5 8.0 7.5 7.0 6.5 6.0 5.5 5.0 4.5 4.0 3.5 3.0 2.5 2.0 1.5 1.0 0.5 0.0
f1 (ppm)

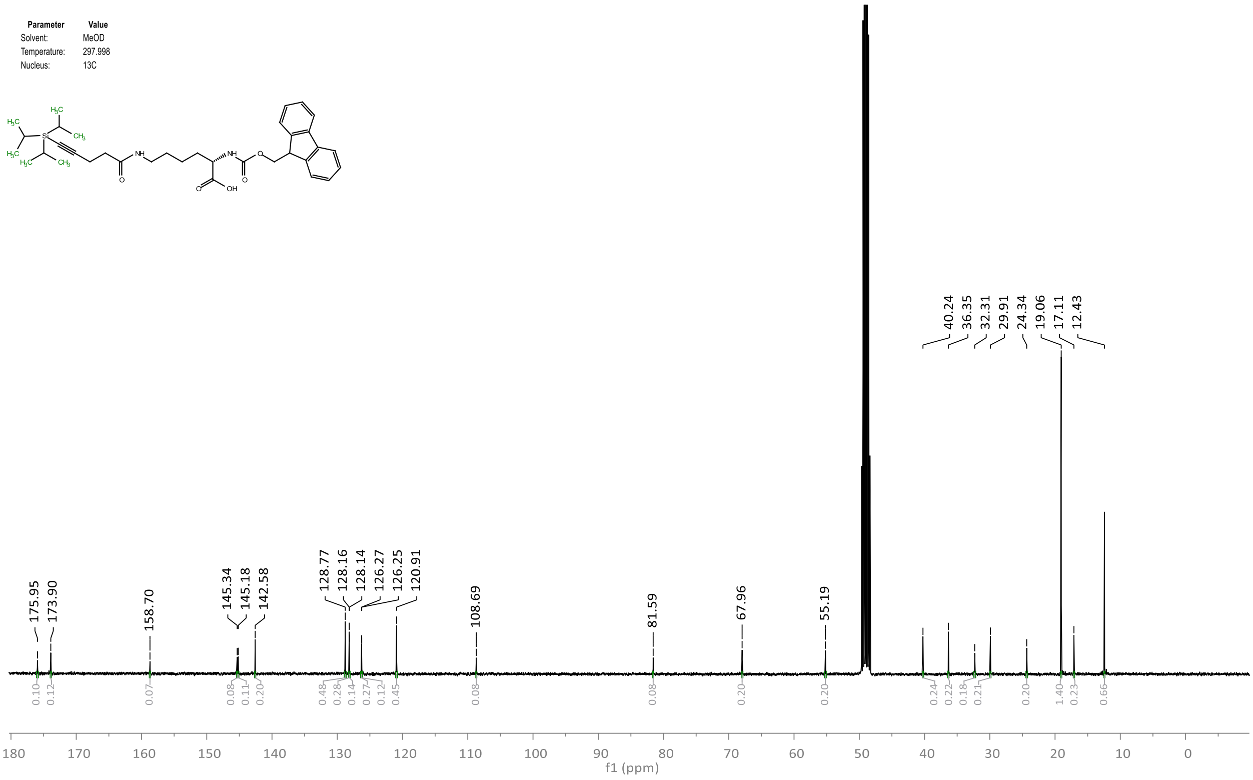
Parameter Value
Solvent: MeOD
Temperature: 297.998
Nucleus: 13C
40.24 36.35 32.31 29.91 24.34 19.06 17.11 12.43
175.95 173.90 158.70 145.34 145.18 142.58 128.77 128.16 128.14 126.27 126.25 120.91 108.69 81.59 67.96 55.19
0.10 0.12 0.07 0.08 0.11 0.20 0.48 0.28 0.14 0.27 0.12 0.45 0.08 0.08 0.20 0.20 0.24 0.22 0.18 0.21 0.20 1.40 0.23 0.66
180 170 160 150 140 130 120 110 100 90 80 70 60 50 40 30 20 10 0
f1 (ppm)

Compound 3

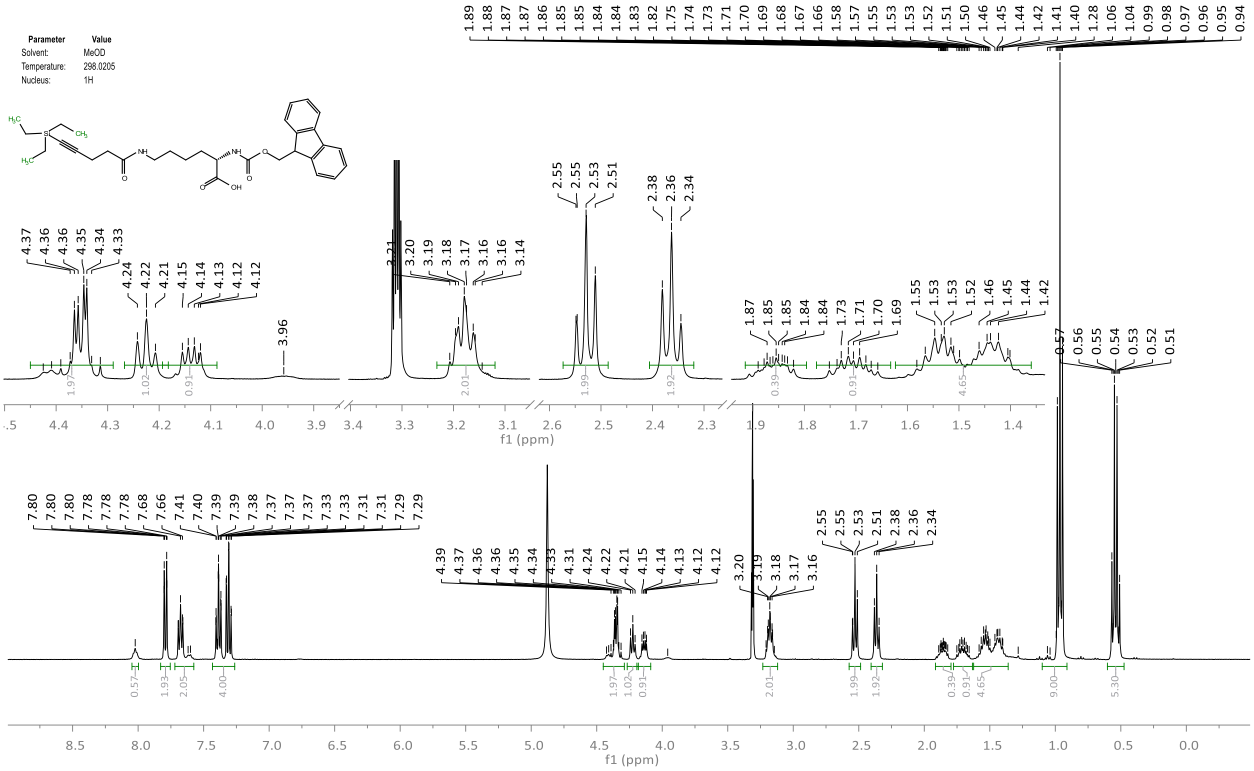
Parameter Value
Solvent: MeOD
Temperature: 298.0205
Nucleus: 1H
f1 (ppm)

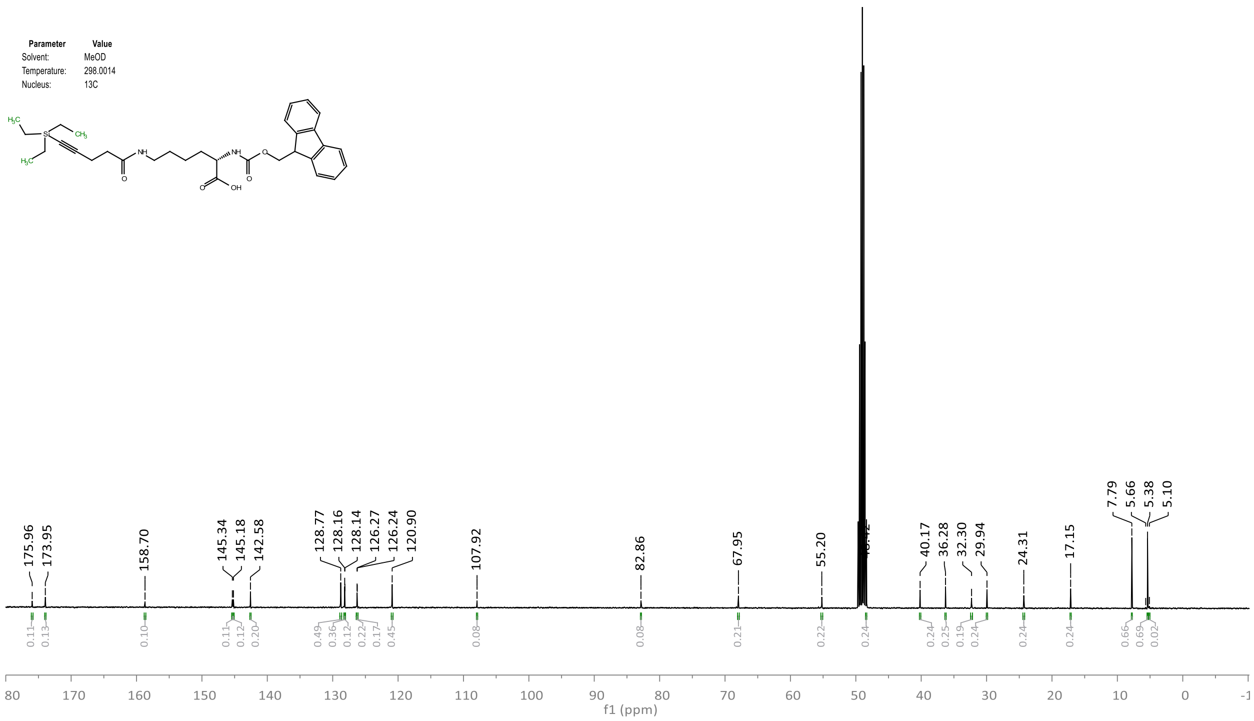
Parameter Value
Solvent: MeOD
Temperature: 298.0014
Nucleus: 13C
f1 (ppm)

Compound **12**

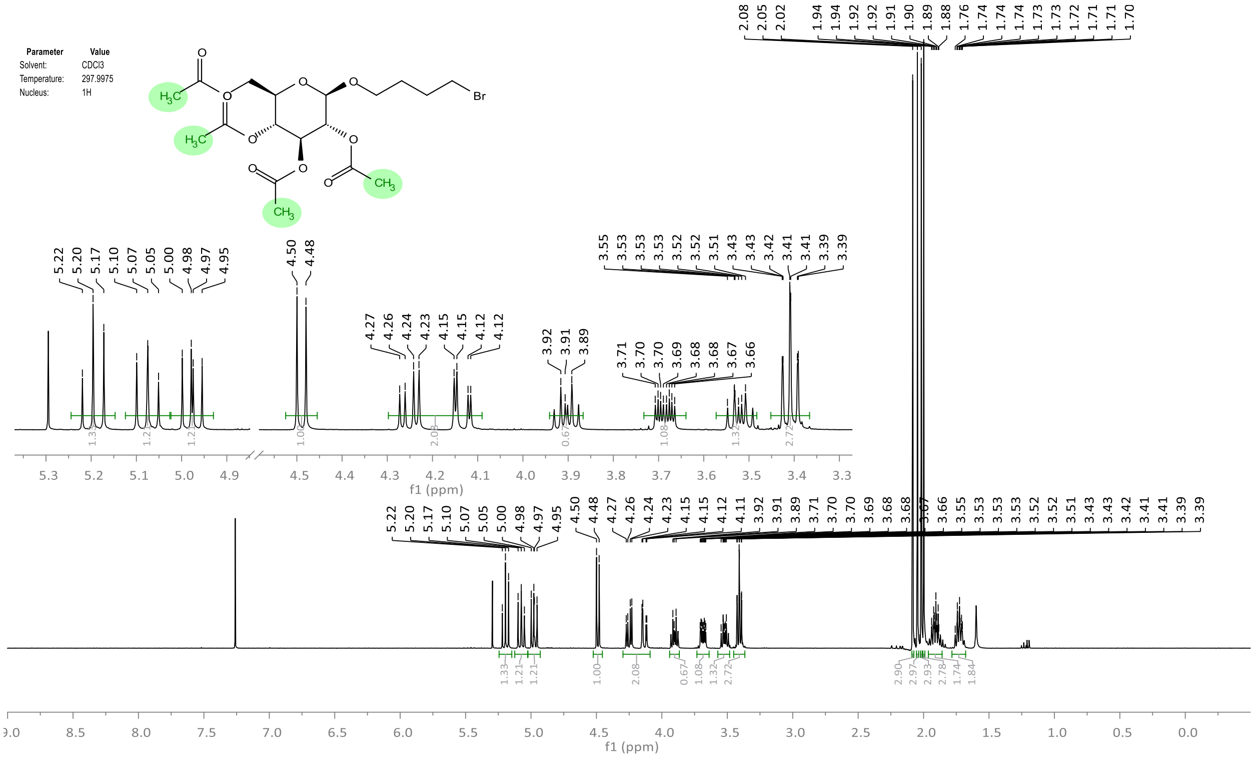

Parameter Value
Solvent: CDCl3
Temperature: 297.9975
Nucleus: 1H
Br
f1 (ppm)

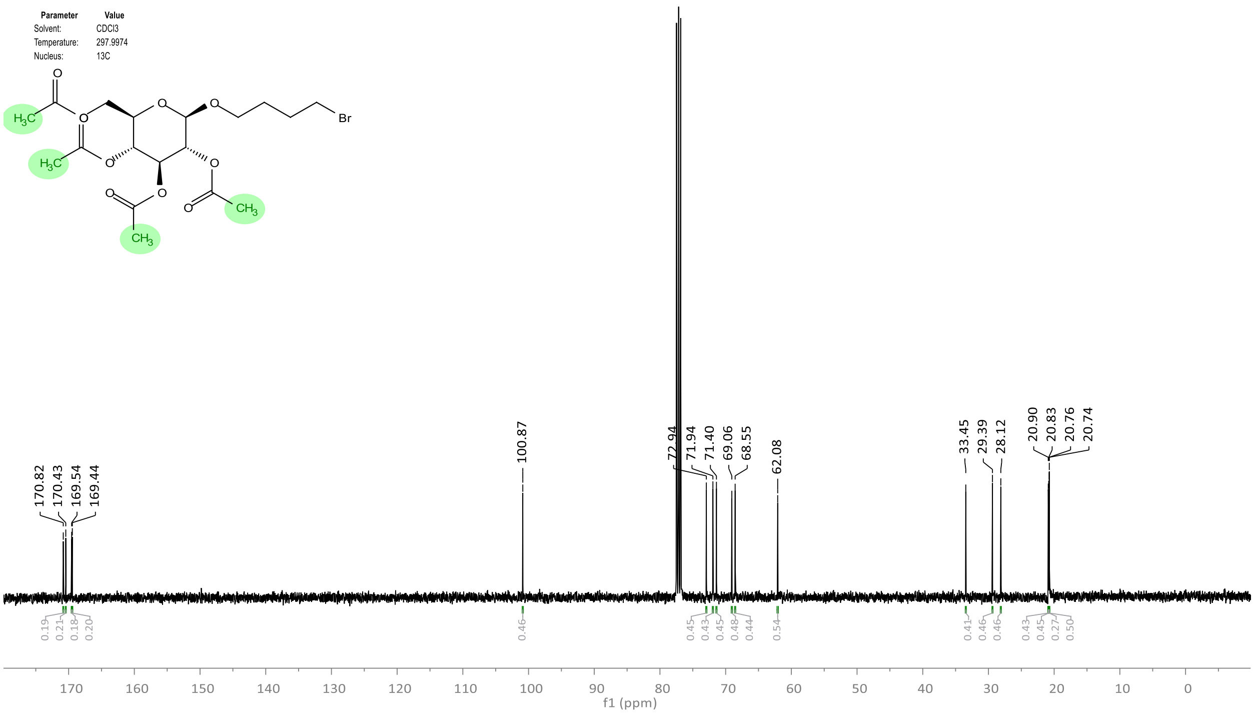

Parameter Value
Solvent: CDCl3
Temperature: 297.9974
Nucleus: 13C
Br
170.82
170.43
169.54
169.44
100.87
72.94
71.94
71.40
69.06
68.55
62.08
33.45
29.39
28.12
20.90
20.83
20.76
20.74
f1 (ppm)

Compound **14**

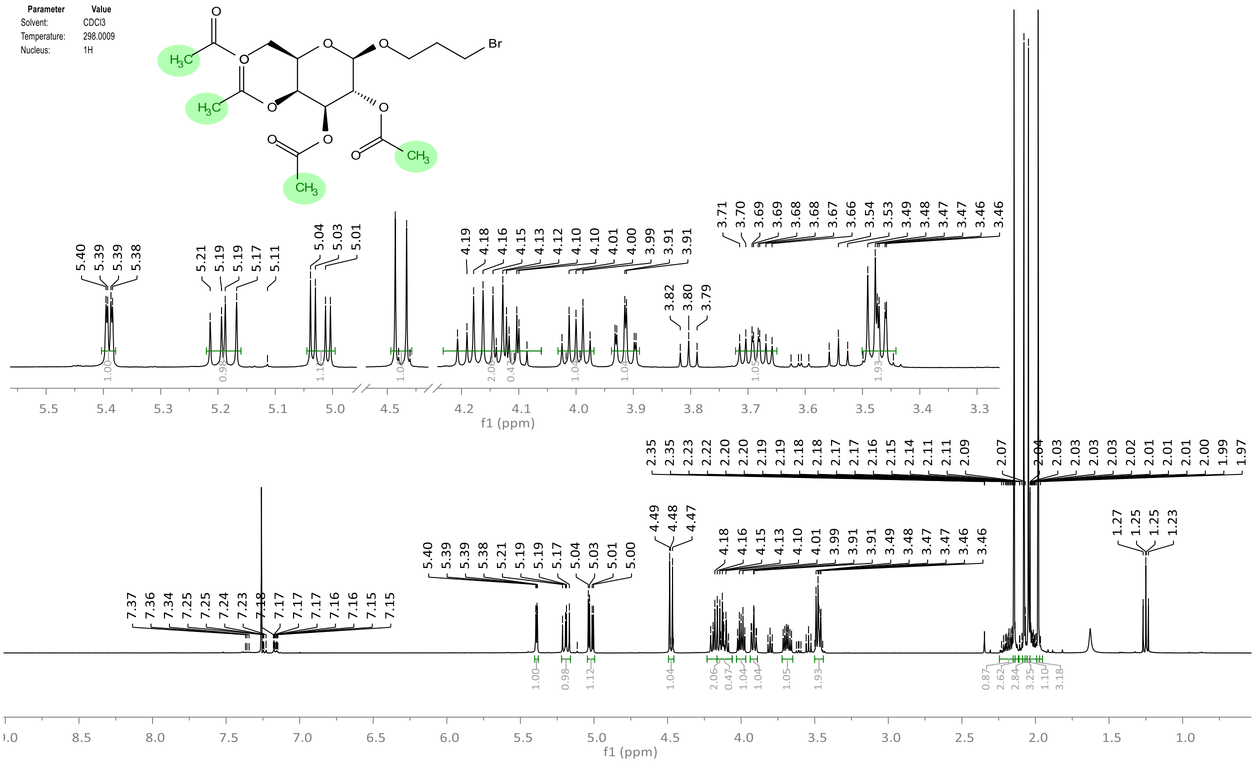
Parameter Value
Solvent: CDCl3
Temperature: 298.0009
Nucleus: 1H
O
Br
H3C
CH3
f1 (ppm)

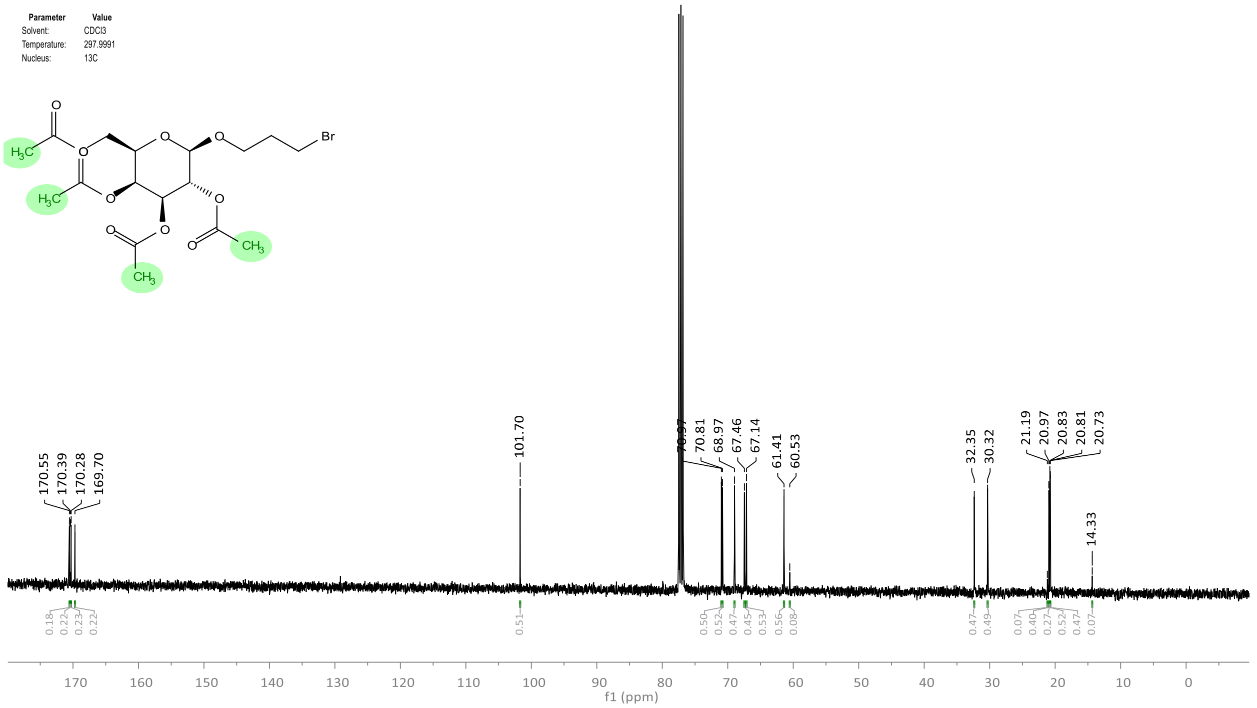
Parameter Value
Solvent: CDCl3
Temperature: 297.9991
Nucleus: 13C
O
Br
H3C
CH3
f1 (ppm)

Compound **13**

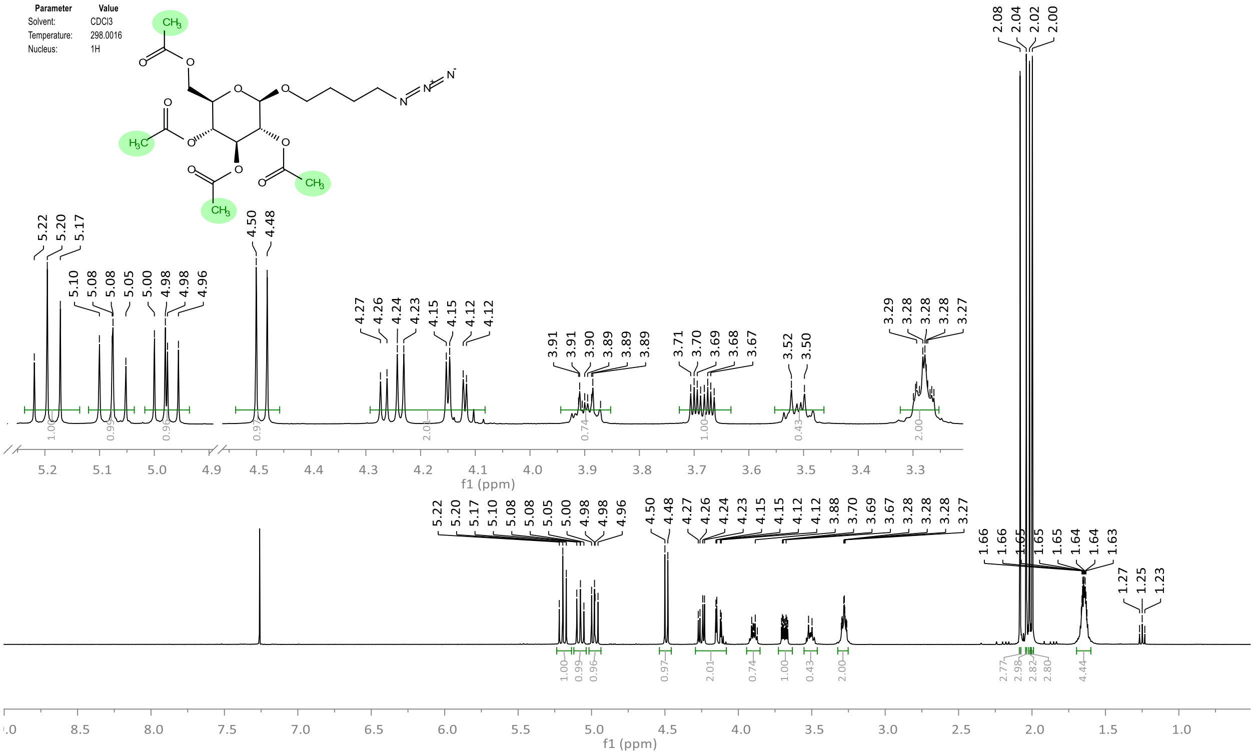
Parameter Value
Solvent: CDCl3
Temperature: 298.0016
Nucleus: 1H
f1 (ppm)

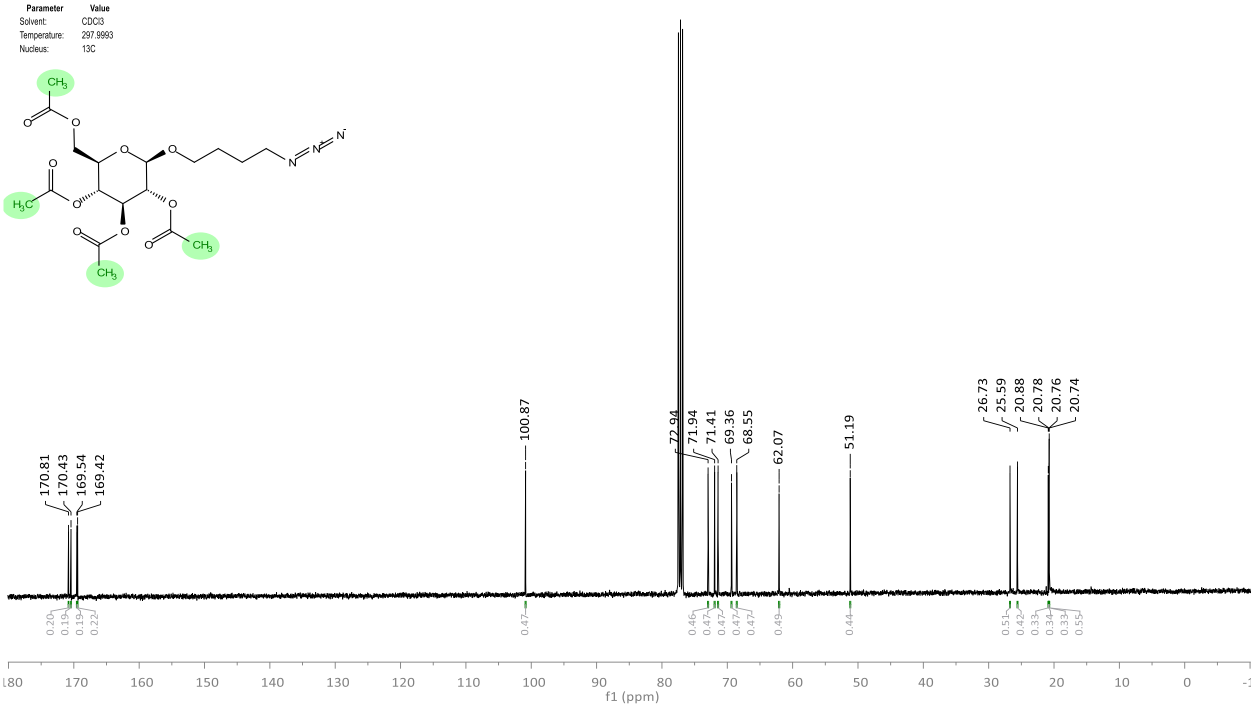
Parameter Value
Solvent: CDCl3
Temperature: 297.9993
Nucleus: 13C
170.81 170.43 169.54 169.42
100.87
72.94 71.94 71.41 69.36 68.55 62.07 51.19
26.73 25.59 20.88 20.78 20.76 20.74
f1 (ppm)

Compound **17**

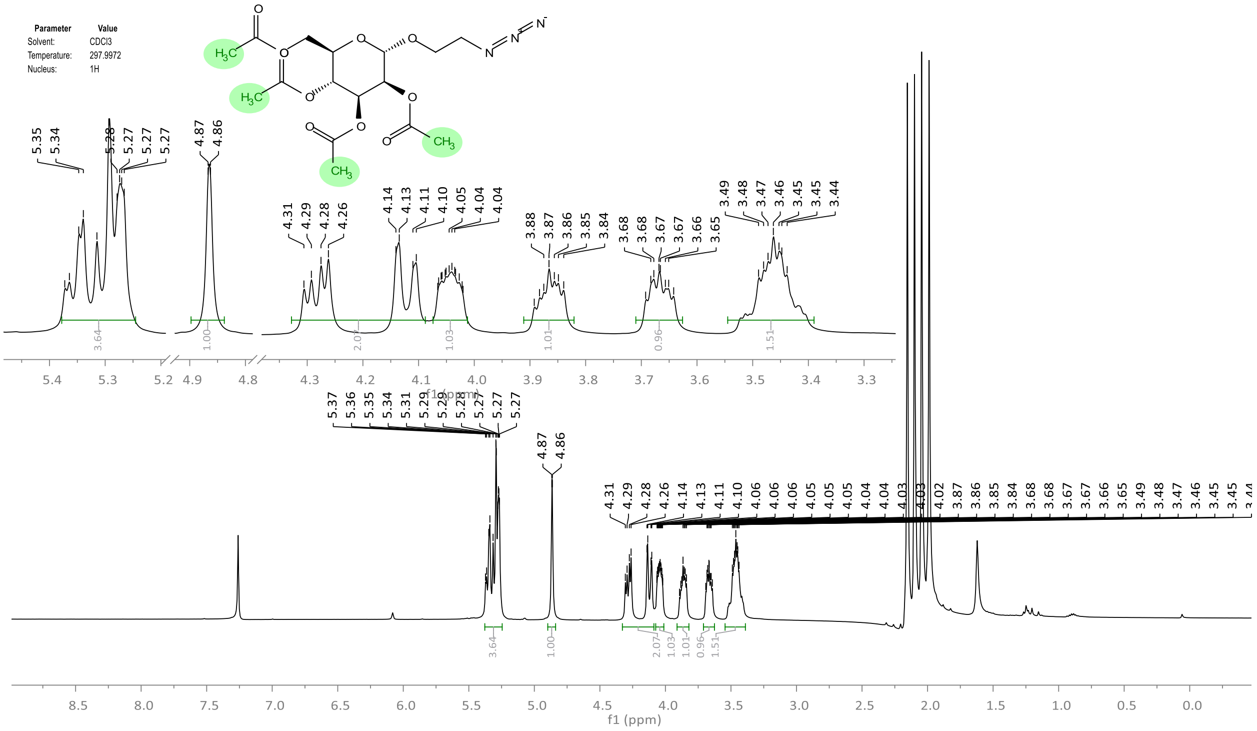

Parameter Value
Solvent: CDCl3
Temperature: 297.9972
Nucleus: 1H
f1 (ppm)

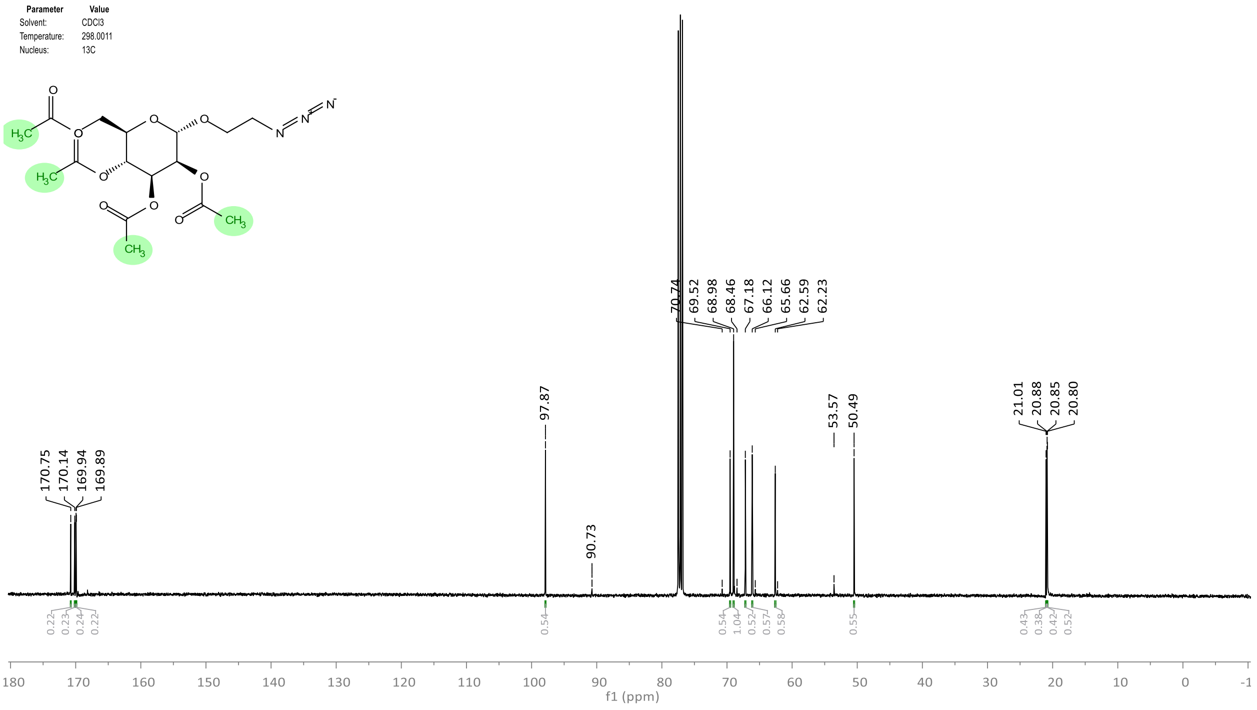

Parameter Value
Solvent: CDCl3
Temperature: 298.0011
Nucleus: 13C
f1 (ppm)

Compound **15**

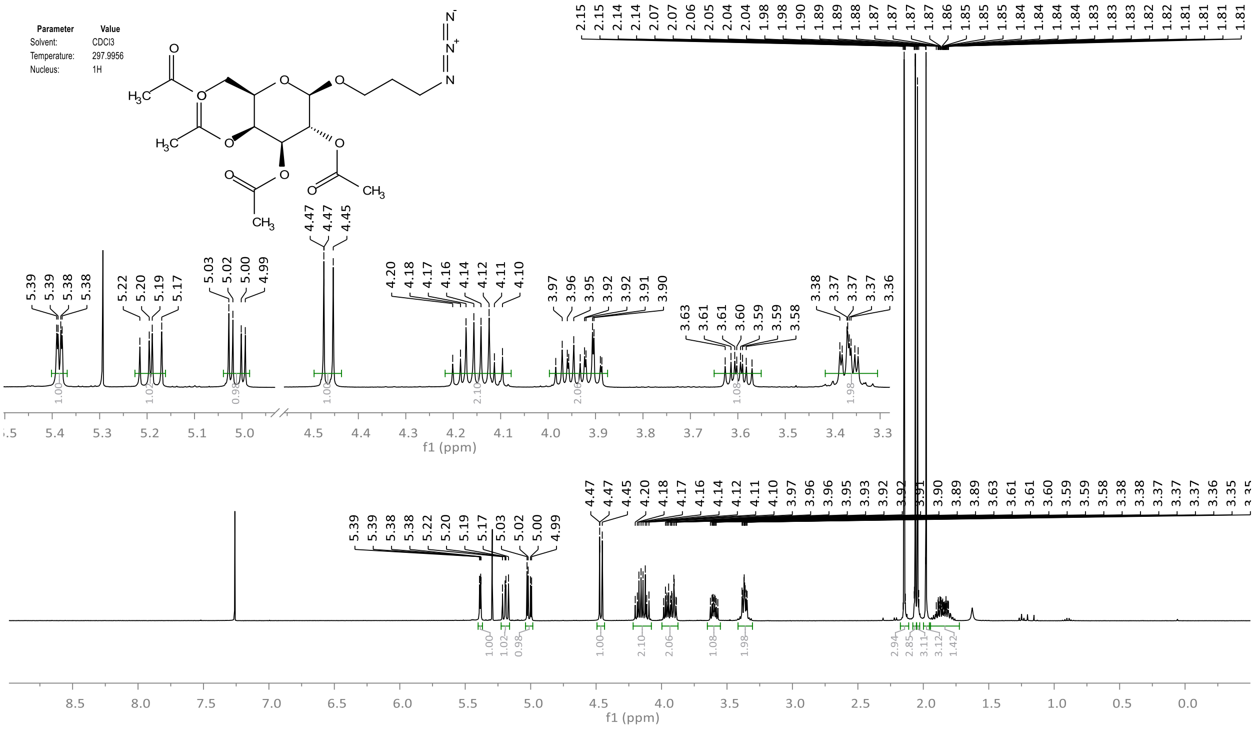
Parameter Value
Solvent: CDCl3
Temperature: 297.9956
Nucleus: 1H
f1 (ppm)

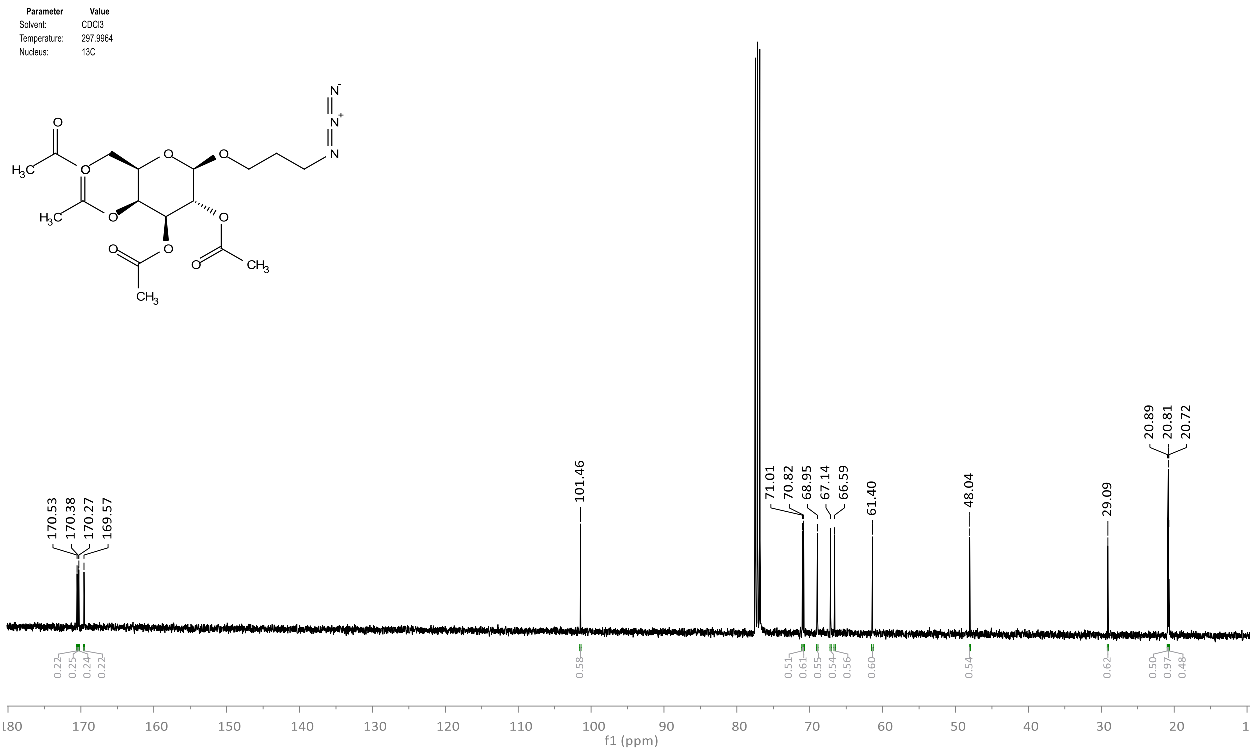
Parameter Value
Solvent: CDCl3
Temperature: 297.9964
Nucleus: 13C
f1 (ppm)

Compound Glc-C4-N_3

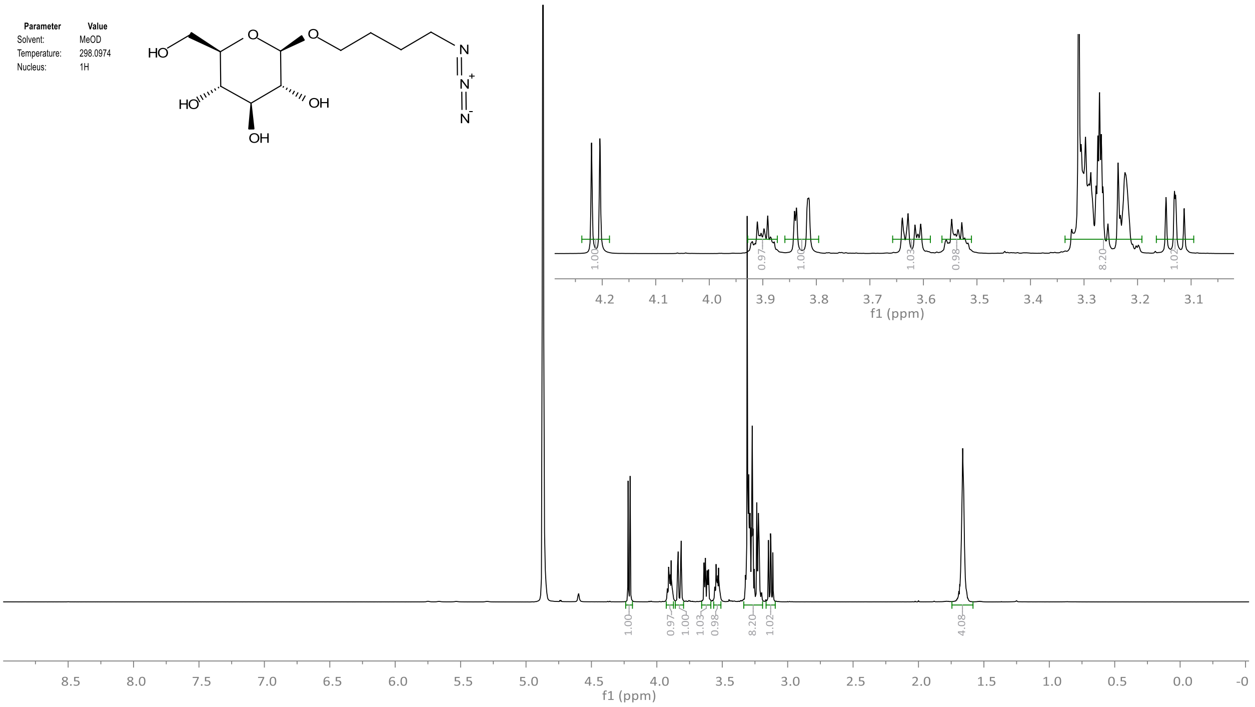

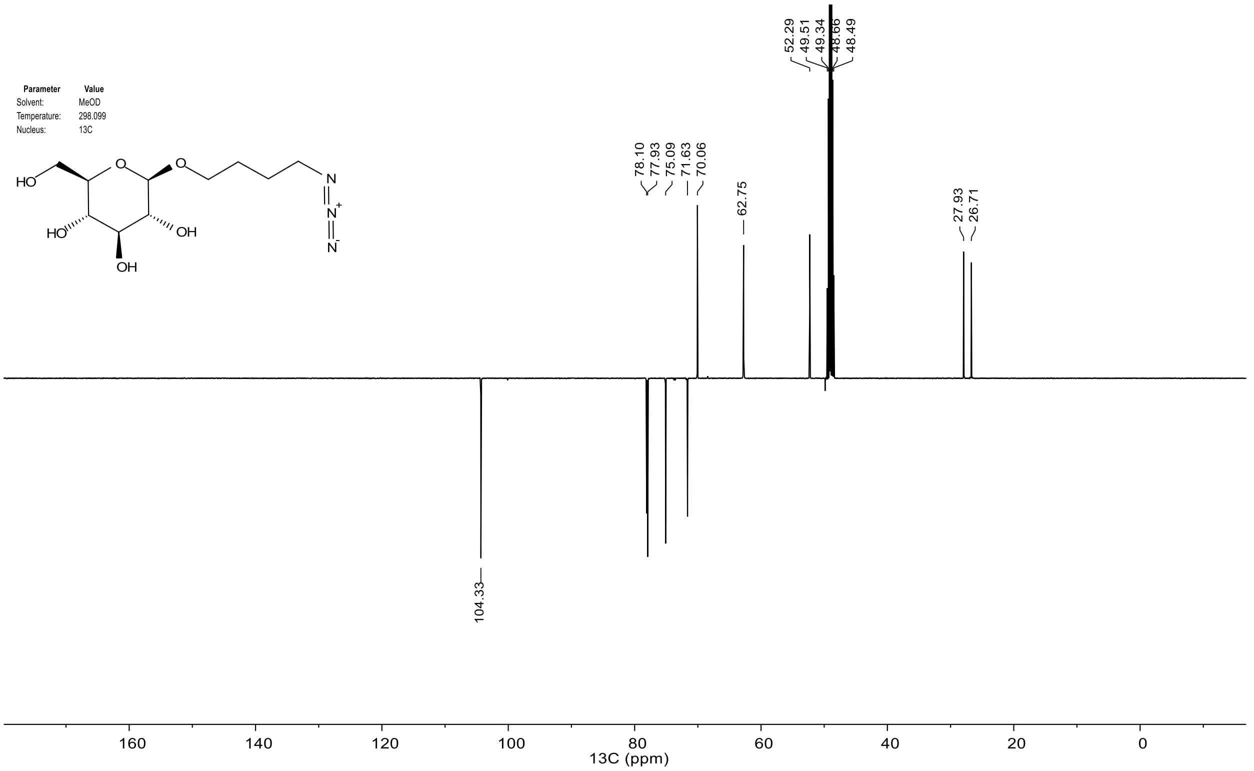

Compound Man-C2-N_3

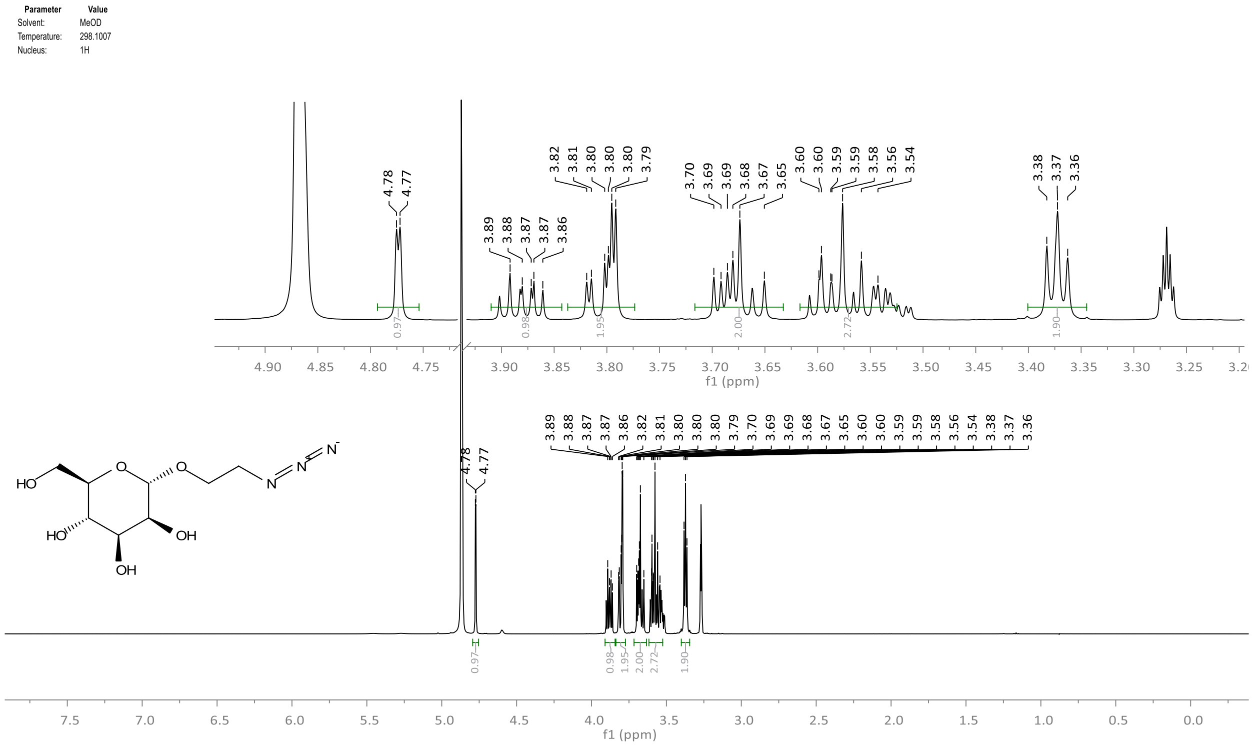

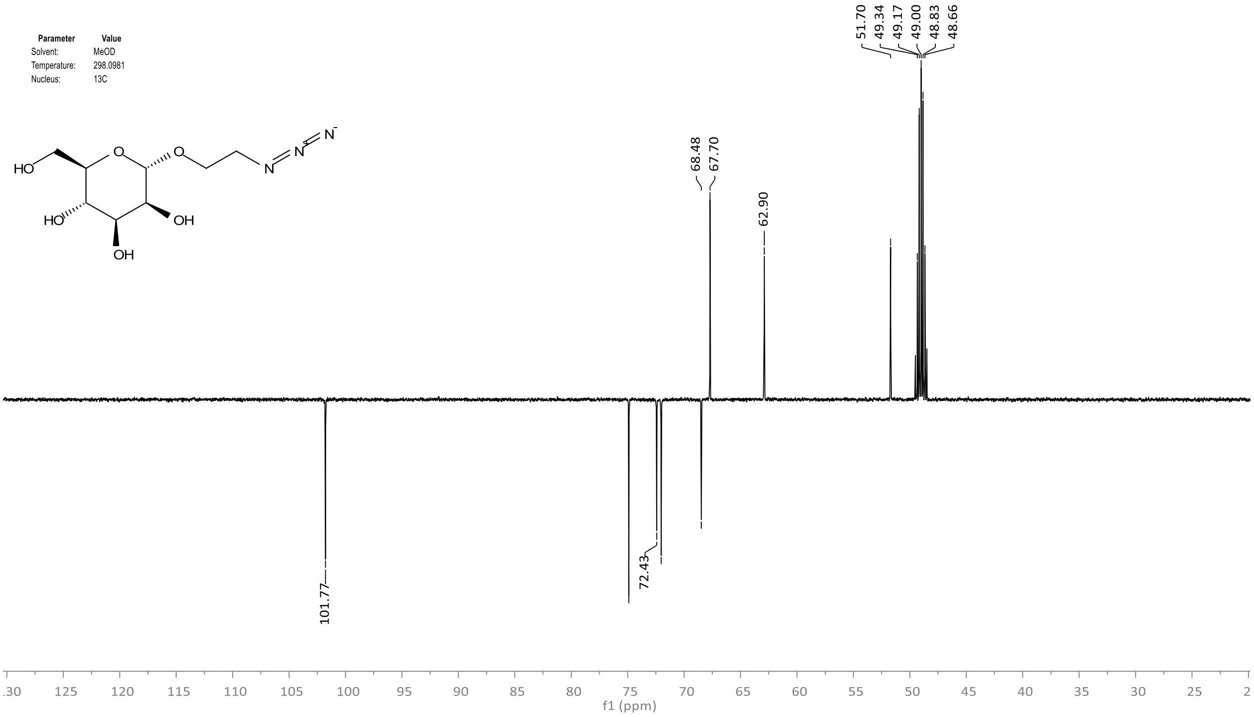

Compound Gal-C3-N_3

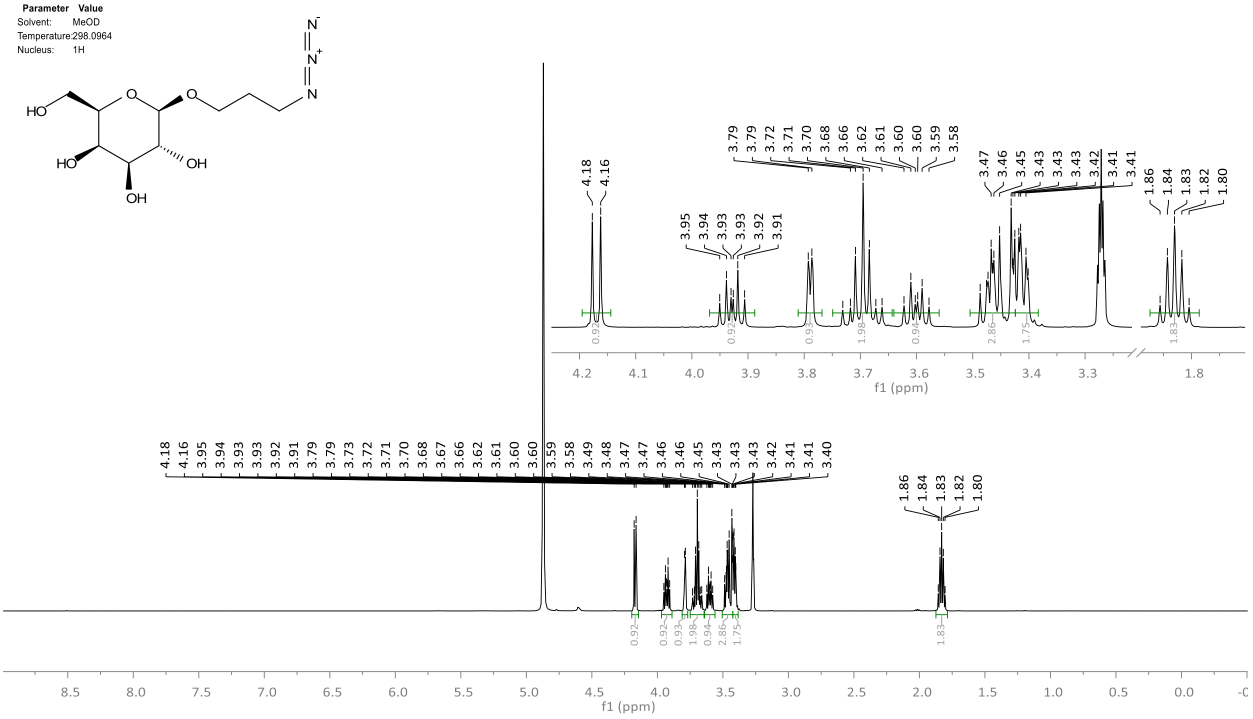

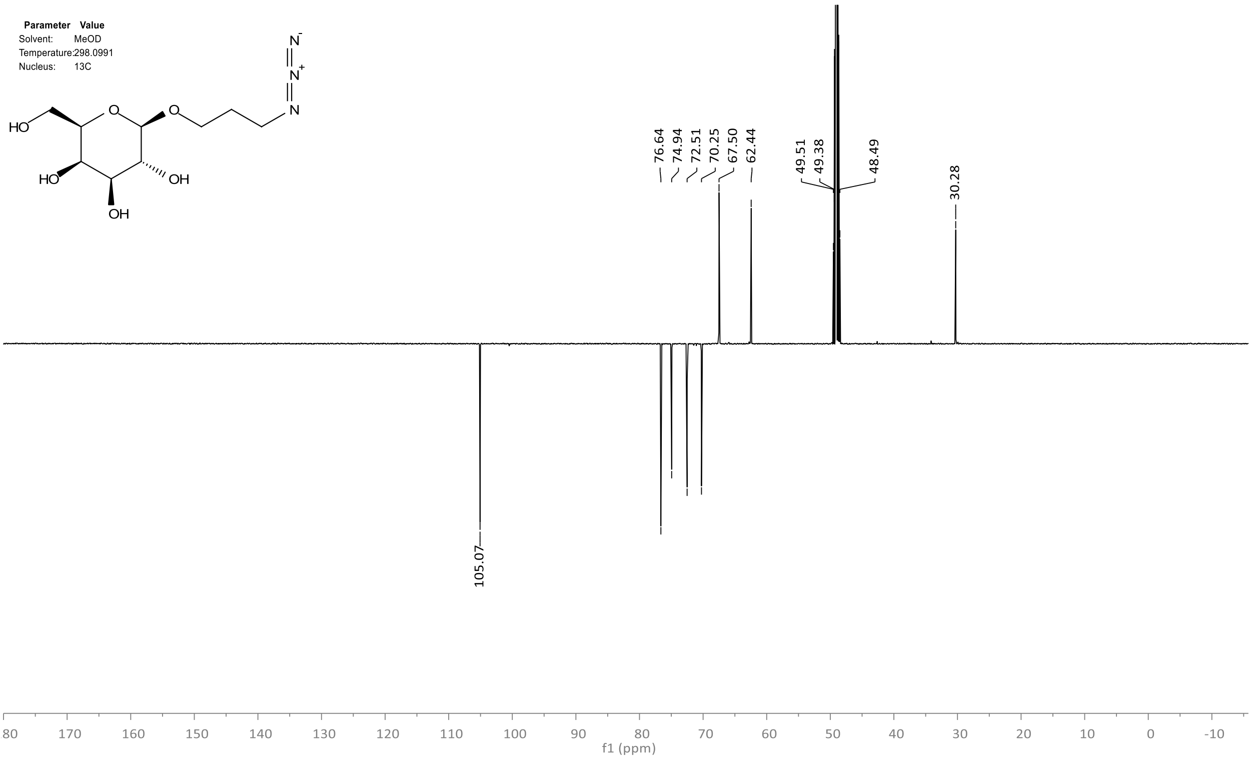

Abbreviations:

ACN	Acetonitrile
Boc	*tert*-Butyloxycarbonyl
BTTP	3-[4-({Bis[(1-tert-butyl-1H-1,2,3-triazol-4-yl)methyl]amino}methyl)-1H-1,2,3-triazol-1-yl]propanol
Bu	Butyl
CuAAC	The Copper(I)-Catalyzed Azide Alkyne Cycloaddition
DCC	*N*,*N*′-Dicyclohexylcarbodiimide
DCM	Dichloromethane
DIC	*N,N′*-Diisopropylcarbodiimide
DIPEA	*N*,*N*-Diisopropylethylamine
DMAP	4-(Dimethylamino)pyridine
DMF	Dimethylformamide
DMSO	Dimethyl sulfoxide
EDTA	Ethylenediaminetetraacetic acid
Eq.	Equivalent
Fmoc	9-Fluorenylmethoxycarbonyl
HBTU	*N,N,N′,N′*-Tetramethyl-*O*-(1*H*-benzotriazol-1-yl)uronium hexafluorophosphate
HEPES	4-(2-Hydroxyethyl)piperazine-1-ethanesulfonic acid
HPLC	High Pressure Liquid Chromatography
HRMS	High Resolution Mass Spectroscopy
iPr	Isopropyl
iPrOH	2-Propanol
LC-MS	Liquid Chromatography – Mass Spectroscopy
Me	Methyl
NHS	N-Hydroxysuccinimide
NMM	4-Methylmorpholine
NMR	Nuclear Magnetic Resonance
Pbf	2,2,4,6,7-Pentamethyldihydrobenzofuran-5-sulfonyl
PE	Petrolether
SPPS	Solid Phase Peptide Synthesis
TBAF	Tetrabutylammonium fluoride
t-Bu	*Tert*-Butyl
t-BuOH	2-Methyl-2-propanol
TES	Triethylsilyl
TFA	Trifluoroacetic acid
THF	Tetrahydrofuran
TIPS	Triisopropylsilyl
TIS	Triisopropylsilane
Trt	Trityl

References:

1. G. M. Fischer, C. Jungst, M. Isomaki-Krondahl, D. Gauss, H. M. Moller, E. Daltrozzo and A. Zumbusch, *Chem Commun*, 2010, **46**, 5289-5291.
2. B. Neises and W. Steglich, *Angewandte Chemie International Edition in English*, 1978, **17**, 522-524.
3. P. R. Werkhoven, M. Elwakiel, T. J. Meuleman, H. C. Q. van Ufford, J. A. W. Kruijtzer and R. M. J. Liskamp, *Org Biomol Chem*, 2016, **14**, 701-710.
4. S. Combemale, J. N. Assam-Evoung, S. Houaidji, R. Bibi and V. Barragan-Montero, *Molecules*, 2014, **19**, 1120-1149.
5. M. Malkoch, K. Schleicher, E. Drockenmuller, C. J. Hawker, T. P. Russell, P. Wu and V. V. Fokin, *Macromolecules*, 2005, **38**, 3663-3678.
6. A. Horatscheck, S. Wagner, J. Ortwein, B. G. Kim, M. Lisurek, S. Beligny, A. Schutz and J. Rademann, *Angew. Chem. Int. Ed. Engl.*, 2012, **51**, 9441-9447.
7. K. Shinoda, Y. Sohma and M. Kanai, *Bioorg Med Chem Lett*, 2015, **25**, 2976-2979.
8. J. Dahmen, T. Frejd, G. Gronberg, T. Lave, G. Magnusson and G. Noori, *Carbohyd Res*, 1983, **116**, 303-307.
9. V. Ladmiral, G. Mantovani, G. J. Clarkson, S. Cauet, J. L. Irwin and D. M. Haddleton, *J Am Chem Soc*, 2006, **128**, 4823-4830.
10. S. Park and I. Shin, *Org Lett*, 2007, **9**, 1675-1678.
11. C. C. Lee, G. Grandinetti, P. M. McLendon and T. M. Reineke, *Macromol Biosci*, 2010, **10**, 585-598.
12. B. Ren, M. Y. Wang, J. Y. Liu, J. T. Ge, X. L. Zhang and H. Dong, *Green Chem*, 2015, **17**, 1390-1394.
13. E. Calatrava-Perez, S. A. Bright, S. Achermann, C. Moylan, M. O. Senge, E. B. Veale, D. C. Williams, T. Gunnlaugsson and E. M. Scanlan, *Chem Commun (Camb)*, 2016, **52**, 13086-13089.
14. M. Gude, J. Ryf and P. D. White, *Lett Pept Sci*, 2002, **9**, 203-206.
15. I. Coin, M. Beyermann and M. Bienert, *Nat Protoc*, 2007, **2**, 3247-3256.
16. I. E. Valverde, A. F. Delmas and V. Aucagne, *Tetrahedron*, 2009, **65**, 7597-7602.
